# Do Maternal Living Arrangements Influence the Vaccination Status of Children Age 12–23 Months? A Data Analysis of Demographic Health Surveys 2010–11 from Zimbabwe

**DOI:** 10.1371/journal.pone.0132357

**Published:** 2015-07-13

**Authors:** Rodolfo Rossi

**Affiliations:** London School of Hygiene & Tropical Medicine, London, United Kingdom; Örebro University, SWEDEN

## Abstract

**Introduction:**

Although vaccination is an effective intervention to reduce childhood mortality and morbidity, reasons for incomplete vaccination, including maternal living arrangements, have been marginally explored. This study aims at assessing whether maternal living arrangements are associated with vaccination status of children aged 12–23 months in Zimbabwe. It also explores other variables that may be associated with having children not fully vaccinated.

**Materials and Methods:**

A cross-sectional analysis was performed on the DHS-VI done in Zimbabwe in 2010–2011 (response rate 93%). Incomplete vaccination of children (outcome), was defined as not having received one dose of BCG and measles, 3 doses of polio and DPT/Pentavalent. Maternal living arrangements (main exposure), and other exposure variables were analysed. Survey logistic regression was used to calculate crude and adjusted OR for exposures against the outcome.

**Results:**

The dataset included 1,031 children aged 12–23 months. 65.8% of children were fully vaccinated. 65.7% of the mothers were married and cohabitating with a partner, 20.3% were married/partnered but living separately and 14% were not married. Maternal living arrangements were not associated with the vaccination status of children both in crude and adjusted analysis. Factors associated with poorer vaccination status of the children included: no tetanus vaccination for mothers during pregnancy (adjusted OR = 2.1, 95%CI 1.5;3.0), child living away from mother (adjusted OR = 1.5, 95%CI 1.2;1.8), mother’s education (adjusted OR = 0.6, 95%CI 0.4;0.9), high number of children living in the household (adjusted OR = 1.5, 95%CI 1.1;2.2), child age (adjusted OR = 0.7, 95%CI 0.5;0.9).

**Discussion:**

Maternal living arrangements were not associated with vaccination status of Zimbabwean children. Other factors, such as the mother’s health-seeking behaviour and education were major factors associated with the children’s vaccination status. Given the results of this study, it is strongly recommended that the vaccination coverage is increased by improving access to antenatal care and education for the parents.

## Introduction

The public health community agrees that a full course of vaccinations against communicable diseases such as measles, polio and all the others included in the Expanded Programme of Immunization (EPI) is one of the most effective and cost-effective public health interventions to reduce morbidity and mortality of children under 5 years of age. However, vaccination coverage in children varies greatly worldwide, both between and within countries, with low and middle-income countries having lower coverage.[1] The World Health Organisation (WHO) and the United Nations Children’s Fund (UNICEF) jointly estimated that measles vaccination in the African Region reached 74% in 2013, while the estimates for the same year were 78% for South-East Asia and Eastern Mediterranean Regions, 90% for the Americas, 95% for European and 97% for Western Pacific Regions.[2] In general, African countries appear to have a lower vaccination coverage than other regions of the world: for example, the African countries with the highest coverage have the same coverage levels as the countries with the lowest coverage in the WHO Region of Americas.[1] Vaccination coverage should ideally reach levels that allow herd immunity to work in order to keep the propagation of the disease to a level that is no longer a public health problem when eradication or elimination are not achievable.

### Literature Review

In spite of the importance of the issue, differences in coverage between and within countries have been marginally explored and mostly from a descriptive rather than analytical perspective. Evidence so far shows that vaccination coverage is influenced by different factors. These differences mainly involve programmatic issues such as availability of health personnel [3]; perception of children’s parents or guardians toward vaccines [4,5]; global policies, laws and financial issues. [6,7,8,9]

However, research at the individual level on factors that may be associated with the vaccination status of children is limited. In order to explore such individual or household factors associated with vaccination status, a PUBMED literature search was performed in October 2013 and again in August 2014 to account for possible new studies published on this topic. In particular, the literature search used key words that focused on the association in the African continent between the marital status of the mother/maternal living arrangements and the vaccination status of their children, aiming at identifying studies carried out in the last 10 years. Few studies have been carried out in Africa and these have been mostly inconclusive in uncovering factors associated with the vaccination status of children. A study conducted in three districts in Niger in 2005 [10] attempted to assess whether the drop-out of vaccination in children of up to 11 months was associated with age, education, occupation and marital status of the mother and the only significant association was found in one district with education of the mother. However, the sample size of this study was small, (415 children in total). A similar study was carried out in one rural health district of Senegal [11] with a sample size of 562 children, (aged 12–23 months), surveyed. This study found that the major causes for not being fully vaccinated were linked to poor knowledge of the effects of vaccination and economic factors of the parents while no association was found concerning education of the parents and marital status of the mother. Another cross-sectional study carried out in rural Mozambique [12] with a sample of 668 children under 2 found strong association between incomplete vaccination status and education of the mother, knowledge about vaccination, place of delivery and religious beliefs but no association with the age and marital status of the mother. An analysis of the Demographic Health Surveys (DHS) 2011 from Ethiopia [13] explored inequalities in childhood immunization, and revealed that the wealth index was strongly and linearly associated with vaccination status but no other factors were explored. Another study, carried out in a peri-urban area in Abidjan, Ivory Coast [14], among 669 children aged 12–59 months, revealed that education, knowledge of the vaccination calendar and marital status of the mother were all strongly associated with the full vaccination coverage of children. Similarly, another cross-sectional study carried out in Kenya [15], also reported that children living in a household with only one parent are less likely to be fully vaccinated.

The above-mentioned studies were inconclusive and conflicting in identifying which factors are associated with full child-vaccination coverage. In most instances, these studies were done in small and precise areas of a given country, they had small sample sizes and therefore were not generalizable for the whole country. With only two exceptions, [14,15] these studies could not find an association between the marital status of the mother and the vaccination status of their children.

### Study rationale

In 2012, under-5 mortality rate in Zimbabwe was 90/1,000 live-births, (about double of the global average), and measles accounted for about 1% of the total mortality rate for the same age group.[16,17]. Therefore, investigating potential risk factors associated with incomplete vaccination of children is essential to propose and implement public health interventions aiming at reducing childhood mortality and morbidity.

Due to the financial crisis that affected Zimbabwe between 2007–2010, the parents in many families became separated as one of the parents (usually the father) had to leave the household to search a job in other countries of the region. The underlying hypothesis of this study is that mothers without partner’s support may be busier in accomplishing daily tasks, therefore having less time to seek preventive care for their children, including vaccination.

This study will assess whether maternal living arrangements are associated with vaccination coverage of children in Zimbabwe. It aims at assessing whether children aged 12–23 months whose mothers are without a current partner (whether single, divorced, widower, or simply not cohabiting with the father) have a different vaccination status compared to children living with both parents. Moreover, it will explore other variables for the same outcome, these being potential confounders, independent or interactive factors, and propose public health interventions on the basis of the results. By using the DHS dataset of Zimbabwe for 2010–2011, which has a large sample size, the results obtained are more likely to be representative of the whole country.

## Materials and Methods

### Ethics

The Zimbabwe National Statistics Agency (ZIMSTAT) conducted the DHS survey and obtained written informed consent from the participants (or parents/guardians when children were involved) to participate in the survey. The MEASURE DHS Data Archive/ICF International granted permission to download the dataset from the internet DHS website following the formal request of the undersigned. The Ethical Committee of the London School of Hygiene & Tropical Medicine (LSHTM) approved, on the 6^th^ June 2014, the present study following the submission of the CARE form (LSHTM MSc Ethics Ref: 7440).

### Materials

#### Data

The last Demographic Health Surveys (DHS) for Zimbabwe was carried out in 2010–2011 (DHS-VI) [18]. The current study uses the Children Records (KR) file.

This study included, as main study population, Zimbabwean children of both sexes, aged 12–23 months and their mothers. Only one child per household was randomly selected and included in the analysis so as to avoid household-level clustering. The Children Records file also included information on the mother (or primary care giver) and the household. This stratified DHS survey was conducted with 2-stage cluster design where enumeration areas were the sampling units for the first stage, (Primary Sampling Units – PSU), and households were the units for the second stage. The DHS survey reached all the provinces of Zimbabwe, rural and urban areas, with a response rate of 93%.[18] The initial sample size of the dataset included more than 9,000 household and 5,000 children.

#### Design

This study consists of an analysis of DHS-VI data from Zimbabwe. The main outcome of interest in this study was whether a child aged 12–23 months was or was not fully vaccinated at the time of the survey. A child was considered “fully vaccinated”, (yes or no), if it had received at least the following vaccine doses in accordance with the vaccination calendar of the Zimbabwean Ministry of Health and Child Welfare (MoHCW): one dose of Bacillus Calmette–Guérin (BCG), 3 doses of Polio, 1 dose of Measles, and 3 doses of DPT or Pentavalent, which, in addition to Diphtheria-Pertussis-Tetanus (DPT), also includes Hepatitis B and Haemoinfluenza B.[18] DPT or Pentavalent are included because, during the survey, Zimbabwe experienced the co-existence of both vaccines since the DPT was phased out and Pentavalent was introduced. The main exposure variable was the maternal living arrangements with three levels of exposure. These are: 1) whether the mother of the child was married, (or with a partner), and cohabiting at the time of the survey (unexposed); 2) whether the mother was married, (or with a partner), and he was living elsewhere, (first level of exposure), and; 3) whether the mother was unmarried, divorced/separated or widowed, (second level of exposure).

Other variables were considered in the analysis as potential independent risk factors for not being fully vaccinated, potential confounders for the association between main exposure and outcome, or effect modifiers. These were: age of the mother, (in 5-year age groups), and child, (in months), living in either urban or rural areas, education level, and occupation of the mother and husband/partner, wealth index of the household, number of living children in the household, variables of health-seeking behaviour and access to health care, sex of the child, whether the child was living with the mother or somewhere else and language of the questionnaire, (English, Shona or Ndebele), as the proxy indicator of ethnic group, since there was no question of asking for the ethnic group. In fact, it is possible that both Shona and Ndebele respondents may have preferred to answer the questionnaire in English, particularly if they were highly educated. The wealth index had 5 levels of exposure from the “poorest” to the “richest”, and was defined through the use of given indicators that divide the population in quintiles of wealth.[18]

**Fig 1:**
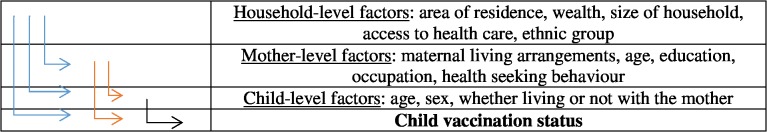
Conceptual hierarchical framework of risk factors for not being fully vaccinated in Zimbabwe. Arrows indicate potential relationships/influence between factors and final outcome.

A hierarchical/conceptual framework was developed in order to organise these variables into different levels [19]: child, maternal and household-related (Fig 1).

#### Tools

All the above-mentioned variables were obtained, (or constructed), from the DHS-VI standard questionnaire (S1 Questionnaire) that was administered in Zimbabwe in 2010–2011.[20]

STATA version 11 was used for the data management and analysis.

### Methods

#### Selection and construction of dataset variables

Variables that were not applicable to the survey, as well as variables that were not relevant to the analysis were removed from the dataset. The variable for the main outcome of interest was constructed from the relevant variables indicating whether a child had received the full course of each antigen by combining them together, resulting in a dichotomous outcome variable. Therefore, children were classified as “fully vaccinated” if they had received the full course of all vaccines, or “not fully vaccinated” if even one vaccine course had not been completed.

The variable for the main exposure of interest was constructed from two existing variables in the DHS dataset: one reporting the current marital status of the mother, (6 levels), and one reporting whether married mothers were currently living with the partner/husband or whether he was living away, (but not separated/divorced). These two variables were combined to obtain the maternal living arrangements; the resulting variable had three levels of exposure: whether the mother was married/partner and if she was living with him, (unexposed); whether the mother was married/partner, but the partner/husband lived elsewhere, (first level of exposure); or whether the mother was divorced/separated, widowed or a single mother without any further distinction, (second level of exposure). Mother’s occupation, which had 8 different levels was re-categorised into 3 levels: unemployed, unskilled employed/domestic/agricultural, or skilled employed.

Different variables concerning the mother were considered as a proxy for health-seeking behaviour. The place where the mother delivered had 13 levels, ranging from home to different types of hospitals or other health facilities and it was recoded into a dichotomous variable: whether the mother delivered in a health facility or at home. Tetanus toxoid injection was considered as a proxy of health-seeking behaviour before birth, (whether the mother had received at least one dose or no doses), while the mother’s health check-up after delivery (yes-no) was considered as a proxy of mother’s health-seeking behaviour for after the birth.

Only children aged 12–23 months at the time of the survey were included in the dataset.

In order to account for weight and survey design, a “weight” variable was created from the women’s individual sample weight of the existing dataset and the data were set for survey design for the PSU. There was no need to account for household clustering since only one child was kept for the analysis.

Age of children, which was on continuous scale, was transformed into a dichotomous variable, dividing the group at the median of 17 months, resulting into two levels (12–17 and 18–23 months). The variable reporting the number of children living within the same household was also on a continuous scale and was also dichotomised into two levels (up to 3 children, or more than 3 children in the same household).

Similarly with the mother’s occupation, husband’s/partner’s occupation was recoded in four levels: unemployed, unskilled employed, skilled employed and unknown. Education of both mother and husband were recoded in three levels, (no education/primary, secondary/higher, or unknown).

All the other variables of interest were kept as originally set in the initial database.

#### Statistical analysis

The dataset was explored by means of tabulations of each variable of interest. Three tables were constructed that reported unweighted frequencies of participants and weighted percentages, with 95% Confidence Interval (95% CI), for each level of the variable: children-related, mothers-related and one the household-related variables (Tables 1, 2, and 3). The weighted percentage of fully vaccinated children was calculated for each level of all the variables, along with the 95%CI and P-value from Pearson Test, and presented in the same 3 tables.

**Table 1:** Percentage distribution of children aged 12–23 months and percentage fully vaccinated by selected background characteristics.

Variable	N (unweighted)	Weighted % (95%CI)	% of fully vaccinated children (95%CI)*	p-value§
Gender:				0.54
**Male**	514	49.9 (46.5; 53.3)	64.7 (59.5; 69.6)	
**Female**	517	50.1 (46.7; 53.5)	66.8 (61.4; 71.9)	
Age group (in months):				0.08
**12–17**	602	58.8 (55.1; 62.4)	63.2 (58.5; 67.8)	
**18–23**	429	41.2 (37.6; 44.9)	69.3 (63.3; 74.8)	
Child lives with:				0.01
**Mother**	985	95.4 (93.7; 96.6)	66.7 (62.6; 70.5)	
**Elsewhere**	46	4.6 (3.4; 6.2)	46.6 (31.4 ; 62.4)	
**Completed vaccination of the child**				
BCG:			—	
**Yes**	913	87.0 (83.9; 89.6)		
**No**	118	13.0 (10.4; 16.0)		
Polio:			—	
**Yes**	777	73.9 (70.0; 77.5)		
**No**	254	26.1 (22.5; 30.0)		
DPT or Pentavalent:¤			—	
**Yes**	780	73.5 (69.5; 77.1)		
**No**	251	26.5 (22.9; 30.5)		
Measles:			—	
**Yes**	831	79.2 (75.9; 82.2)		
**No**	200	20.8 (17.8; 24.1)		
Fully vaccinated:†			—	
**Yes**	690	65.8 (61.8; 69.5)		
**No**	341	34.2 (30.5; 38.2)		

This is the Table 1 legend.

Frequencies are unweighted (N = 1031, unless otherwise indicated), percentages and their 95%CI are weighted. The table includes vaccination status for each antigen.

*weighted percentages accounting survey design

§p-values from Pearson Test

¤Complete course of vaccination for either DPT or Pentavalent were considered since the country was facing a transition (phasing out DPT while introducing Pentavalent) at the time of the DHS survey.

†A “Fully vaccinated” child has received at least the following doses: 1 dose of BCG, 3 doses of Polio, 3 doses of either DPT or Pentavalent, 1 dose of Measles.

**Table 2:** Percentage distribution of mother-level variables and percentages of fully vaccinated children for each variable’s level.

Variable	N (unweighted)	weighted % (95%CI)	% of fully vaccinated children (95%CI)*	p-value§
**Age group (in years):**				0.7
15–19	108	9.8 (8.0; 12.1)	60.9 (50.6; 70.4)	
20–24	341	33.9 (30.8; 37.2)	65.8 (60.1; 71.1)	
25–29	291	28.0 (25.1; 31.0)	68.3 (61.9; 74.0)	
30–34	159	15.5 (13.1; 18.2)	66.1 (57.2; 74.1)	
35+	132	12.8 (10.6; 15.0)	63.3 (53.6; 72.1)	
**Highest educational level:**				<0.0001
No education/Primary	320	30.1 (26.8; 33.4)	55.2 (47.8; 62.4)	
Secondary or higher	711	69.9 (66.6; 73.2)	70.3 (66.4; 73.9)	
**Occupation:**				0.8
Unemployed	634	58.4 (54.7; 62.0)	66.1 (61.2; 70.7)	
Unskilled employed	226	23.5 (20.4; 26.8)	66.6 (59.5; 72.9)	
Skilled employed	171	18.1 (15.4; 21.1)	63.4 (54.2; 71.7)	
**Place of delivery:**				<0.0001
Any health facility	663	63.1 (59.1; 67.0)	71.6 (67.6; 75.3)	
At home	368	36.9 (33.0; 40.9)	55.7 (48.6; 62.6)	
**Mother health check-up after delivery**				0.0009
No	492	48.3 (44.3; 52.3)	59.3 (53.0; 65.4)	
Yes	539	51.7 (47.6; 55.7)	71.8 (67.4; 75.7)	
**Mother received at least 1 dose of tetanus vaccination before delivery:**				<0.0001
Yes	822	78.2 (75.0; 81.1)	70.3 (66.5; 73.8)	
No	209	21.8 (18.9; 25.0)	49.5 (41.4; 57.7)	
**Mother has health insurance:**				0.1
No	975	94.4 (92.6; 95.9)	65.1 (61.0; 69.0)	
Yes	56	5.5 (4.1; 7.4)	76.2 (62.5; 86.0)	
Maternal living arrangements:				0.9
Married/partner cohabitating	661	65.7 (62.4; 68.9)	65.7 (60.8; 70.3)	
Married, husband/partner lives elsewhere	211	20.3 (17.6; 23.2)	64.7 (57.4; 71.3)	
Not married (single/divorced/separated/widowed)	159	14.0 (11.7; 16.7)	67.5 (58.8; 75.1)	

This is Table 2 legend

Frequencies are unweighted (N = 1031, unless otherwise indicated), percentages and their 95%CI are weighted.

*weighted percentages accounting survey design

§p-values from Pearson Test.

**Table 3:** Percentage distribution of household-level variables and percentages of fully vaccinated children for each variable’s level.

Variable	N (unweighted)	weighted % (95%CI)	% of fully vaccinated children (95%CI)*	p-value§
**Area of residence:**				0.2
Urban	291	29.0 (26.2; 31.9)	69.8 (61.6; 76.9)	
Rural	740	71.0 (68.1; 73.8)	64.1 (59.5; 68.5)	
**Wealth index:**				0.01
Poorest	240	21.8 (18.7; 25.2)	57.3 (49.5; 64.7)	
Poorer	202	20.3 (17.8; 23.0)	64.4 (55.9; 72.0)	
Middle	203	20.8 (17.9; 24.1)	63.1 (55.1; 70.4)	
Richer	222	20.8 (17.9; 24.0)	73.5 (66.8; 79.3)	
Richest	164	16.3 (13.6; 19.6	72.2 (64.1; 79.1)	
**Total number of children in the household:**				0.0015
1–3	819	79.0 (76.1; 81.6)	68.4 (64.5; 72.1)	
4+	212	21.0 (18.3; 23.9)	55.6 (47.3; 63.6)	
**Distance to health facility:**				0.2
“Big problem”	447	41.9 (37.6; 46.2)	63.0 (56.8; 68.8)	
“Not a big problem”	584	58.1 (53.8; 62.4)	67.7 (63.1; 72.1)	
**Husband/partner education level**	(N = 968)			0.4
No education/Primary	224	21.5 (17.6; 23.5)	66.9 (58.0; 74.8)	
Secondary or Higher	732	78.3 (75.3; 81.2)	65.7 (61.2; 70.0)	
Don’t know	12	1.2 (0.4; 2.0)	45.3 (22.1; 70.8)	
**Husband/partner occupation:**	(N = 968)			0.39
Unemployed	139	14.4 (11.8; 17.5)	61.8 (49.7; 72.7)	
Unskilled employed	375	38.6 (34.9; 42.3)	63.2 (57.0; 69.0)	
Skilled employed	410	42.7 (39.0; 46.5)	68.7 (63.4; 73.5)	
Don’t know	44	4.3 (3.2; 5.9)	71.7 (54.9; 84.1)	
**Ethnic group/language:**				0.01
Shona	713	76.6 (73.3; 79.6)	63.1 (58.2; 67.7)	
Ndebele	216	12.8 (11.1; 14.7)	75.0 (68.0; 80.8)	
English	102	10.6 (8.3; 13.4)	74.1 (63.5; 82.5)	

This is Table 3 legend

Frequencies are unweighted (N = 1031, unless otherwise indicated), percentages and their 95%CI are weighted.

*weighted percentages accounting survey design

§p-values from Pearson Test.

Univariate analysis was performed through survey-accounted logistic regression by calculating the crude Odds Ratios (OR), along with the 95%CI, for a child not being fully vaccinated for all the variables in turn. P-values from the crude analysis were calculated from the Wald Test. Results were summarised into 3 different tables, following the same logic as above: child-related variables (Table 4), mother-related variables (Table 5), and household-related variables (Table 6). Similarly, three multivariable logistic regression models were constructed for each group of variables, which included all the relevant variables of the group, and presented in the same tables. In both univariate and multivariable models, OR for categorical variables were also calculated in ordered scale to assess potential linear trends, which were then reported if relevant.

**Table 4:** Crude and adjusted association between different child-related variables and the child not being fully vaccinated. Measure of effect expressed as Odds Ratio (OR) from survey-accounted logistic regression.

Variable	Crude OR		Adjusted OR*	
	OR (95%CI)	p-value§	OR (95%CI)	p-value§
**Child gender:**				
Male	(ref)		(ref)	
Female	0.91 (0.67;1.23)	0.541	0.90 (0.67; 1.23)	0.516
**Child age group (in months):**				
12–17	(ref)		(ref)	
18–23	0.76 (0.56; 1.04)	0.083	0.69 (0.50; 0.96)	0.028
**Child lives with:**				
Mother	(ref)		(ref)	
Elsewhere	1.23 (1.04; 1.45)	0.014	1.29 (1.08; 1.53)	0.005

This is Table 4 legend

*OR adjusted for the other variables in the table

§p-value from Wald Test.

**Table 5:** Crude and adjusted association between different mother-related variables and the child not being fully vaccinated. Measure of effect expressed as Odds Ratio (OR) from survey-accounted logistic regression.

Variable	Crude OR		Adjusted OR*	
	OR (95%CI)	p-value§	OR (95%CI)	p-value§
**Age group (in years):**				
15–19	(ref)		(ref)	
20–24	0.81 (0.51; 1.29)	0.376	1.00 (0.63; 1.59)	0.990
25–29	0.72 (0.45; 1.18)	0.192	0.85 (0.52; 1.39)	0.515
30–34	0.80 (0.45; 1.42)	0.441	0.84 (0.47; 1.48)	0.540
35+	0.90 (0.53; 1.54)	0.706	1.00 (0.56; 1.77)	1.000
Linear trend	0.99 (0.87; 1.12)	0.850	0.98 (0.86; 1.11)	0.694
**Highest educational level:**				
No education/primary	(ref)		(ref)	
Secondary or higher	0.52 (0.38; 0.71)	<0.0001	0.63 (0.45; 0.88)	0.007
**Occupation:**				
Unemployed	0.89 (0.60; 1.32)	0.555	0.77 (0.51; 1.14)	0.195
Unskilled employed	0.87 (0.53; 1.43)	0.582	0.69 (0.42; 1.13)	0.142
Skilled employed	(ref)		(ref)	
**Place of delivery:**				
Any health facility	(ref)		(ref)	
At home	2.00 (1.44; 2.79)	<0.0001	1.44 (1.01; 2.05)	0.041
**Mother health check-up after delivery:**				
No	1.74 (1.26; 2.41)	0.001	1.41 (1.03; 1.93)	0.041
Yes	(ref)		(ref)	
**Mother received at least 1 dose of tetanus vaccination before delivery:**				
Yes	(ref)		(ref)	
No	2.41 (1.73; 3.39)	<0.0001	1.96 (1.39; 2.75)	<0.0001
**Mother has health insurance:**				
No	1.72 (0.88; 3.36)	0.114	1.24 (0.63; 2.44)	0.536
Yes	(ref)		(ref)	
**Maternal living arrangements:**				
Married/partner cohabitating	(ref)		(ref)	
Married, husband/partner lives elsewhere	1.05 (0.74; 1.48)	0.795	1.16 (0.81; 1.66)	0.411
Not married (single/divorced/separated/widowed)	0.92 (0.61; 1.39)	0.699	0.91 (0.59; 1.41)	0.666

This is Table 5 legend

*OR adjusted for the other variables in the table

§p-value from Wald Test.

**Table 6:** Crude and adjusted association between different household-related variables and the child not being fully vaccinated. Measure of effect expressed as Odds Ratio (OR) from survey-accounted logistic regression.

Variable	Crude OR		Adjusted OR*	
	OR (95%CI)	p-value§	OR (95%CI)	p-value§
**Area of residence:**				
Urban	(ref)		(ref)	
Rural	1.30 (0.86; 1.96)	0.219	0.70 (0.38; 1.27)	0.239
**Wealth index:**				
Poorest	(ref)			
Poorer	0.74 (0.50; 1.10)	0.140		
Middle	0.79 (0.49; 1.25)	0.311		
Richer	0.48 (0.31; 0.76)	0.002		
Richest	0.52 (0.32; 0.84)	0.008		
Linear trend	0.84 (0.75; 0.94)	0.002	0.81 (0.70; 0.95)	0.012
**Total number of children in the household:**				
1–3	(ref)		(ref)	
4+	1.73 (1.23; 2.43)	0.002	1.61 (1.13; 2.30)	0.008
**Distance to health facility:**				
“Big problem”	(ref)		(ref)	
“Not a big problem”	0.81 (0.59; 1.11)	0.187	0.94 (0.66; 1.35)	0.746
**Husband/partner education level:**				
No education/primary	(ref)		(ref)	
Secondary or higher	1.06 (0.70; 1.59)	0.795	1.31 (0.87; 1.98)	0.189
Don’t know	2.44 (0.76; 7.83)	0.133	2.89 (0.94; 8.91)	0.064
**Husband/partner occupation:**				
Unemployed	1.35 (0.78; 2.34)	0.164	1.14 (0.69; 1.87)	0.615
Unskilled employed	1.28 (0.90; 1.80)	0.277	1.12 (0.78; 1.60)	0.550
Skilled employed	(ref)		(ref)	
Don’t know	0.85 (0.41; 1.83)	0.703	1.03 (0.47; 2.27)	0.943
**Ethnic group/language:**				
Shona	(ref)		(ref)	
Ndebele	0.57 (0.38; 0.86)	0.007	0.51 (0.32; 0.82)	0.005
English	0.60 (0.35; 1.02)	0.060	0.58 (0.32; 1.04)	0.067

This is Table 6 legend

*OR adjusted for the other variables in the table

§p-value from Wald Test.

Finally, a multivariable survey logistic regression model was constructed using a forward stepwise approach. The main exposure of interest was always kept in the model, and all the other variables, (independently from whether they were child, mother or household-related), were added one by one to the model, commencing with those variables that appeared to be statistically associated with the outcome from the previous step of the analysis. A variable was kept in the model if it showed being an important confounder, (either positive or negative), between the main exposure and the outcome, (assessed by visual inspection of the change in OR of +/-10%), or an independent risk factor for a child not having been fully vaccinated, (assessed by strength of association, 95%CI, and P-value from the Wald Test), even if did not confound the association between maternal living arrangements and the child not having been fully vaccinated. The main exposure of interest, (maternal living arrangements), was always kept in the model independently of its association with the outcome. The possible interactions between the main exposure and other variables on the outcome were assessed by including an interaction term between the main exposure and a given variable and then assessed by the P-value from the Wald Test for interaction. In this last model, ordered categorical variables were only included so as to assess their linear trend whenever this was statistically significant, this was done so as to simplify the model when appropriate.

Statistical significance was considered at p = <0.05.

## Results

### Sample size and description of the distribution of variables

After removing the observations of children younger than 12 months and older than 23 months, and possible twins or other children in the same age group within the same household, the total sample size was 1,031, (unweighted), children, living in the same number of households. All the children were alive at the time of the survey. Account for weight and survey design reduced the sample size to 1,008 observations. Unweighted frequencies together with weighted percentages are presented here. This is in line with the DHS report for Zimbabwe.[18] No missing values were observed in the dataset.

The gender of children aged 12–23 months was found to be well balanced, with 514 boys and 517 girls, representing 49.9% and 50.1% of the study population, respectively. The mean age of children was 16.9 months (16.8 for boys and 17.0 for girls). Most of the children (58.8%) were aged 12–17 months, while the remaining 41.2% were 18–23 months. The majority of children, (95.4%), lived with their mothers at the time of the survey while the rest were living elsewhere (the survey did not specify further). See Table 1.

The mean age of the mothers was 26.6 years, and the most represented age groups were 20–24 years (33.9%) and 25–29 years (28.0%). About 70% of the mothers reported to have had at least a secondary school level of education, while only about 1% reported to have had no education at all. In spite of this, more than 58% were unemployed and only 18.1% were in skilled employment. 63% of children were delivered in a health facility while the remaining 37% were delivered at home; 51.7% reported having received a health check-up after delivery, and 78.2% reported or could prove they had received at least 1 dose of tetanus toxoid vaccination during pregnancy.

With regard to maternal living arrangements, (main exposure of interest), 661 mothers (65.7%) were married or with a partner and cohabiting; 20.3% (n = 211) reported to be married or with a partner but living separately from the partner, while 159 mothers (14.0%) were reported as being either single mothers, divorced, separated or widowed (Table 2).

Most of the surveyed households were living in rural areas (71.0%), and the most represented ethnic group was the Shona (76.6%), followed by the Ndebele (12.8%) and English (10.6%). The wealth index grouping was done in quintiles, therefore each level should contain about 20% of the observations. While this is true for the 3 middle groups (“poorer”, “middle”, “richer”), the “poorest” are slightly more represented (21.8%) and the “richest” less represented (16.3%). The mean number of children living in the same household was 2.4, with most of the households (79%) reported having 3 or fewer children and only 21% reported 4 or more children. With regard to access to a health facility in terms of physical distance, 41.9% of the mothers reported this to be a “big problem”, while the remaining 58.1% reported this as “not a big problem”. Married or cohabiting mothers also gave information regarding their husband’s/partner’s education and employment (n = 968). About 78% of the husbands/partners achieved at least a secondary level of education, and over 80% were employed at the time of the survey. However, 1.2% and 4.3% of mothers were not able to report on their husband’s/partner’s education and employment status, respectively, by answering “I don’t know” (Table 3).

In terms of outcome variables, 65.8% of children were “fully vaccinated”, meaning that they had received at least 1 dose of BCG (Bacillus Calmette–Guérin) against Tuberculosis, 3 doses of either DPT (Diphtheria-Pertussis-Tetanus) or Pentavalent (which in addition to DPT also includes antigens for Hepatitis B and Haemo-influenza B) and Polio, and one dose of Measles. The percentage who received specific individual vaccination varied. Most children received a BCG vaccination (87.0%), given in a single dose shortly after birth; 79.2% were vaccinated against measles, given in a single dose at around 9 months of age but fewer children received the other vaccines (DPT or Pentavalent, and Polio). These required a minimum of 3 doses to complete the full course. The complete coverage for DPT/Pentavalent was 73.5%, while it was 73.4% for polio (Table 1).

### Results from survey cross-tabulation: percentages of fully vaccinated children across different factors

Children living with their mothers were more likely to be fully vaccinated: 66.7% as against 46.6% of the children living elsewhere. The difference of being fully vaccinated between the 2 age groups of children was marginal, 69.4% of children aged 18–23 months and 63.2% of the children aged 12–17 months attained full vaccination. Vaccination status did not vary by sex of child (Table 1).

With regard to mother-related variables, maternal living arrangement levels of exposure did not show any significant difference in outcome (p = 0.9), and the percentage of children fully vaccinated was very similar across these 3 levels of exposure, ranging from 64.7% for married/partner mothers where the husband/partner lives elsewhere, to 67.5% for not married mothers. However, other mother-related variables showed important differences in outcome, and these mostly involved health-seeking behaviour during pregnancy or just after delivery: 71.6% of children of mothers who delivered in a health facility (p<0.0001), 71.8% of children whose mothers had a health check-up after delivery (p = 0.0009), and 70.3% whose mothers received at least one dose of tetanus toxoid vaccination (p<0.0001) were fully vaccinated. Mother’s education also showed some differences in outcome distribution (p<0.0001), with children of mothers with secondary or higher education being more likely to be fully vaccinated (70.3%), compared to children whose mothers had lower levels of or no education (55.2%). Mother’s age was not associated with vaccination status (Table 2).

The area or residence (urban or rural), the perception of the distance to the health facility, the husband’s/partner’s education and employment showed no significant differences in outcome distribution. However, distribution of fully vaccinated children varied across the different levels of the wealth index, with 57.3% of the “poorest” households, and 73.5% and 72.2% of the “richer” and “richest” households, respectively, having had their children fully vaccinated (p = 0.01). The total number of children in a household also showed varying outcome distribution, with 68.4% of children living in households having up to 3 children having been fully vaccinated, against 55.6% of children living in households with 4 or more children (p = 0.001). See Table 3.

### Univariate and multivariable results within each category of variables

Univariate survey logistic regression and results from the multivariable models, which include only variables of the same group (child-, mother- or household-related variables), are summarised below. Although cross-tabulation reported the percentage of children fully vaccinated, the regression analysis reports the OR for “not fully vaccinated” children.

Child-related variables (Table 4) – In the regression analysis, children not living with their mothers were found to have 23% higher odds (crude OR = 1.23, 95%CI 1.04 to 1.45, p = 0.014) of not being fully vaccinated compared to children living with their mothers. Once adjusted for sex and age group of the child, this association became slightly stronger (adjusted OR = 1.29, 95%CI 1.08 to 1.53, p = 0.005). Children aged 12–17 months were less likely to be fully vaccinated than those aged 18–23 months, and this association was significant in both the crude and adjusted models (adjusted OR = 0.69, 95%CI = 0.50 to 0.96, p = 0.03). Sex of the child was not associated with vaccination status.

Mother-related variables (Table 5) – Maternal living arrangements were not found to be associated with vaccination status, and this was true in both the crude and adjusted analysis. It is worth noting that children whose mothers were not cohabiting were slightly more likely to be unvaccinated compared to those living with cohabiting mothers (adjusted OR = 1.16, 95% CI 0.81 to 1.66, p = 0.4); unmarried mothers had 9% lower odds of having their child not fully vaccinated compared to cohabiting married women (OR = 0.91, 95% CI 0.59 to 1.41, p = 0.67). Other maternal factors associated with a child not reaching full vaccination were mostly those linked to health seeking behaviour: in the crude analysis, not having received at least one dose of tetanus vaccine was found to be associated with a 2.4 increase in the odds of having the child not fully vaccinated compared to women who had received at least one dose of tetanus vaccine (95%CI 1.7 to 3.4, p<0.0001). Although reduced, the adjusted OR remained strongly significant (adjusted OR = 1.96, 95%CI 1.39 to 2.75, p<0.0001). The place of delivery (either at home or in a health facility) and mother check-up after delivery were both strongly associated with having their child not fully vaccinated in the crude and adjusted analyses (adjusted OR = 1.44, 95%CI 1.01 to 2.05, p = 0–04 and OR = 1.41, 95%CI 1.03 to 1.93, p = 0.04, respectively). The mother’s educational level was also strongly associated with having the child not fully vaccinated; compared to children of mothers with no or only primary education, those with a mother with at least secondary education were less likely to be unvaccinated (adjusted OR = 0.63, 95% CI 0.45 to 0.88, p = 0.007). Mother’s age, occupation and having health insurance were not associated with the outcome in both crude and adjusted models, even if the crude OR varied compared to the adjusted OR.

Household-related variables (Table 6) – Wealth index was linearly and inversely associated with having a child not fully vaccinated. In the adjusted analysis, each increase in wealth index quintile (from the “poorest” to the “richest”) decreases the odds by 19% (OR = 0.81, 95%CI 0.70 to 0.95, p = 0.012) of a child being unvaccinated. Husband’s/partner’s education was not associated with having a child not fully vaccinated but when a mother reported being unaware of husband’s/partner’s education, compared to no/primary education, a marginal association was found (adjusted OR = 2.89, 95%CI 0.94 to 8.91, p = 0.06); this association, however, did not appear in the crude analysis (p = 0.13). However, only 12 mothers could not report on their husband’s/partner’s education. A relevant factor associated with not having a child fully vaccinated was the number of children living in the household: compared to households with up to 3 children, those with 4 or more had 61% higher adjusted odds (95%CI 1.13 to 2.30, P = 0.008); crude OR were similar. The language of the respondents (as proxy for ethnic group) was also associated with the outcome. Compared to Shona speakers, Ndebele and English speakers were less likely to have their children unvaccinated (for Ndebele adjusted OR = 0.51, 95% CI 0.32 to 0.82, p = 0.005; for English OR = 0.58, 95%CI 0.32 to 1.04, p = 0.067). The other household-related variables (area of residence, perceived distance to the health facility, and husband’s/partner’s occupation) were not found to be associated with the outcome in both crude and adjusted analysis.

### Results from the multivariable survey logistic regression with different group variables together – final regression model (Table 7)

Maternal living arrangements were not associated with vaccination status in the last multivariable model. Compared to married cohabiting mothers, married mothers whose husband/partner lives elsewhere had 3% higher odds of having their child not fully vaccinated (95%CI 0.71 to 1.48, p = 0.88); for unmarried mothers, the OR = 0.75 (95%CI 0.45 to 1.27, p = 0.28). The mother’s health-seeking behaviour during pregnancy but before delivery, (expressed as having received or not at least one tetanus vaccine dose during pregnancy), was the strongest factor associated with not having a child fully vaccinated (adjusted OR = 2.09, 95%CI 1.46 to 2.99, p<0.0001). However, the place of delivery remained only marginally associated with the outcome in the final adjusted model (adjusted OR = 1.44, 95%CI 1.00 to 2.07, p = 0.05). Similarly, the mother’s health check-up strength of association decreased in the final analysis (adjusted OR = 1.34, 95%CI 0.94 to 1.89, p = 0.1). The mother’s education remained significantly associated with the outcome although the adjusted OR was slightly less significant compared to the crude OR (adjusted OR = 0.65, 95%CI 0.45 to 0.92, p = 0.016). The husband’s/partner’s education had a similar strength of association on vaccination status as mother’s, but simply in the opposite direction (adjusted OR = 1.56, 95%CI 1.00 to 2.43, p = 0.048): increase in husband’s/partner’s education increased the OR by about 56% whereas an increase in mother’s education decreased the OR by about 35%. Again, the mother’s report of being unaware of husband’s/partner’s education, compared to no/primary education, a marginal association was found (adjusted OR = 2.63, 95%CI 0.84 to 8.22, p = 0.096). In the final model, other household-related factors showed varying strengths of association: linear trend of wealth index became less and less significantly associated with the outcome as new variables were added in the model and finally resulted to be virtually null (adjusted OR = 0.95, 95%CI 0.83 to 1.08, p = 0.4), while the language of the questionnaire and total number of children in the household remained significant: adjusted OR = 0.62 and 0.66 for Ndebele and English vs Shona (95% CI 0.39 to 0.99, p = 0.05, and 95%CI 0.37 to 1.15, p = 0.14), respectively, and adjusted OR = 1.52 for household with 4 or more children compared with those with up to 3 children (95% CI 1.05 to 2.20, p = 0.03). With regard to child-related factors, children living away from their mothers showed having 50% higher odds of not being fully vaccinated compared to children living with their mothers (95%CI 1.25 to 1.79, p<0.0001). Children aged 18–23 months appeared to be more likely to be fully vaccinated than those aged 12–17 months also in the adjusted final model and this association was stronger than in the crude analysis (adjusted OR = 0.68, 95%CI 0.49 to 0.94, p = 0.02); this may indicate a sort of delay in vaccinating children, who actually may become fully vaccinated only later.

**Table 7:** Crude (as per Tables 4, 5, and 6) and adjusted OR between different variables and the child not being fully vaccinated. OR from survey-accounted logistic regression.

Variable	Crude OR		Adjusted OR*	
	OR (95%CI)	p-value§	OR (95%CI)	p-value§
**Maternal living arrangements:**				
Married/partner cohabiting	(ref)		(ref)	
Married/partner, husband/partner lives elsewhere	1.05 (0.74; 1.48)	0.795	1.03 (0.71; 1.48)	0.882
Not married (single/divorced/separated/widowed)	0.92 (0.61; 1.39)	0.699	0.75 (0.45; 1.27)	0.280
**Mother received at least 1 dose of tetanus vaccination before delivery:**				
Yes	(ref)		(ref)	
No	2.41 (1.73; 3.39)	<0.0001	2.09 (1.46; 2.99)	<0.0001
**Child lives with:**				
Mother	(ref)		(ref)	
Elsewhere	1.23 (1.04; 1.45)	0.014	1.50 (1.25; 1.79)	<0.0001
**Mother’s highest educational level:**				
No education/primary	(ref)		(ref)	
Secondary or higher	0.52 (0.38; 0.71)	<0.0001	0.65 (0.45; 0.92)	0.016
**Wealth index:**				
Poorest	(ref)			
Poorer	0.74 (0.50; 1.10)	0.140		
Middle	0.79 (0.49; 1.25)	0.311		
Richer	0.48 (0.31; 0.76)	0.002		
Richest	0.52 (0.32; 0.84)	0.008		
Linear trend	0.84 (0.75; 0.94)	0.002	0.95 (0.83; 1.08)	0.396
**Total number of children in the household:**				
1–3	(ref)		(ref)	
4+	1.73 (1.23; 2.43)	0.002	1.52 (1.05; 2.20)	0.028
**Child age group (in months):**				
12–17	(ref)		(ref)	
18–23	0.76 (0.56; 1.04)	0.083	0.68 (0.49; 0.94)	0.021
**Husband/partner education level:**				
No education/primary	(ref)		(ref)	
Secondary or higher	1.06 (0.70; 1.59)	0.795	1.56 (1.00; 2.43)	0.048
Don’t know	2.44 (0.76; 7.83)	0.133	2.63 (0.84; 8.22)	0.096
**Ethnic group/language:**				
Shona	(ref)		(ref)	
Ndebele	0.57 (0.38; 0.86)	0.007	0.62 (0.39; 0.99)	0.047
English	0.60 (0.35; 1.02)	0.060	0.66 (0.37; 1.15)	0.143
**Place of delivery:**				
Any health facility	(ref)		(ref)	
At home	2.00 (1.44; 2.79)	<0.0001	1.44 (1.00; 2.07)	0.051
**Mother health check-up after delivery:**				
No	1.74 (1.26; 2.41)	0.001	1.34 (0.94; 1.89)	0.101
Yes	(ref)		(ref)	

This is Table 7 legend

*OR adjusted for the other variables in the table

§p-value from Wald Test.

Check for interaction between maternal living arrangements and all the variables included in the final model in turn revealed that none of these variables acted as an effect modifier. The same results were obtained when checking for any possible interaction between having received at least one injection of tetanus vaccination during pregnancy and whether the child lived with the mother or elsewhere (the strongest factors associated with not having a child fully vaccinated) and all the other variables of the model in turn.

## Discussion

### Summary of results, comparison with other studies

In the analysis of this dataset no association was found between maternal living arrangements and the vaccination status of the child. The proportion of fully vaccinated children in Zimbabwe does not differ between mothers who are married (or have a partner) and the partner/husband lives within the household, or lives away, or whether the mother is not married and has to bring up her children without the support of a partner. The results were practically the same in the crude and adjusted analysis, indicating that other variables did not confound the association. These results are in line with the cross-sectional studies of Ekra [10] in Niger, Faye [11] in rural Senegal, and Jagrati [12] in rural Mozambique, which also did not find any significant association between the mother’s marital status/maternal living arrangements and the vaccination status of their children. These results contrast however with those reported by Sackou [14] in Ivory Coast (peri-urban areas of Abidjan) and Caloun [15] in Western Kenya. Their cross-sectional studies found a strong association between being a single mother or living without a partner and their child not having been fully vaccinated. The contrast in results between this current study and those done in Ivory Coast and Kenya may be explained by the difference in contexts (including the fact that these studies were done in specific country areas), smaller sample size and adjustment for different confounders compared to this present study.

However, other important factors were identified as being associated with an incomplete vaccination status of children; factors that may act at different levels and through different mechanisms but mostly these factors related to the mothers. First of all, a mother who had received at least one dose of tetanus vaccination (as proxy of health seeking behaviour during pregnancy) was strongly associated with the complete vaccination of the child. This may also imply a “pro-vaccination” attitude of the mother as well as the possibility of having received health education from health professionals during antenatal care that may result in better vaccination coverage for children. Other indicators of health-seeking behaviour of the mother, namely place of delivery (whether the mother delivered at home instead of in a health facility) and mother’s check-up after delivery were also found to be associated with the outcome, even if other factors acted as positive confounders and the adjusted results were somehow weaker than the crude ones. These results are also confirmed by the association found with the mother’s level of education, an association also reported in other studies previously cited in this research [10, 11, 12, 14, 15], since a pro-vaccination attitude and positive health-seeking behaviour can be triggered by a higher level of education. Another strongly associated factor with full vaccination from this analysis was whether the child was actually living with the mother. This may indicate that the guardians, other than the parents, have less opportunities or willingness to vaccinate children who are not their owns. However, this factor could not be further explored since the survey data did not report where these children were actually living. The occupation of the mother and husband/partner, and the wealth index were not found to be associated with the vaccination status of children, and this is in contrast with what was found by Aemro in Ethiopia [13] where important vaccination coverage inequalities were reported concerning socio-economic status. Since this seems not to be the case in this study, it may be hypothesized that vaccination programmes in Zimbabwe are accessible to people independently from their socio-economic status and therefore the reasons for incomplete vaccination of children have to be sought elsewhere. In line with Calhoun [15], these results show an association between parity and complete vaccination of children and this may be explained by the fact that the more children a woman has then the less time and attention which can be dedicated to each of them also results in a poorer vaccination status of children. Although children should be fully vaccinated by their first birthday, the fact that children aged 18–23 months are more likely to be fully vaccinated than those aged 12–17 months may imply delays in following the correct vaccination calendar. Vaccination is therefore completed during campaigns or at other opportunities in health facilities when a child attends it for sickness, rather than the mother taking the child to a health facility only to be vaccinated. Interestingly, mothers who chose Shona as the language of the questionnaire, (in this analysis considered as a proxy indicator for ethnic group), appear to have their children less vaccinated than the Ndebele, and this fact should be further investigated.

### Strengths and limitations

This study has some strengths. Firstly, compared to other similar studies previously cited, this study includes a large sample size, derived from a large and countrywide survey, therefore its results can be generalizable for Zimbabwe as a whole. Secondly, it uses a more reliable tool for assessing vaccination coverage compared to official reports, since it is thought that survey data are more reliable that reports for this purpose. [9,21,22] Thirdly, the development of a conceptual hierarchical framework helped the categorisation of factors to be explored across different levels, as the survey dataset included a multifaceted number of variables. Lastly, this study contributes to evidence that mother’s education and parental health-seeking behaviour, as also found in other studies, improve the vaccination status of children [23], and other health outcomes. [24]

However, this study has also its limitations. Firstly, it is based on a survey; therefore data on exposure and outcome were collected simultaneously resulting in a non-fulfilment of the temporal criterion for causality. Secondly, the data collection to ascertain some factors, being on exposure or vaccination status, relied in part on the mother’s report and this may introduce information bias since mothers may be prone to give more socially-acceptable answers, (for example, on vaccination status of children), or may have problems in recalling vaccination events, (mostly for different types of antigens). Residual confounding cannot be ruled out: in spite of the numerous variables included in the models, it is still possible that the associations found with vaccination status may have been over or underestimated. Lastly, proxy indicators to estimate some factors may not be valid; in fact, mother’s health-seeking behaviour was estimated through tetanus vaccination during pregnancy, place of delivery and check-up after birth, and it cannot be excluded that these three factors may be more linked economically rather than measuring a proactive behaviour in seeking preventive health care. Similarly, the language of the questionnaire was used as the proxy for ethnic groups; while it can be assumed that Shona and Ndebele respondents belong to these respective ethnic groups, English speaking respondents may not necessarily be white since educated Shona and Ndebele people, or other minority groups, may have preferred English to answer the DHS questionnaire. This may have introduced some misclassification of this factor and the direction of this misclassification (whether differential or non-differential) remains unknown.

### Public health implications and recommended further research

Although this study reports that most of the children were fully vaccinated, the overall vaccination coverage, as well as the coverage for individual vaccines in Zimbabwe remains sub-optimal. In fact, although the measles vaccination coverage reaches about 78% (as per this study), Zimbabwe still recorded almost 10,000 cases of measles in 2012. [16] In the same year, measles accounted for about 1% of the total under 5 mortality in Zimbabwe, which was 90/1,000 live births (almost double the global average), and communicable diseases still represent 73% of the total years of life lost. [17]

In such a context it becomes necessary that vaccination coverage is improved to a level that childhood vaccine-preventable diseases are no longer a public health concern if these cannot be eradicated or eliminated (such as measles). The results of this study suggest that strategies to improve vaccination coverage should aim at improving ante-natal care for mothers, educating mothers on childhood vaccination, family planning and vaccination campaigns to include children who missed vaccination at the right period, although vaccination at the recommended age is preferable to avoid unnecessary susceptibility to these diseases. From a social and broader perspective, improving women’s educational level will have beneficial effects not only on the vaccination status of their children but also on other important health outcomes.[24]

The factors associated with incomplete vaccination in children identified in this study are far from exhaustive; therefore research in this field should continue to identify new risk factors with the aim of recommending strategies to improve vaccination coverage. Future studies should be more powerful compared to cross-sectional surveys, and preferably prospective in design.

## Supporting Information

S1 Questionnaire Questionnaire of the DHS-VI available at: http://www.measuredhs.com/publications/publication-DHSQ6-DHS-Questionnaires-and-Manuals.cfm. (PDF)
