# Supplementary material for: Do Maternal Living Arrangements Influence the Vaccination Status of Children Age 12–23 Months? A Data Analysis of Demographic Health Surveys 2010–11 from Zimbabwe
Source: PLoS One. 2015 Jul 13;10(7):e0132357. doi: 10.1371/journal.pone.0132357 (PMC4500504; doi:10.1371/journal.pone.0132357)
Supplement: S1 Questionnaire — (PDF) [file pone.0132357.s001.pdf]

USAID
FROM THE AMERICAN PEOPLE

QUESTIONNAIRES:

HOUSEHOLD, WOMAN'S, AND MAN'S

Demographic and Health Surveys Methodology

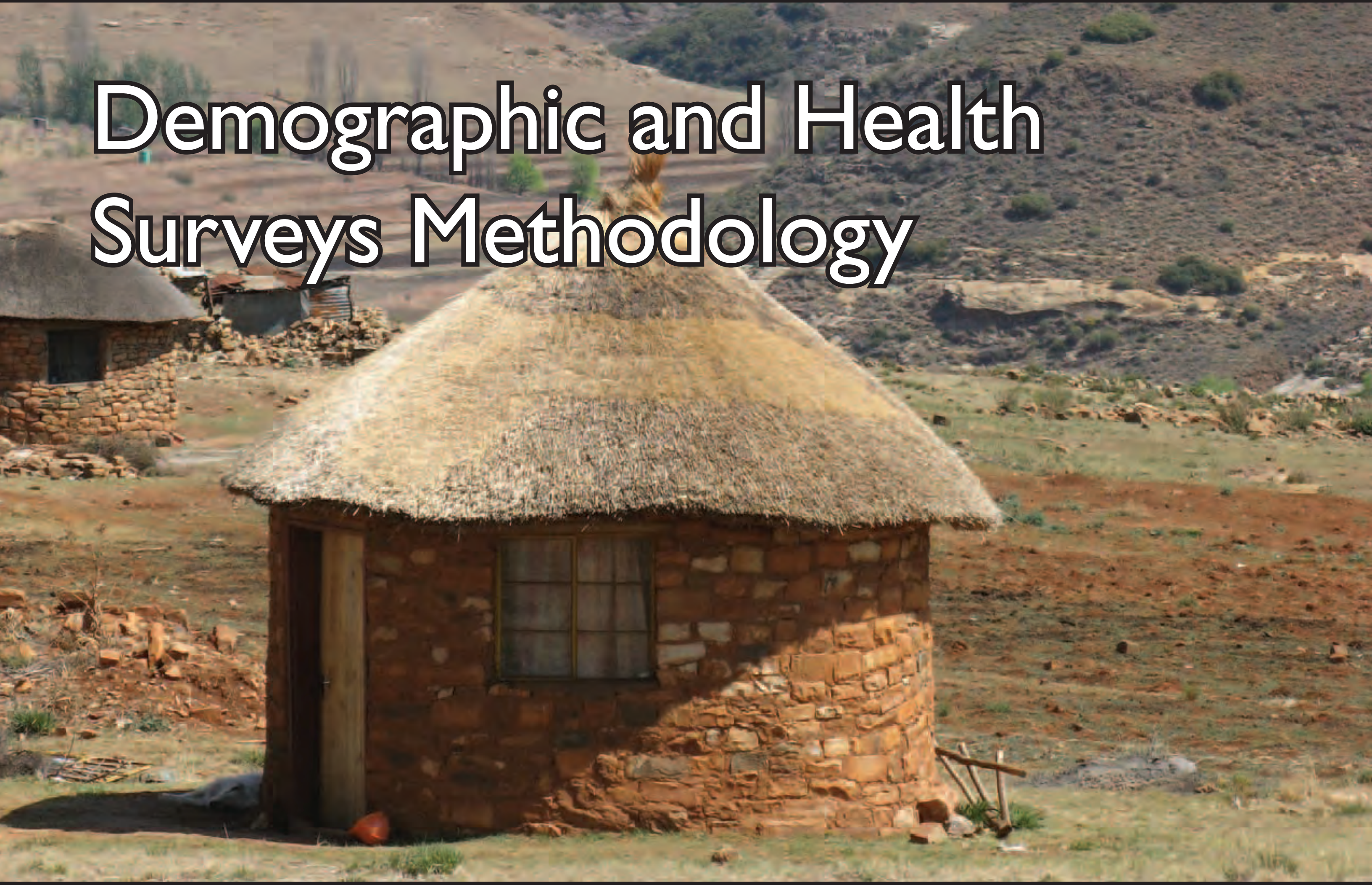

This document is part of the Demographic and Health Survey's *DHS Toolkit* of methodology for the MEASURE DHS Phase III project, implemented from 2008-2013.

This publication was produced for review by the United States Agency for International Development (USAID). It was prepared by MEASURE DHS/ICF International.

DHS TOOLKIT

[THIS PAGE IS INTENTIONALLY BLANK]

Demographic and Health Surveys Methodology

Questionnaires:
Household, Woman's, and Man's

ICF International
Calverton, Maryland

November 2011

MEASURE DHS is a five-year project to assist institutions in collecting and analyzing data needed to plan, monitor, and evaluate population, health, and nutrition programs. MEASURE DHS is funded by the U.S. Agency for International Development (USAID). The project is implemented by ICF International in Calverton, Maryland, in partnership with the Johns Hopkins Bloomberg School of Public Health/Center for Communication Programs, the Program for Appropriate Technology in Health (PATH), Futures Institute, Camris International, and Blue Raster.

The main objectives of the MEASURE DHS program are to: 1) provide improved information through appropriate data collection, analysis, and evaluation; 2) improve coordination and partnerships in data collection at the international and country levels; 3) increase host-country institutionalization of data collection capacity; 4) improve data collection and analysis tools and methodologies; and 5) improve the dissemination and utilization of data.

For information about the Demographic and Health Surveys (DHS) program, write to DHS, ICF International, 11785 Beltsville Drive, Suite 300, Calverton, MD 20705, U.S.A. (Telephone: 301-572-0200; fax: 301-572-0999; e-mail: info@measuredhs.com; Internet: http://www.measuredhs.com).

Recommended citation:
ICF International. 2011. Demographic and Health Surveys Methodology - Questionnaires: Household, Woman's, and Man's. MEASURE DHS Phase III: Calverton, Maryland, USA.
http://www.measuredhs.com/publications/publication-DHSQ6-DHS-Questionnaires-and-Manuals.cfm

5 November 2012

DEMOGRAPHIC AND HEALTH SURVEYS
MODEL HOUSEHOLD QUESTIONNAIRE

[NAME OF COUNTRY]
[NAME OF ORGANIZATION]

IDENTIFICATION (1)

PLACE NAME ______________________

NAME OF HOUSEHOLD HEAD ______________________

CLUSTER NUMBER . . . . [ ][ ][ ]

HOUSEHOLD NUMBER . . . . [ ][ ]

INTERVIEWER VISITS

	1	2	3	FINAL VISIT
DATE	________	________	________	DAY [ ][ ]<br>MONTH [ ][ ]<br>YEAR [ ][ ][ ][ ]
INTERVIEWER'S NAME	________	________	________	INT. NUMBER [ ][ ][ ]
RESULT*	________	________	________	RESULT [ ]
NEXT VISIT: DATE	________	________		TOTAL NUMBER OF VISITS [ ]
TIME	________	________		

*RESULT CODES:
1 COMPLETED
2 NO HOUSEHOLD MEMBER AT HOME OR NO COMPETENT RESPONDENT AT HOME AT TIME OF VISIT
3 ENTIRE HOUSEHOLD ABSENT FOR EXTENDED PERIOD OF TIME
4 POSTPONED
5 REFUSED
6 DWELLING VACANT OR ADDRESS NOT A DWELLING
7 DWELLING DESTROYED
8 DWELLING NOT FOUND
9 OTHER ______________________ (SPECIFY)

TOTAL PERSONS IN HOUSEHOLD [ ][ ]

TOTAL ELIGIBLE WOMEN [ ][ ]

TOTAL ELIGIBLE MEN [ ][ ]

LINE NO. OF RESPONDENT TO HOUSEHOLD QUESTIONNAIRE [ ][ ]

SUPERVISOR	FIELD EDITOR	OFFICE EDITOR	KEYED BY
NAME ________ [ ][ ][ ]	NAME ________ [ ][ ][ ]	[ ][ ]	[ ][ ]

Note: Questions with blue highlighting in the question number column are HIV related questions that may be deleted in some circumstances (see footnotes). Questions with pink highlighting in the question number column are malaria related questions that may be deleted in some circumstances (see footnotes).

THIS PAGE IS INTENTIONALLY BLANK

INTRODUCTION AND CONSENT

Hello. My name is ____. I am working with (NAME OF ORGANIZATION). We are conducting a survey about health all over (NAME OF COUNTRY). The information we collect will help the government to plan health services. Your household was selected for the survey. I would like to ask you some questions about your household. The questions usually take about 15 to 20 minutes. All of the answers you give will be confidential and will not be shared with anyone other than members of our survey team. You don't have to be in the survey, but we hope you will agree to answer the questions since your views are important. If I ask you any question you don't want to answer, just let me know and I will go on to the next question or you can stop the interview at any time.
In case you need more information about the survey, you may contact the person listed on this card.

GIVE CARD WITH CONTACT INFORMATION

Do you have any questions?
May I begin the interview now?

SIGNATURE OF INTERVIEWER: ________________________________ DATE: ____________________

RESPONDENT AGREES TO BE INTERVIEWED . . . 1 ↓ RESPONDENT DOES NOT AGREE TO BE INTERVIEWED . . . 2 → END

HOUSEHOLD SCHEDULE

							IF AGE 15 OR OLDER			
LINE NO.	USUAL RESIDENTS AND VISITORS	RELATIONSHIP TO HEAD OF HOUSEHOLD	SEX	RESIDENCE		AGE	MARITAL STATUS	ELIGIBILITY		
**1**	**2**	**3**	**4**	**5**	**6**	**7**	**8**	**9**	**10**	**11**
	Please give me the names of the persons who usually live in your household and guests of the household who stayed here last night, starting with the head of the household.<br><br>AFTER LISTING THE NAMES AND RECORDING THE RELATIONSHIP AND SEX FOR EACH PERSON, ASK QUESTIONS 2A-2C TO BE SURE THAT THE LISTING IS COMPLETE.<br><br>THEN ASK APPROPRIATE QUESTIONS IN COLUMNS 5-20 FOR EACH PERSON.	What is the relationship of (NAME) to the head of the household?<br><br>SEE CODES BELOW.	Is (NAME) male or female?	Does (NAME) usually live here?	Did (NAME) stay here last night?	How old is (NAME)?<br><br>IF 95 OR MORE, RECORD '95'.	What is (NAME)'s current marital status?<br><br>1 = MARRIED OR LIVING TOGETHER<br>2 = DIVORCED/ SEPARATED<br>3 = WIDOWED<br>4 = NEVER-MARRIED AND NEVER LIVED TOGETHER	CIRCLE LINE NUMBER OF ALL WOMEN AGE 15-49	CIRCLE LINE NUMBER OF ALL MEN AGE 15-49	CIRCLE LINE NUMBER OF ALL CHILDREN AGE 0-5
			M F	Y N	Y N	IN YEARS				
01		☐☐	1 2	1 2	1 2	☐☐	☐	01	01	01
02		☐☐	1 2	1 2	1 2	☐☐	☐	02	02	02
03		☐☐	1 2	1 2	1 2	☐☐	☐	03	03	03
04		☐☐	1 2	1 2	1 2	☐☐	☐	04	04	04
05		☐☐	1 2	1 2	1 2	☐☐	☐	05	05	05
06		☐☐	1 2	1 2	1 2	☐☐	☐	06	06	06
07		☐☐	1 2	1 2	1 2	☐☐	☐	07	07	07
08		☐☐	1 2	1 2	1 2	☐☐	☐	08	08	08
09		☐☐	1 2	1 2	1 2	☐☐	☐	09	09	09
10		☐☐	1 2	1 2	1 2	☐☐	☐	10	10	10

CODES FOR Q. 3: RELATIONSHIP TO HEAD OF HOUSEHOLD

01 = HEAD
02 = WIFE OR HUSBAND
03 = SON OR DAUGHTER
04 = SON-IN-LAW OR DAUGHTER-IN-LAW
05 = GRANDCHILD
06 = PARENT
07 = PARENT-IN-LAW
08 = BROTHER OR SISTER
09 = OTHER RELATIVE
10 = ADOPTED/FOSTER/ STEPCHILD
11 = NOT RELATED
98 = DON'T KNOW

	IF AGE 0-17 YEARS				IF AGE 5 YEARS OR OLDER		IF AGE 5-24 YEARS		IF AGE 0-4 YEARS
LINE NO.	SURVIVORSHIP AND RESIDENCE OF BIOLOGICAL PARENTS				EVER ATTENDED SCHOOL		CURRENT/RECENT SCHOOL ATTENDANCE		BIRTH REGIS-TRATION
	**12**	**13**	**14**	**15**	**16**	**17**	**18**	**19**	**20**
	Is (NAME)'s natural mother alive?	Does (NAME)'s natural mother usually live in this household or was she a guest last night?<br><br>IF YES: What is her name? RECORD MOTHER'S LINE NUMBER.<br><br>IF NO, RECORD '00'.	Is (NAME)'s natural father alive?	Does (NAME)'s natural father usually live in this household or was he a guest last night?<br><br>IF YES: What is his name? RECORD FATHER'S LINE NUMBER.<br><br>IF NO, RECORD '00'.	Has (NAME) ever attended school?	What is the highest level of school (NAME) has attended?<br><br>SEE CODES BELOW.<br><br>What is the highest grade (NAME) completed at that level?<br><br>SEE CODES BELOW.	Did (NAME) attend school at any time during the (2009-2010) **(2)** school year?	During this/that school year, what level and grade [is/was] (NAME) attending?<br><br>SEE CODES BELOW.	Does (NAME) have a birth certificate?<br><br>IF NO, PROBE: Has (NAME)'s birth ever been registered with the civil authority?<br><br>1 = HAS CERTIFICATE<br>2 = REGISTERED<br>3 = NEITHER<br>8 = DON'T KNOW
01	Y N DK<br>1 2 8<br>GO TO 14	☐☐	Y N DK<br>1 2 8<br>GO TO 16	☐☐	Y N<br>1 2<br>NEXT LINE	LEVEL GRADE<br>☐ ☐☐	Y N<br>1 2<br>NEXT LINE	LEVEL GRADE<br>☐ ☐☐	☐
02	1 2 8<br>GO TO 14	☐☐	1 2 8<br>GO TO 16	☐☐	1 2<br>NEXT LINE	☐ ☐☐	1 2<br>NEXT LINE	☐ ☐☐	☐
03	1 2 8<br>GO TO 14	☐☐	1 2 8<br>GO TO 16	☐☐	1 2<br>NEXT LINE	☐ ☐☐	1 2<br>NEXT LINE	☐ ☐☐	☐
04	1 2 8<br>GO TO 14	☐☐	1 2 8<br>GO TO 16	☐☐	1 2<br>NEXT LINE	☐ ☐☐	1 2<br>NEXT LINE	☐ ☐☐	☐
05	1 2 8<br>GO TO 14	☐☐	1 2 8<br>GO TO 16	☐☐	1 2<br>NEXT LINE	☐ ☐☐	1 2<br>NEXT LINE	☐ ☐☐	☐
06	1 2 8<br>GO TO 14	☐☐	1 2 8<br>GO TO 16	☐☐	1 2<br>NEXT LINE	☐ ☐☐	1 2<br>NEXT LINE	☐ ☐☐	☐
07	1 2 8<br>GO TO 14	☐☐	1 2 8<br>GO TO 16	☐☐	1 2<br>NEXT LINE	☐ ☐☐	1 2<br>NEXT LINE	☐ ☐☐	☐
08	1 2 8<br>GO TO 14	☐☐	1 2 8<br>GO TO 16	☐☐	1 2<br>NEXT LINE	☐ ☐☐	1 2<br>NEXT LINE	☐ ☐☐	☐
09	1 2 8<br>GO TO 14	☐☐	1 2 8<br>GO TO 16	☐☐	1 2<br>NEXT LINE	☐ ☐☐	1 2<br>NEXT LINE	☐ ☐☐	☐
10	1 2 8<br>GO TO 14	☐☐	1 2 8<br>GO TO 16	☐☐	1 2<br>NEXT LINE	☐ ☐☐	1 2<br>NEXT LINE	☐ ☐☐	☐

CODES FOR Qs. 17 AND 19: EDUCATION

LEVEL	GRADE
1 = PRIMARY	00 = LESS THAN 1 YEAR COMPLETED (USE '00' FOR Q. 17 ONLY. THIS CODE IS NOT ALLOWED FOR Q. 19)
2 = SECONDARY	98 = DON'T KNOW
3 = HIGHER	
6 = PRE-PRIMARY	
8 = DON'T KNOW	

							IF AGE 15 OR OLDER			
LINE NO.	USUAL RESIDENTS AND VISITORS	RELATIONSHIP TO HEAD OF HOUSEHOLD	SEX	RESIDENCE		AGE	MARITAL STATUS	ELIGIBILITY		
**1**	**2**	**3**	**4**	**5**	**6**	**7**	**8**	**9**	**10**	**11**
	Please give me the names of the persons who usually live in your household and guests of the household who stayed here last night, starting with the head of the household.<br><br>AFTER LISTING THE NAMES AND RECORDING THE RELATIONSHIP AND SEX FOR EACH PERSON, ASK QUESTIONS 2A-2C TO BE SURE THAT THE LISTING IS COMPLETE.<br><br>THEN ASK APPROPRIATE QUESTIONS IN COLUMNS 5-20 FOR EACH PERSON.	What is the relationship of (NAME) to the head of the household?<br><br>SEE CODES BELOW.	Is (NAME) male or female?	Does (NAME) usually live here?	Did (NAME) stay here last night?	How old is (NAME)?<br><br>IF 95 OR MORE, RECORD '95'.	What is (NAME)'s current marital status?<br><br>1 = MARRIED OR LIVING TOGETHER<br>2 = DIVORCED/ SEPARATED<br>3 = WIDOWED<br>4 = NEVER-MARRIED AND NEVER LIVED TOGETHER	CIRCLE LINE NUMBER OF ALL WOMEN AGE 15-49	CIRCLE LINE NUMBER OF ALL MEN AGE 15-49	CIRCLE LINE NUMBER OF ALL CHILDREN AGE 0-5
			M F	Y N	Y N	IN YEARS				
11		☐☐	1 2	1 2	1 2	☐☐	☐	11	11	11
12		☐☐	1 2	1 2	1 2	☐☐	☐	12	12	12
13		☐☐	1 2	1 2	1 2	☐☐	☐	13	13	13
14		☐☐	1 2	1 2	1 2	☐☐	☐	14	14	14
15		☐☐	1 2	1 2	1 2	☐☐	☐	15	15	15
16		☐☐	1 2	1 2	1 2	☐☐	☐	16	16	16
17		☐☐	1 2	1 2	1 2	☐☐	☐	17	17	17
18		☐☐	1 2	1 2	1 2	☐☐	☐	18	18	18
19		☐☐	1 2	1 2	1 2	☐☐	☐	19	19	19
20		☐☐	1 2	1 2	1 2	☐☐	☐	20	20	20

TICK HERE IF CONTINUATION SHEET USED ☐

2A) Just to make sure that I have a complete listing: are there any other persons such as small children or infants that we have not listed? YES ☐ → ADD TO TABLE NO ☐

2B) Are there any other people who may not be members of your family, such as domestic servants, lodgers, or friends who usually live here? YES ☐ → ADD TO TABLE NO ☐

2C) Are there any guests or temporary visitors staying here, or anyone else who stayed here last night, who have not been listed? YES ☐ → ADD TO TABLE NO ☐

CODES FOR Q. 3: RELATIONSHIP TO HEAD OF HOUSEHOLD

01 = HEAD
02 = WIFE OR HUSBAND
03 = SON OR DAUGHTER
04 = SON-IN-LAW OR DAUGHTER-IN-LAW
05 = GRANDCHILD
06 = PARENT
07 = PARENT-IN-LAW
08 = BROTHER OR SISTER
09 = OTHER RELATIVE
10 = ADOPTED/FOSTER/ STEPCHILD
11 = NOT RELATED
98 = DON'T KNOW

LINE NO.	IF AGE 0-17 YEARS<br>SURVIVORSHIP AND RESIDENCE OF BIOLOGICAL PARENTS				IF AGE 5 YEARS OR OLDER<br>EVER ATTENDED SCHOOL		IF AGE 5-24 YEARS<br>CURRENT/RECENT SCHOOL ATTENDANCE		IF AGE 0-4 YEARS<br>BIRTH REGIS-TRATION
	**12**	**13**	**14**	**15**	**16**	**17**	**18**	**19**	**20**
	Is (NAME)'s natural mother alive?	Does (NAME)'s natural mother usually live in this household or was she a guest last night?<br><br>IF YES: What is her name? RECORD MOTHER'S LINE NUMBER.<br><br>IF NO, RECORD '00'.	Is (NAME)'s natural father alive?	Does (NAME)'s natural father usually live in this household or was he a guest last night?<br><br>IF YES: What is his name? RECORD FATHER'S LINE NUMBER.<br><br>IF NO, RECORD '00'.	Has (NAME) ever attended school?	What is the highest level of school (NAME) has attended?<br><br>SEE CODES BELOW.<br><br>What is the highest grade (NAME) completed at that level?<br><br>SEE CODES BELOW.	Did (NAME) attend school at any time during the (2009-2010) **(2)** school year?	During this/that school year, what level and grade [is/was] (NAME) attending?<br><br>SEE CODES BELOW.	Does (NAME) have a birth certificate?<br><br>IF NO, PROBE: Has (NAME)'s birth ever been registered with the civil authority?<br><br>1 = HAS CERTIFICATE<br>2 = REGISTERED<br>3 = NEITHER<br>8 = DON'T KNOW
	Y N DK		Y N DK		Y N	LEVEL GRADE	Y N	LEVEL GRADE	
11	1 2 8<br>GO TO 14	☐☐	1 2 8<br>GO TO 16	☐☐	1 2<br>NEXT LINE	☐ ☐☐	1 2<br>NEXT LINE	☐ ☐☐	☐
12	1 2 8<br>GO TO 14	☐☐	1 2 8<br>GO TO 16	☐☐	1 2<br>NEXT LINE	☐ ☐☐	1 2<br>NEXT LINE	☐ ☐☐	☐
13	1 2 8<br>GO TO 14	☐☐	1 2 8<br>GO TO 16	☐☐	1 2<br>NEXT LINE	☐ ☐☐	1 2<br>NEXT LINE	☐ ☐☐	☐
14	1 2 8<br>GO TO 14	☐☐	1 2 8<br>GO TO 16	☐☐	1 2<br>NEXT LINE	☐ ☐☐	1 2<br>NEXT LINE	☐ ☐☐	☐
15	1 2 8<br>GO TO 14	☐☐	1 2 8<br>GO TO 16	☐☐	1 2<br>NEXT LINE	☐ ☐☐	1 2<br>NEXT LINE	☐ ☐☐	☐
16	1 2 8<br>GO TO 14	☐☐	1 2 8<br>GO TO 16	☐☐	1 2<br>NEXT LINE	☐ ☐☐	1 2<br>NEXT LINE	☐ ☐☐	☐
17	1 2 8<br>GO TO 14	☐☐	1 2 8<br>GO TO 16	☐☐	1 2<br>NEXT LINE	☐ ☐☐	1 2<br>NEXT LINE	☐ ☐☐	☐
18	1 2 8<br>GO TO 14	☐☐	1 2 8<br>GO TO 16	☐☐	1 2<br>NEXT LINE	☐ ☐☐	1 2<br>NEXT LINE	☐ ☐☐	☐
19	1 2 8<br>GO TO 14	☐☐	1 2 8<br>GO TO 16	☐☐	1 2<br>NEXT LINE	☐ ☐☐	1 2<br>NEXT LINE	☐ ☐☐	☐
20	1 2 8<br>GO TO 14	☐☐	1 2 8<br>GO TO 16	☐☐	1 2<br>NEXT LINE	☐ ☐☐	1 2<br>NEXT LINE	☐ ☐☐	☐

CODES FOR Qs. 17 AND 19: EDUCATION

**LEVEL**	**GRADE**
1 = PRIMARY	00 = LESS THAN 1 YEAR COMPLETED
2 = SECONDARY	(USE '00' FOR Q. 17 ONLY.
3 = HIGHER	THIS CODE IS NOT ALLOWED
6 = PRE-PRIMARY	FOR Q. 19)
8 = DON'T KNOW	98 = DON'T KNOW

HOUSEHOLD CHARACTERISTICS

NO.	QUESTIONS AND FILTERS	CODING CATEGORIES	SKIP
101	How often does anyone smoke inside your house?<br>Would you say daily, weekly, monthly, less than monthly, or never?	DAILY . . . . . 1<br>WEEKLY . . . . . 2<br>MONTHLY . . . . . 3<br>LESS THAN MONTHLY . . . . . 4<br>NEVER 5	
102	What is the main source of drinking water for members of your household?	PIPED WATER<br>PIPED INTO DWELLING . . . . . 11<br>PIPED TO YARD/PLOT . . . . . 12<br>PUBLIC TAP/STANDPIPE . . . . . 13<br>TUBE WELL OR BOREHOLE . . . . . 21<br>DUG WELL<br>PROTECTED WELL . . . . . 31<br>UNPROTECTED WELL . . . . . 32<br>WATER FROM SPRING<br>PROTECTED SPRING . . . . . 41<br>UNPROTECTED SPRING . . . . . 42<br>RAINWATER . . . . . 51<br>TANKER TRUCK . . . . . 61<br>CART WITH SMALL TANK . . . . . 71<br>SURFACE WATER (RIVER/DAM/ LAKE/POND/STREAM/CANAL/ IRRIGATION CHANNEL) . . . . . 81<br>BOTTLED WATER . . . . . 91<br>OTHER ______ 96<br>(SPECIFY)	11, 12 → 105<br>51 → 105
103	Where is that water source located?	IN OWN DWELLING . . . . . 1<br>IN OWN YARD/PLOT . . . . . 2<br>ELSEWHERE 3	1, 2 → 105
104	How long does it take to go there, get water, and come back?	MINUTES . . . . . [ ][ ][ ]<br>DON'T KNOW 998	
105	Do you do anything to the water to make it safer to drink?	YES . . . . . 1<br>NO . . . . . 2<br>DON'T KNOW 8	2, 8 → 107
106	What do you usually do to make the water safer to drink?<br><br>Anything else?<br><br>RECORD ALL MENTIONED.	BOIL . . . . . A<br>ADD BLEACH/CHLORINE . . . . . B<br>STRAIN THROUGH A CLOTH . . . . . C<br>USE WATER FILTER (CERAMIC/ SAND/COMPOSITE/ETC.) . . . . . D<br>SOLAR DISINFECTION . . . . . E<br>LET IT STAND AND SETTLE . . . . . F<br>OTHER ______ X<br>(SPECIFY)<br>DON'T KNOW . . . . . Z	

NO.	QUESTIONS AND FILTERS	CODING CATEGORIES	SKIP
107	What kind of toilet facility do members of your household usually use? **(3)**	FLUSH OR POUR FLUSH TOILET<br>FLUSH TO PIPED SEWER SYSTEM . . . . . 11<br>FLUSH TO SEPTIC TANK . . . . . 12<br>FLUSH TO PIT LATRINE . . . . . 13<br>FLUSH TO SOMEWHERE ELSE . . . . . 14<br>FLUSH, DON'T KNOW WHERE . . . . . 15<br>PIT LATRINE<br>VENTILATED IMPROVED PIT LATRINE . . . . . 21<br>PIT LATRINE WITH SLAB . . . . . 22<br>PIT LATRINE WITHOUT SLAB/ OPEN PIT . . . . . 23<br>COMPOSTING TOILET . . . . . 31<br>BUCKET TOILET . . . . . 41<br>HANGING TOILET/HANGING LATRINE . . . . . 51<br>NO FACILITY/BUSH/FIELD . . . . . 61<br>OTHER ______ (SPECIFY) 96	61 → 110
108	Do you share this toilet facility with other households?	YES . . . . . 1<br>NO 2	2 → 110
109	How many households use this toilet facility?	NO. OF HOUSEHOLDS IF LESS THAN 10 . . . . . [0][ ]<br>10 OR MORE HOUSEHOLDS . . . . . 95<br>DON'T KNOW 98	
110	Does your household have: **(4)**<br>Electricity?<br>A radio?<br>A television?<br>A mobile telephone?<br>A non-mobile telephone?<br>A refrigerator?<br>[ADD ADDITIONAL ITEMS. SEE FOOTNOTE 4.]	YES NO<br>ELECTRICITY . . . . . 1 2<br>RADIO . . . . . 1 2<br>TELEVISION . . . . . 1 2<br>MOBILE TELEPHONE . . . . . 1 2<br>NON-MOBILE TELEPHONE . . . 1 2<br>REFRIGERATOR 1 2	
111	What type of fuel does your household mainly use for cooking?	ELECTRICITY . . . . . 01<br>LPG . . . . . 02<br>NATURAL GAS . . . . . 03<br>BIOGAS . . . . . 04<br>KEROSENE . . . . . 05<br>COAL, LIGNITE . . . . . 06<br>CHARCOAL . . . . . 07<br>WOOD . . . . . 08<br>STRAW/SHRUBS/GRASS . . . . . 09<br>AGRICULTURAL CROP . . . . . 10<br>ANIMAL DUNG . . . . . 11<br>NO FOOD COOKED IN HOUSEHOLD . . . . . 95<br>OTHER ______ (SPECIFY) 96	95 → 114

NO.	QUESTIONS AND FILTERS	CODING CATEGORIES	SKIP
112	Is the cooking usually done in the house, in a separate building, or outdoors?	IN THE HOUSE . . . . 1<br>IN A SEPARATE BUILDING . . . . 2<br>OUTDOORS . . . . 3<br><br>OTHER ______________________ 6<br>(SPECIFY)	2, 3, 6 → 114
113	Do you have a separate room which is used as a kitchen?	YES . . . . 1<br>NO 2	
114	MAIN MATERIAL OF THE FLOOR. **(3)**<br><br>RECORD OBSERVATION.	NATURAL FLOOR<br>EARTH/SAND . . . . 11<br>DUNG . . . . 12<br>RUDIMENTARY FLOOR<br>WOOD PLANKS . . . . 21<br>PALM/BAMBOO . . . . 22<br>FINISHED FLOOR<br>PARQUET OR POLISHED<br>WOOD . . . . 31<br>VINYL OR ASPHALT STRIPS . . . . 32<br>CERAMIC TILES . . . . 33<br>CEMENT . . . . 34<br>CARPET . . . . 35<br><br>OTHER ______________________ 96<br>(SPECIFY)	
115	MAIN MATERIAL OF THE ROOF. **(3)**<br><br>RECORD OBSERVATION.	NATURAL ROOFING<br>NO ROOF . . . . 11<br>THATCH/PALM LEAF . . . . 12<br>SOD . . . . 13<br>RUDIMENTARY ROOFING<br>RUSTIC MAT . . . . 21<br>PALM/BAMBOO . . . . 22<br>WOOD PLANKS . . . . 23<br>CARDBOARD . . . . 24<br>FINISHED ROOFING<br>METAL . . . . 31<br>WOOD . . . . 32<br>CALAMINE/CEMENT FIBER . . . . 33<br>CERAMIC TILES . . . . 34<br>CEMENT . . . . 35<br>ROOFING SHINGLES . . . . 36<br><br>OTHER ______________________ 96<br>(SPECIFY)	

NO.	QUESTIONS AND FILTERS	CODING CATEGORIES	SKIP
116	MAIN MATERIAL OF THE EXTERIOR WALLS. **(3)**<br><br>RECORD OBSERVATION.	NATURAL WALLS<br>NO WALLS . . . . 11<br>CANE/PALM/TRUNKS . . . . 12<br>DIRT . . . . 13<br>RUDIMENTARY WALLS<br>BAMBOO WITH MUD . . . . 21<br>STONE WITH MUD . . . . 22<br>UNCOVERED ADOBE . . . . 23<br>PLYWOOD . . . . 24<br>CARDBOARD . . . . 25<br>REUSED WOOD . . . . 26<br>FINISHED WALLS<br>CEMENT . . . . 31<br>STONE WITH LIME/CEMENT . . . . 32<br>BRICKS . . . . 33<br>CEMENT BLOCKS . . . . 34<br>COVERED ADOBE . . . . 35<br>WOOD PLANKS/SHINGLES . . . . 36<br><br>OTHER ______________________ 96<br>(SPECIFY)	
117	How many rooms in this household are used for sleeping?	ROOMS . . . . [ ][ ]	
118	Does any member of this household own:<br><br>A watch?<br>A bicycle?<br>A motorcycle or motor scooter?<br>An animal-drawn cart?<br>A car or truck?<br>A boat with a motor?	YES NO<br>WATCH . . . . 1 2<br>BICYCLE . . . . 1 2<br>MOTORCYCLE/SCOOTER . . . 1 2<br>ANIMAL-DRAWN CART . . . . . 1 2<br>CAR/TRUCK . . . . 1 2<br>BOAT WITH MOTOR 1 2	
119	Does any member of this household own any agricultural land?	YES . . . . 1<br>NO 2	 → 121
120	How many hectares of agricultural land do members of this household own?<br><br>IF 95 OR MORE, CIRCLE '950'.	HECTARES . . . . [ ][ ].[ ]<br><br>95 OR MORE HECTARES . . . . 950<br>DON'T KNOW 998	
121	Does this household own any livestock, herds, other farm animals, or poultry?	YES . . . . 1<br>NO 2	 → 123

NO.	QUESTIONS AND FILTERS	CODING CATEGORIES	SKIP
122	How many of the following animals does this household own? **(5)**<br>IF NONE, ENTER '00'.<br>IF 95 OR MORE, ENTER '95'.<br>IF UNKNOWN, ENTER '98'.<br><br>Cattle?<br>Milk cows or bulls?<br>Horses, donkeys, or mules?<br>Goats?<br>Sheep?<br>Chickens?	 <br><br><br><br>CATTLE . . . . ☐☐<br>COWS/BULLS . . . . ☐☐<br>HORSES/DONKEYS/MULES . . . . . ☐☐<br>GOATS . . . . ☐☐<br>SHEEP . . . . ☐☐<br>CHICKENS . . . . ☐☐	
123	Does any member of this household have a bank account?	YES . . . . 1<br>NO 2	
124<br>**(6)**	At any time in the past 12 months, has anyone come into your dwelling to spray the interior walls against mosquitoes?	YES . . . . 1<br>NO . . . . 2<br>DON'T KNOW 8	 ⌉<br>→ 126
125<br>**(6)**	Who sprayed the dwelling?	GOVERNMENT WORKER/PROGRAM . . . . . A<br>PRIVATE COMPANY . . . . B<br>NONGOVERNMENTAL<br>ORGANIZATION (NGO) . . . . C<br><br>OTHER ______________________ X<br>(SPECIFY)<br>DON'T KNOW . . . . Z	
126<br>**(7)**	Does your household have any mosquito nets that can be used while sleeping?	YES . . . . 1<br>NO 2	 → 137
127<br>**(7)**	How many mosquito nets does your household have?<br><br>IF 7 OR MORE NETS, RECORD '7'.	NUMBER OF NETS . . . . ☐	

		NET #1	NET #2	NET #3
128<br>**(7)**	ASK THE RESPONDENT TO SHOW YOU ALL THE NETS IN THE HOUSEHOLD<br><br>IF MORE THAN 3 NETS, USE ADDITIONAL QUESTIONNAIRE(S).	OBSERVED . . . . . 1<br>NOT OBSERVED . . . 2	OBSERVED . . . . . 1<br>NOT OBSERVED . . . 2	OBSERVED . . . . . 1<br>NOT OBSERVED . . . 2
129<br>**(7)**	How many months ago did your household get the mosquito net?<br><br>IF LESS THAN ONE MONTH AGO, RECORD '00'.	MONTHS AGO . . . [ ][ ]<br><br>MORE THAN 36 MONTHS AGO . . . 95<br><br>NOT SURE 98	MONTHS AGO . . . [ ][ ]<br><br>MORE THAN 36 MONTHS AGO . . . 95<br><br>NOT SURE 98	MONTHS AGO . . . [ ][ ]<br><br>MORE THAN 36 MONTHS AGO . . . 95<br><br>NOT SURE 98
130<br>**(7)**	OBSERVE OR ASK THE BRAND/ TYPE OF MOSQUITO NET.<br><br>IF BRAND IS UNKNOWN AND YOU CANNOT OBSERVE THE NET, SHOW PICTURES OF TYPICAL NET TYPES/BRANDS TO RESPONDENT.	LONG-LASTING INSECTICIDE-TREATED NET (LLIN)<br>BRAND A . . . . . 11<br>BRAND B . . . . . 12<br>OTHER/ DK BRAND . . . 16<br>(SKIP TO 134)<br><br>'PRETREATED' NET<br>BRAND C . . . . . 21<br>BRAND D . . . . . 22<br>OTHER/ DK BRAND . . . 26<br>(SKIP TO 132)<br><br>OTHER BRAND . . . 96<br>DK BRAND 98	LONG-LASTING INSECTICIDE-TREATED NET (LLIN)<br>BRAND A . . . . . 11<br>BRAND B . . . . . 12<br>OTHER/ DK BRAND . . . 16<br>(SKIP TO 134)<br><br>'PRETREATED' NET<br>BRAND C . . . . . 21<br>BRAND D . . . . . 22<br>OTHER/ DK BRAND . . . 26<br>(SKIP TO 132)<br><br>OTHER BRAND . . . 96<br>DK BRAND 98	LONG-LASTING INSECTICIDE-TREATED NET (LLIN)<br>BRAND A . . . . . 11<br>BRAND B . . . . . 12<br>OTHER/ DK BRAND . . . 16<br>(SKIP TO 134)<br><br>'PRETREATED' NET<br>BRAND C . . . . . 21<br>BRAND D . . . . . 22<br>OTHER/ DK BRAND . . . 26<br>(SKIP TO 132)<br><br>OTHER BRAND . . . 96<br>DK BRAND 98
131<br>**(7)**	When you got the net, was it already treated with an insecticide to kill or repel mosquitoes?	YES . . . . 1<br>NO . . . . 2<br>NOT SURE 8	YES . . . . 1<br>NO . . . . 2<br>NOT SURE 8	YES . . . . 1<br>NO . . . . 2<br>NOT SURE 8
132<br>**(7)**	Since you got the net, was it ever soaked or dipped in a liquid to kill or repel mosquitoes?	YES . . . . 1<br>NO . . . . 2<br>(SKIP TO 134)<br>NOT SURE 8	YES . . . . 1<br>NO . . . . 2<br>(SKIP TO 134)<br>NOT SURE 8	YES . . . . 1<br>NO . . . . 2<br>(SKIP TO 134)<br>NOT SURE 8
133<br>**(7)**	How many months ago was the net last soaked or dipped?<br>IF LESS THAN ONE MONTH AGO, RECORD '00'.	MONTHS AGO . . . [ ][ ]<br><br>MORE THAN 24 MONTHS AGO . . . 95<br><br>NOT SURE 98	MONTHS AGO . . . [ ][ ]<br><br>MORE THAN 24 MONTHS AGO . . . 95<br><br>NOT SURE 98	MONTHS AGO . . . [ ][ ]<br><br>MORE THAN 24 MONTHS AGO . . . 95<br><br>NOT SURE 98
134<br>**(7)**	Did anyone sleep under this mosquito net last night?	YES . . . . 1<br>NO . . . . 2<br>(SKIP TO 136)<br>NOT SURE 8	YES . . . . 1<br>NO . . . . 2<br>(SKIP TO 136)<br>NOT SURE 8	YES . . . . 1<br>NO . . . . 2<br>(SKIP TO 136)<br>NOT SURE 8

		NET #1	NET #2	NET #3
135 **(7)**	Who slept under this mosquito net last night?<br><br>RECORD THE PERSON'S NAME AND LINE NUMBER FROM THE HOUSEHOLD SCHEDULE.	NAME_____________<br>LINE NO. . . . . . [ ][ ]<br>NAME_____________<br>LINE NO. . . . . . [ ][ ]<br>NAME_____________<br>LINE NO. . . . . . [ ][ ]<br>NAME_____________<br>LINE NO. . . . . . [ ][ ]	NAME_____________<br>LINE NO. . . . . . [ ][ ]<br>NAME_____________<br>LINE NO. . . . . . [ ][ ]<br>NAME_____________<br>LINE NO. . . . . . [ ][ ]<br>NAME_____________<br>LINE NO. . . . . . [ ][ ]	NAME_____________<br>LINE NO. . . . . . [ ][ ]<br>NAME_____________<br>LINE NO. . . . . . [ ][ ]<br>NAME_____________<br>LINE NO. . . . . . [ ][ ]<br>NAME_____________<br>LINE NO. . . . . . [ ][ ]
136 **(7)**		GO BACK TO 128 FOR NEXT NET; OR, IF NO MORE NETS, GO TO 137.	GO BACK TO 128 FOR NEXT NET; OR, IF NO MORE NETS, GO TO 137.	GO TO 128 IN FIRST COLUMN OF A NEW QUESTIONNAIRE; OR, IF NO MORE NETS, GO TO 137.

137	Please show me where members of your household most often wash their hands.	OBSERVED . . . . 1<br>NOT OBSERVED,<br>NOT IN DWELLING/YARD/PLOT . . . . 2<br>NOT OBSERVED,<br>NO PERMISSION TO SEE . . . . 3<br>NOT OBSERVED, OTHER REASON 4	2, 3, 4: (SKIP TO 140)
138	OBSERVATION ONLY:<br><br>OBSERVE PRESENCE OF WATER AT THE PLACE FOR HANDWASHING.	WATER IS AVAILABLE . . . . 1<br>WATER IS NOT AVAILABLE 2	
139	OBSERVATION ONLY:<br><br>OBSERVE PRESENCE OF SOAP, DETERGENT, OR OTHER CLEANSING AGENT.	SOAP OR DETERGENT<br>(BAR, LIQUID, POWDER, PASTE) . . . . A<br>ASH, MUD, SAND . . . . B<br>NONE C	
140	ASK RESPONDENT FOR A TEASPOONFUL OF COOKING SALT.<br><br>TEST SALT FOR IODINE. **(8)**	IODINE PRESENT . . . . 1<br>NO IODINE . . . . 2<br><br>NO SALT IN HOUSEHOLD . . . . 3<br><br>SALT NOT TESTED ______________________ 6<br>(SPECIFY REASON)	

WEIGHT, HEIGHT AND HEMOGLOBIN MEASUREMENT FOR CHILDREN AGE 0-5

201	CHECK COLUMN 11 IN HOUSEHOLD SCHEDULE. RECORD THE LINE NUMBER AND NAME FOR ALL ELIGIBLE CHILDREN 0-5 YEARS IN QUESTION 202. IF MORE THAN SIX CHILDREN, USE ADDITIONAL QUESTIONNAIRE(S).			
		CHILD 1	CHILD 2	CHILD 3
202	LINE NUMBER FROM COLUMN 11<br>NAME FROM COLUMN 2	LINE NUMBER . . . . . . [ ][ ]<br>NAME ________	LINE NUMBER . . . . . . [ ][ ]<br>NAME ________	LINE NUMBER . . . . . . [ ][ ]<br>NAME ________
203	IF MOTHER INTERVIEWED, COPY MONTH AND YEAR OF BIRTH FROM BIRTH HISTORY AND ASK DAY; IF MOTHER NOT INTERVIEWED, ASK:<br>What is (NAME)'s birth date?	DAY . . . . [ ][ ]<br>MONTH . . . . . . [ ][ ]<br>YEAR [ ][ ][ ][ ]	DAY . . . . [ ][ ]<br>MONTH . . . . . . [ ][ ]<br>YEAR [ ][ ][ ][ ]	DAY . . . . [ ][ ]<br>MONTH . . . . . . [ ][ ]<br>YEAR [ ][ ][ ][ ]
204	CHECK 203:<br>CHILD BORN IN JANUARY 2005 **(9)** OR LATER?	YES . . . . 1<br>NO . . . . 2<br>(GO TO 203 FOR NEXT CHILD OR, IF NO MORE CHILDREN, GO TO 214)	YES . . . . 1<br>NO . . . . 2<br>(GO TO 203 FOR NEXT CHILD OR, IF NO MORE CHILDREN, GO TO 214)	YES . . . . 1<br>NO . . . . 2<br>(GO TO 203 FOR NEXT CHILD OR, IF NO MORE CHILDREN, GO TO 214)
205	WEIGHT IN KILOGRAMS<br>**(10)**	KG. [ ][ ] . [ ][ ]<br>NOT PRESENT . . . 9994<br>REFUSED . . . . 9995<br>OTHER 9996	KG. [ ][ ] . [ ][ ]<br>NOT PRESENT . . . 9994<br>REFUSED . . . . 9995<br>OTHER 9996	KG. [ ][ ] . [ ][ ]<br>NOT PRESENT . . . 9994<br>REFUSED . . . . 9995<br>OTHER 9996
206	HEIGHT IN CENTIMETERS	CM. [ ][ ][ ] . [ ]<br>NOT PRESENT . . . 9994<br>REFUSED . . . . 9995<br>OTHER 9996	CM. [ ][ ][ ] . [ ]<br>NOT PRESENT . . . 9994<br>REFUSED . . . . 9995<br>OTHER 9996	CM. [ ][ ][ ] . [ ]<br>NOT PRESENT . . . 9994<br>REFUSED . . . . 9995<br>OTHER 9996
207	MEASURED LYING DOWN OR STANDING UP?	LYING DOWN . . . . 1<br>STANDING UP . . . . 2<br>NOT MEASURED 3	LYING DOWN . . . . 1<br>STANDING UP . . . . 2<br>NOT MEASURED 3	LYING DOWN . . . . 1<br>STANDING UP . . . . 2<br>NOT MEASURED 3
208	CHECK 203:<br>IS CHILD AGE 0-5 MONTHS, I.E., WAS CHILD BORN IN MONTH OF INTERVIEW OR FIVE PREVIOUS MONTHS?	0-5 MONTHS . . . . 1<br>(GO TO 203 FOR NEXT CHILD OR, IF NO MORE CHILDREN, GO TO 214)<br>OLDER 2	0-5 MONTHS . . . . 1<br>(GO TO 203 FOR NEXT CHILD OR, IF NO MORE CHILDREN, GO TO 214)<br>OLDER 2	0-5 MONTHS . . . . 1<br>(GO TO 203 FOR NEXT CHILD OR, IF NO MORE CHILDREN, GO TO 214)<br>OLDER 2
209	LINE NUMBER OF PARENT/ OTHER ADULT RESPONSIBLE FOR THE CHILD (FROM COLUMN 1 OF HOUSEHOLD SCHEDULE). RECORD '00' IF NOT LISTED.	LINE NUMBER . . . . . . [ ][ ]	LINE NUMBER . . . . . . [ ][ ]	LINE NUMBER . . . . . . [ ][ ]
210	ASK CONSENT FOR ANEMIA TEST FROM PARENT/OTHER ADULT IDENTIFIED IN 209 AS RESPONSIBLE FOR CHILD.	As part of this survey, we are asking people all over the country to take an anemia test. Anemia is a serious health problem that usually results from poor nutrition, infection, or chronic disease. This survey will assist the government to develop programs to prevent and treat anemia.<br><br>We ask that all children born in 2005 **(9)** or later take part in anemia testing in this survey and give a few drops of blood from a finger or heel. The equipment used to take the blood is clean and completely safe. It has never been used before and will be thrown away after each test.<br><br>The blood will be tested for anemia immediately, and the result will be told to you right away. The result will be kept strictly confidential and will not be shared with anyone other than members of our survey team.<br>Do you have any questions?<br>You can say yes to the test, or you can say no. It is up to you to decide.<br>Will you allow (NAME OF CHILD) to participate in the anemia test?		
211	CIRCLE THE APPROPRIATE CODE AND SIGN YOUR NAME.	GRANTED . . . . 1<br>________ (SIGN)<br>REFUSED 2	GRANTED . . . . 1<br>________ (SIGN)<br>REFUSED 2	GRANTED . . . . 1<br>________ (SIGN)<br>REFUSED 2
212	RECORD HEMOGLOBIN LEVEL HERE AND IN THE ANEMIA PAMPHLET **(11)**.	G/DL [ ][ ] . [ ]<br>NOT PRESENT . . . . . 994<br>REFUSED . . . . 995<br>OTHER 996	G/DL [ ][ ] . [ ]<br>NOT PRESENT . . . . . 994<br>REFUSED . . . . 995<br>OTHER 996	G/DL [ ][ ] . [ ]<br>NOT PRESENT . . . . . 994<br>REFUSED . . . . 995<br>OTHER 996
213	GO BACK TO 203 IN NEXT COLUMN OF THIS QUESTIONNAIRE OR IN THE FIRST COLUMN OF THE NEXT PAGE; IF NO MORE CHILDREN, GO TO 214.			

		CHILD 4	CHILD 5	CHILD 6
202	LINE NUMBER FROM COLUMN 11<br><br>NAME FROM COLUMN 2	LINE NUMBER . . . . .<br><br>NAME ______	LINE NUMBER . . . . .<br><br>NAME ______	LINE NUMBER . . . . .<br><br>NAME ______
203	IF MOTHER INTERVIEWED, COPY MONTH AND YEAR OF BIRTH FROM BIRTH HISTORY AND ASK DAY; IF MOTHER NOT INTERVIEWED, ASK:<br>What is (NAME)'s birth date?	DAY . . . .<br>MONTH . . . . .<br>YEAR	DAY . . . .<br>MONTH . . . . .<br>YEAR	DAY . . . .<br>MONTH . . . . .<br>YEAR
204	CHECK 203:<br>CHILD BORN IN JANUARY 2005 **(9)** OR LATER?	YES . . . . 1<br>NO . . . . 2<br>(GO TO 203 FOR NEXT CHILD OR, IF NO MORE CHILDREN, GO TO 214)	YES . . . . 1<br>NO . . . . 2<br>(GO TO 203 FOR NEXT CHILD OR, IF NO MORE CHILDREN, GO TO 214)	YES . . . . 1<br>NO . . . . 2<br>(GO TO 203 IN FIRST COLUMN OF NEW QUESTIONNAIRE; OR, IF NO MORE CHILDREN, GO TO 214)
205	WEIGHT IN KILOGRAMS<br>**(10)**	KG. __ __ . __ __<br><br>NOT PRESENT . . . . . 9994<br>REFUSED . . . . 9995<br>OTHER 9996	KG. __ __ . __ __<br><br>NOT PRESENT . . . . . 9994<br>REFUSED . . . . 9995<br>OTHER 9996	KG. __ __ . __ __<br><br>NOT PRESENT . . . . . 9994<br>REFUSED . . . . 9995<br>OTHER 9996
206	HEIGHT IN CENTIMETERS	CM. __ __ __ . __<br><br>NOT PRESENT . . . 9994<br>REFUSED . . . . 9995<br>OTHER 9996	CM. __ __ __ . __<br><br>NOT PRESENT . . . 9994<br>REFUSED . . . . 9995<br>OTHER 9996	CM. __ __ __ . __<br><br>NOT PRESENT . . . 9994<br>REFUSED . . . . 9995<br>OTHER 9996
207	MEASURED LYING DOWN OR STANDING UP?	LYING DOWN . . . . 1<br>STANDING UP . . . . 2<br>NOT MEASURED 3	LYING DOWN . . . . 1<br>STANDING UP . . . . 2<br>NOT MEASURED 3	LYING DOWN . . . . 1<br>STANDING UP . . . . 2<br>NOT MEASURED 3
208	CHECK 203:<br>IS CHILD AGE 0-5 MONTHS, I.E., WAS CHILD BORN IN MONTH OF INTERVIEW OR FIVE PREVIOUS MONTHS?	0-5 MONTHS . . . . 1<br>(GO TO 203 FOR NEXT CHILD OR, IF NO MORE CHILDREN, GO TO 214)<br>OLDER 2	0-5 MONTHS . . . . 1<br>(GO TO 203 FOR NEXT CHILD OR, IF NO MORE CHILDREN, GO TO 214)<br>OLDER 2	0-5 MONTHS . . . . 1<br>(GO TO 203 IN FIRST COLUMN OF NEW QUESTIONNAIRE; OR, IF NO MORE CHILDREN, GO TO 214)<br>OLDER 2
209	LINE NUMBER OF PARENT/ OTHER ADULT RESPONSIBLE FOR THE CHILD (FROM COLUMN 1 OF HOUSEHOLD SCHEDULE).<br>RECORD '00' IF NOT LISTED.	LINE NUMBER	LINE NUMBER	LINE NUMBER . . . . .
210	ASK CONSENT FOR ANEMIA TEST FROM PARENT/OTHER ADULT IDENTIFIED IN 209 AS RESPONSIBLE FOR CHILD.	As part of this survey, we are asking people all over the country to take an anemia test. Anemia is a serious health problem that usually results from poor nutrition, infection, or chronic disease. This survey will assist the government to develop programs to prevent and treat anemia.<br><br>We ask that all children born in 2005 **(9)** or later take part in anemia testing in this survey and give a few drops of blood from a finger or heel. The equipment used to take the blood is clean and completely safe. It has never been used before and will be thrown away after each test.<br><br>The blood will be tested for anemia immediately, and the result told to you right away. The result will be kept strictly confidential and will not be shared with anyone other than members of our survey team.<br><br>Do you have any questions?<br>You can say yes to the test, or you can say no. It is up to you to decide.<br>Will you allow (NAME OF CHILD) to participate in the anemia test?		
211	CIRCLE THE APPROPRIATE CODE AND SIGN YOUR NAME.	GRANTED . . . . 1<br>______<br>(SIGN)<br>REFUSED 2	GRANTED . . . . 1<br>______<br>(SIGN)<br>REFUSED 2	GRANTED . . . . 1<br>______<br>(SIGN)<br>REFUSED 2
212	RECORD HEMOGLOBIN LEVEL HERE AND IN THE ANEMIA PAMPHLET **(11)**.	G/DL __ __ . __<br><br>NOT PRESENT . . . . 994<br>REFUSED . . . . 995<br>OTHER 996	G/DL __ __ . __<br><br>NOT PRESENT . . . . 994<br>REFUSED . . . . 995<br>OTHER 996	G/DL __ __ . __<br><br>NOT PRESENT . . . . 994<br>REFUSED . . . . 995<br>OTHER 996
213	GO BACK TO 203 IN NEXT COLUMN OF THIS QUESTIONNAIRE OR IN THE FIRST COLUMN OF AN ADDITIONAL QUESTIONNAIRE; IF NO MORE CHILDREN, GO TO 214.			

WEIGHT, HEIGHT, HEMOGLOBIN MEASUREMENT AND HIV TESTING FOR WOMEN AGE 15-49

214	CHECK COLUMN 9 IN HOUSEHOLD SCHEDULE. RECORD THE LINE NUMBER AND NAME FOR ALL ELIGIBLE WOMEN IN 215.<br>IF THERE ARE MORE THAN THREE WOMEN, USE ADDITIONAL QUESTIONNAIRE(S).			
		WOMAN 1	WOMAN 2	WOMAN 3
215	LINE NUMBER FROM COLUMN 9<br>NAME FROM COLUMN 2	LINE NUMBER . . . . [ ][ ]<br>NAME ______	LINE NUMBER . . . . [ ][ ]<br>NAME ______	LINE NUMBER . . . . [ ][ ]<br>NAME ______
216	WEIGHT IN KILOGRAMS **(10)**	KG. [ ][ ][ ].[ ][ ]<br>NOT PRESENT . . . . 99994<br>REFUSED . . . . 99995<br>OTHER 99996	KG. [ ][ ][ ].[ ][ ]<br>NOT PRESENT . . . . 99994<br>REFUSED . . . . 99995<br>OTHER 99996	KG. [ ][ ][ ].[ ][ ]<br>NOT PRESENT . . . . 99994<br>REFUSED . . . . 99995<br>OTHER 99996
217	HEIGHT IN CENTIMETERS	CM. [ ][ ][ ].[ ]<br>NOT PRESENT . . . . 9994<br>REFUSED . . . . 9995<br>OTHER 9996	CM. [ ][ ][ ].[ ]<br>NOT PRESENT . . . . 9994<br>REFUSED . . . . 9995<br>OTHER 9996	CM. [ ][ ][ ].[ ]<br>NOT PRESENT . . . . 9994<br>REFUSED . . . . 9995<br>OTHER 9996
218	AGE: CHECK COLUMN 7.	15-17 YEARS . . . . 1<br>18-49 YEARS . . . . 2<br>(GO TO 223)	15-17 YEARS . . . . 1<br>18-49 YEARS . . . . 2<br>(GO TO 223)	15-17 YEARS . . . . 1<br>18-49 YEARS . . . . 2<br>(GO TO 223)
219	MARITAL STATUS: CHECK COLUMN 8.	CODE 4 (NEVER IN UNION) . . . . . . 1<br>OTHER . . . . 2<br>(GO TO 223)	CODE 4 (NEVER IN UNION) . . . . . . 1<br>OTHER . . . . 2<br>(GO TO 223)	CODE 4 (NEVER IN UNION) . . . . . . 1<br>OTHER . . . . 2<br>(GO TO 223)
220	RECORD LINE NUMBER OF PARENT/OTHER ADULT RESPON-SIBLE FOR ADOLESCENT. RECORD '00' IF NOT LISTED.	LINE NUMBER OF PARENT OR OTHER RESPONSIBLE ADULT [ ][ ]	LINE NUMBER OF PARENT OR OTHER RESPONSIBLE ADULT [ ][ ]	LINE NUMBER OF PARENT OR OTHER RESPONSIBLE ADULT [ ][ ]
221	ASK CONSENT FOR ANEMIA TEST FROM PARENT/ OTHER ADULT IDENTIFIED IN 220 AS RESPONSIBLE FOR NEVER IN UNION WOMEN AGE 15-17.	As part of this survey, we are asking people all over the country to take an anemia test. Anemia is a serious health problem that usually results from poor nutrition, infection, or chronic disease. This survey will assist the government to develop programs to prevent and treat anemia.<br>For the anemia testing, we will need a few drops of blood from a finger. The equipment used to take the blood is clean and completely safe. It has never been used before and will be thrown away after each test.<br>The blood will be tested for anemia immediately, and the result will be told to you and (NAME OF ADOLESCENT) right away. The result will be kept strictly confidential and will not be shared with anyone other than members of our survey team.<br>Do you have any questions?<br>You can say yes to the test for (NAME OF ADOLESCENT), or you can say no. It is up to you to decide.<br>Will you allow (NAME OF ADOLESCENT) to take the anemia test?		
222	CIRCLE THE APPROPRIATE CODE AND SIGN YOUR NAME.	GRANTED . . . . 1<br>PARENT/OTHER RESPONSIBLE ADULT REFUSED . . . . 2<br>______<br>(SIGN)<br>(IF REFUSED, GO TO 228)	GRANTED . . . . 1<br>PARENT/OTHER RESPONSIBLE ADULT REFUSED . . . . 2<br>______<br>(SIGN)<br>(IF REFUSED, GO TO 228)	GRANTED . . . . 1<br>PARENT/OTHER RESPONSIBLE ADULT REFUSED . . . . 2<br>______<br>(SIGN)<br>(IF REFUSED, GO TO 228)

		WOMAN 1	WOMAN 2	WOMAN 3
	NAME FROM COLUMN 2	NAME ____________	NAME ____________	NAME ____________
223	ASK CONSENT FOR ANEMIA TEST FROM RESPONDENT.	As part of this survey, we are asking people all over the country to take an anemia test. Anemia is a serious health problem that usually results from poor nutrition, infection, or chronic disease. This survey will assist the government to develop programs to prevent and treat anemia.<br><br>For the anemia testing, we will need a few drops of blood from a finger. The equipment used to take the blood is clean and completely safe. It has never been used before and will be thrown away after each test. The blood will be tested for anemia immediately, and the result will be told to you right away. The result will be kept strictly confidential and will not be shared with anyone other than members of our survey team.<br><br>Do you have any questions?<br>You can say yes to the test, or you can say no. It is up to you to decide.<br>Will you take the anemia test?		
224	CIRCLE THE APPROPRIATE CODE AND SIGN YOUR NAME.	GRANTED . . . . 1<br>RESPONDENT REFUSED . . . . . . 2<br>____________<br>(SIGN)<br><br>(IF REFUSED, GO TO 226)	GRANTED . . . . 1<br>RESPONDENT REFUSED . . . . . . 2<br>____________<br>(SIGN)<br><br>(IF REFUSED, GO TO 226)	GRANTED . . . . 1<br>RESPONDENT REFUSED . . . . . . 2<br>____________<br>(SIGN)<br><br>(IF REFUSED, GO TO 226)
225	PREGNANCY STATUS: CHECK 226 IN WOMAN'S QUESTIONNAIRE OR ASK:<br>Are you pregnant?	YES . . . . 1<br>NO . . . . 2<br>DK 8	YES . . . . 1<br>NO . . . . 2<br>DK 8	YES . . . . 1<br>NO . . . . 2<br>DK 8
226<br>**(12)**	AGE: CHECK COLUMN 7.	15-17 YEARS . . . . 1<br>18-49 YEARS . . . . 2<br>(GO TO 230)	15-17 YEARS . . . . 1<br>18-49 YEARS . . . . 2<br>(GO TO 230)	15-17 YEARS . . . . 1<br>18-49 YEARS . . . . 2<br>(GO TO 230)
227<br>**(12)**	MARITAL STATUS: CHECK COLUMN 8.	CODE 4 (NEVER IN UNION) . . . . . . 1<br>OTHER . . . . 2<br>(GO TO 230)	CODE 4 (NEVER IN UNION) . . . . . . 1<br>OTHER . . . . 2<br>(GO TO 230)	CODE 4 (NEVER IN UNION) . . . . . . 1<br>OTHER . . . . 2<br>(GO TO 230)
228<br>**(12)**	ASK CONSENT FOR DBS COLLECTION FROM PARENT/ OTHER ADULT IDENTIFIED IN 220 AS RESPONSIBLE FOR NEVER IN UNION WOMEN AGE 15-17.	As part of the survey we also are asking people all over the country to take an HIV test. HIV is the virus that causes AIDS. AIDS is a very serious illness. The HIV test is being done to see how big the AIDS problem is in (COUNTRY).<br><br>For the HIV test, we need a few (more) drops of blood from a finger. The equipment used to take the blood is clean and completely safe. It has never been used before and will be thrown away after each test. No names will be attached so we will not be able to tell you the test results. No one else will be able to know (NAME OF ADOLESCENT)'s test results either. If (NAME OF ADOLESCENT) wants to know her HIV status, I can provide a list of [nearby] facilities offering counseling and testing for HIV. I will also give her a voucher for free services that can be used at any of these facilities.<br><br>Do you have any questions?<br>You can say yes to the test, or you can say no. It is up to you to decide.<br>Will you allow (NAME OF ADOLESCENT) to take the HIV test?		
229<br>**(12)**	CIRCLE THE APPROPRIATE CODE AND SIGN YOUR NAME.	GRANTED . . . . 1<br>PARENT/OTHER RESPONSIBLE ADULT REFUSED . . . . 2<br>____________<br>(SIGN)<br><br>(IF REFUSED, GO TO 239)	GRANTED . . . . 1<br>PARENT/OTHER RESPONSIBLE ADULT REFUSED . . . . 2<br>____________<br>(SIGN)<br><br>(IF REFUSED, GO TO 239)	GRANTED . . . . 1<br>PARENT/OTHER RESPONSIBLE ADULT REFUSED . . . . 2<br>____________<br>(SIGN)<br><br>(IF REFUSED, GO TO 239)

		WOMAN 1	WOMAN 2	WOMAN 3
	NAME FROM COLUMN 2	NAME ____________	NAME ____________	NAME ____________
230 **(12)**	ASK CONSENT FOR DBS COLLECTION FROM RESPONDENT.	As part of the survey we also are asking people all over the country to take an HIV test. HIV is the virus that causes AIDS. AIDS is a very serious illness. The HIV test is being done to see how big the AIDS problem is in (COUNTRY).<br><br>For the HIV test, we need a few (more) drops of blood from a finger. The equipment used to take the blood is clean and completely safe. It has never been used before and will be thrown away after each test. No names will be attached so we will not be able to tell you the test results. No one else will be able to know your test results either. If you want to know whether you have HIV, I can provide you with a list of [nearby] facilities offering counseling and testing for HIV. I will also give you a voucher for free services for you (and for your partner if you want) that you can use at any of these facilities.<br><br>Do you have any questions?<br>You can say yes to the test, or you can say no. It is up to you to decide.<br>Will you take the HIV test?		
231 **(12)**	CIRCLE THE APPROPRIATE CODE, SIGN YOUR NAME, AND ENTER YOUR INTERVIEWER NUMBER.	GRANTED . . . . 1<br>RESPONDENT REFUSED . . . . . . 2<br>____________<br>(SIGN)<br>[ ][ ][ ]<br>(IF REFUSED, GO TO 239)	GRANTED . . . . 1<br>RESPONDENT REFUSED . . . . . . 2<br>____________<br>(SIGN)<br>[ ][ ][ ]<br>(IF REFUSED, GO TO 239)	GRANTED . . . . 1<br>RESPONDENT REFUSED . . . . . . 2<br>____________<br>(SIGN)<br>[ ][ ][ ]<br>(IF REFUSED, GO TO 239)
232 **(12)**	AGE: CHECK COLUMN 7.	15-17 YEARS . . . . 1<br>18-49 YEARS . . . . 2<br>(GO TO 236)	15-17 YEARS . . . . 1<br>18-49 YEARS . . . . 2<br>(GO TO 236)	15-17 YEARS . . . . 1<br>18-49 YEARS . . . . 2<br>(GO TO 236)
233 **(12)**	MARITAL STATUS: CHECK COLUMN 8.	CODE 4 (NEVER IN UNION) . . . . . . 1<br>OTHER . . . . 2<br>(GO TO 236)	CODE 4 (NEVER IN UNION) . . . . . . 1<br>OTHER . . . . 2<br>(GO TO 236)	CODE 4 (NEVER IN UNION) . . . . . . 1<br>OTHER . . . . 2<br>(GO TO 236)
234 **(12)**	ASK CONSENT FOR ADDITIONAL TESTING FROM PARENT/OTHER ADULT IDENTIFIED IN 220 AS RESPONSIBLE FOR NEVER IN UNION WOMEN AGE 15-17.	We ask you to allow [SURVEY IMPLEMENTING ORGANIZATION/MINISTRY OF HEALTH] to store part of the blood sample at the laboratory for additional tests or research. We are not certain about what additional tests might be done.<br><br>The blood sample will not have any name or other data attached that could identify (NAME OF ADOLESCENT). You do not have to agree. If you do not want the blood sample stored for additional testing (NAME OF ADOLESCENT) can still participate in the HIV testing in this survey. Will you allow us to keep the blood sample stored for additional testing?		
235 **(12)**	CIRCLE THE APPROPRIATE CODE AND SIGN YOUR NAME.	GRANTED . . . . 1<br>PARENT/OTHER RESPONSIBLE ADULT REFUSED . . . . 2<br>____________<br>(SIGN)<br>(IF REFUSED, GO TO 238)	GRANTED . . . . 1<br>PARENT/OTHER RESPONSIBLE ADULT REFUSED . . . . 2<br>____________<br>(SIGN)<br>(IF REFUSED, GO TO 238)	GRANTED . . . . 1<br>PARENT/OTHER RESPONSIBLE ADULT REFUSED . . . . 2<br>____________<br>(SIGN)<br>(IF REFUSED, GO TO 238)
236 **(12)**	ASK CONSENT FOR ADDITIONAL TESTING FROM RESPONDENT.	We ask you to allow [SURVEY IMPLEMENTING ORGANIZATION/MINISTRY OF HEALTH] to store part of the blood sample at the laboratory for additional tests or research. We are not certain about what additional tests might be done.<br><br>The blood sample will not have any name or other data attached that could identify you. You do not have to agree. If you do not want the blood sample stored for additional testing, you can still participate in the HIV testing in this survey. Will you allow us to keep the blood sample stored for additional testing?		

		WOMAN 1	WOMAN 2	WOMAN 3
	NAME FROM COLUMN 2	NAME ____________	NAME ____________	NAME ____________
237 **(12)**	CIRCLE THE APPROPRIATE CODE AND SIGN YOUR NAME.	GRANTED . . . . 1<br>RESPONDENT REFUSED . . . . . . 2<br>____________<br>(SIGN)<br>(IF GRANTED, GO TO 239)	GRANTED . . . . 1<br>RESPONDENT REFUSED . . . . . . 2<br>____________<br>(SIGN)<br>(IF GRANTED, GO TO 239)	GRANTED . . . . 1<br>RESPONDENT REFUSED . . . . . . 2<br>____________<br>(SIGN)<br>(IF GRANTED, GO TO 239)
238 **(12)**	ADDITIONAL TESTS	CHECK 235 AND 237:<br>IF CONSENT HAS NOT BEEN GRANTED WRITE "NO ADDITIONAL TEST" ON THE FILTER PAPER.	CHECK 235 AND 237:<br>IF CONSENT HAS NOT BEEN GRANTED WRITE "NO ADDITIONAL TEST" ON THE FILTER PAPER.	CHECK 235 AND 237:<br>IF CONSENT HAS NOT BEEN GRANTED WRITE "NO ADDITIONAL TEST" ON THE FILTER PAPER.
239 **(12)**	PREPARE EQUIPMENT AND SUPPLIES ONLY FOR THE TEST(S) FOR WHICH CONSENT HAS BEEN OBTAINED AND PROCEED WITH THE TEST(S).			
240	RECORD HEMO-GLOBIN LEVEL HERE AND IN ANEMIA PAMPHLET **(11).**	G/DL . . . . [ ][ ].[ ]<br>NOT PRESENT . . . . 994<br>REFUSED . . . . 995<br>OTHER 996	G/DL . . . . [ ][ ].[ ]<br>NOT PRESENT . . . . 994<br>REFUSED . . . . 995<br>OTHER 996	G/DL . . . . [ ][ ].[ ]<br>NOT PRESENT . . . . 994<br>REFUSED . . . . 995<br>OTHER 996
241 **(12)**	BAR CODE LABEL	PUT THE 1ST BAR CODE LABEL HERE.<br>NOT PRESENT . . . . 99994<br>REFUSED . . . . 99995<br>OTHER . . . . 99996<br>PUT THE 2ND BAR CODE LABEL ON THE RESPONDENT'S FILTER PAPER AND THE 3RD ON THE TRANSMITTAL FORM.	PUT THE 1ST BAR CODE LABEL HERE.<br>NOT PRESENT . . . . 99994<br>REFUSED . . . . 99995<br>OTHER . . . . 99996<br>PUT THE 2ND BAR CODE LABEL ON THE RESPONDENT'S FILTER PAPER AND THE 3RD ON THE TRANSMITTAL FORM.	PUT THE 1ST BAR CODE LABEL HERE.<br>NOT PRESENT . . . . 99994<br>REFUSED . . . . 99995<br>OTHER . . . . 99996<br>PUT THE 2ND BAR CODE LABEL ON THE RESPONDENT'S FILTER PAPER AND THE 3RD ON THE TRANSMITTAL FORM.
242	GO BACK TO 216 IN NEXT COLUMN OF THIS QUESTIONNAIRE OR IN THE FIRST COLUMN OF AN ADDITIONAL QUESTIONNAIRE; IF NO MORE WOMEN, GO TO 243.			

WEIGHT, HEIGHT, HEMOGLOBIN MEASUREMENT AND HIV TESTING FOR MEN AGE 15-49

243	CHECK COLUMN 10 IN HOUSEHOLD SCHEDULE. RECORD THE LINE NUMBER AND NAME FOR ALL ELIGIBLE MEN IN 244.<br>IF THERE ARE MORE THAN THREE MEN, USE ADDITIONAL QUESTIONNAIRE(S).			
		MAN 1	MAN 2	MAN 3
244	LINE NUMBER FROM COLUMN 10<br>NAME FROM COLUMN 2	LINE NUMBER . . . . [ ][ ]<br>NAME ____________	LINE NUMBER . . . . [ ][ ]<br>NAME ____________	LINE NUMBER . . . . [ ][ ]<br>NAME ____________
245	WEIGHT IN KILOGRAMS **(10)**	KG. [ ][ ][ ].[ ][ ]<br>NOT PRESENT . . . . 99994<br>REFUSED . . . . 99995<br>OTHER 99996	KG. [ ][ ][ ].[ ][ ]<br>NOT PRESENT . . . . 99994<br>REFUSED . . . . 99995<br>OTHER 99996	KG. [ ][ ][ ].[ ][ ]<br>NOT PRESENT . . . . 99994<br>REFUSED . . . . 99995<br>OTHER 99996
246	HEIGHT IN CENTIMETERS	CM. . . . . [ ][ ][ ].[ ]<br>NOT PRESENT . . . . 9994<br>REFUSED . . . . 9995<br>OTHER 9996	CM. . . . . [ ][ ][ ].[ ]<br>NOT PRESENT . . . . 9994<br>REFUSED . . . . 9995<br>OTHER 9996	CM. . . . . . . [ ][ ][ ].[ ]<br>NOT PRESENT . . . . 9994<br>REFUSED . . . . 9995<br>OTHER 9996
247	AGE: CHECK COLUMN 7.	15-17 YEARS . . . . 1<br>18-49 YEARS . . . . 2<br>(GO TO 252)	15-17 YEARS . . . . 1<br>18-49 YEARS . . . . 2<br>(GO TO 252)	15-17 YEARS . . . . 1<br>18-49 YEARS . . . . 2<br>(GO TO 252)
248	MARITAL STATUS: CHECK COLUMN 8.	CODE 4 (NEVER IN UNION) . . . . . . 1<br>OTHER . . . . 2<br>(GO TO 252)	CODE 4 (NEVER IN UNION) . . . . . . 1<br>OTHER . . . . 2<br>(GO TO 252)	CODE 4 (NEVER IN UNION) . . . . . . 1<br>OTHER . . . . 2<br>(GO TO 252)
249	RECORD LINE NUMBER OF PARENT/OTHER ADULT RESPON-SIBLE FOR ADOLESCENT. RECORD '00' IF NOT LISTED.	LINE NUMBER OF PARENT OR OTHER RESPONSIBLE ADULT [ ][ ]	LINE NUMBER OF PARENT OR OTHER RESPONSIBLE ADULT [ ][ ]	LINE NUMBER OF PARENT OR OTHER RESPONSIBLE ADULT [ ][ ]
250	ASK CONSENT FOR ANEMIA TEST FROM PARENT/ OTHER ADULT IDENTIFIED IN 249 AS RESPONSIBLE FOR NEVER IN UNION MEN AGE 15-17.	As part of this survey, we are asking people all over the country to take an anemia test. Anemia is a serious health problem that usually results from poor nutrition, infection, or chronic disease. This survey will assist the government to develop programs to prevent and treat anemia.<br>For the anemia testing, we will need a few drops of blood from a finger. The equipment used to take the blood is clean and completely safe. It has never been used before and will be thrown away after each test.<br>The blood will be tested for anemia immediately, and the result will be told to you and (NAME OF ADOLESCENT) right away. The result will be kept strictly confidential and will not be shared with anyone other than members of our survey team.<br>Do you have any questions?<br>You can say yes to the test for (NAME OF ADOLESCENT), or you can say no. It is up to you to decide.<br>Will you allow (NAME OF ADOLESCENT) to take the anemia test?		
251	CIRCLE THE APPROPRIATE CODE AND SIGN YOUR NAME.	GRANTED . . . . 1<br>PARENT/OTHER RESPONSIBLE ADULT REFUSED . . . . 2<br>____________<br>(SIGN)<br>(IF REFUSED, GO TO 256)	GRANTED . . . . 1<br>PARENT/OTHER RESPONSIBLE ADULT REFUSED . . . . 2<br>____________<br>(SIGN)<br>(IF REFUSED, GO TO 256)	GRANTED . . . . 1<br>PARENT/OTHER RESPONSIBLE ADULT REFUSED . . . . 2<br>____________<br>(SIGN)<br>(IF REFUSED, GO TO 256)

		MAN 1	MAN 2	MAN 3
	NAME FROM COLUMN 2	NAME ______	NAME ______	NAME ______
252	ASK CONSENT FOR ANEMIA TEST FROM RESPONDENT.	As part of this survey, we are asking people all over the country to take an anemia test. Anemia is a serious health problem that usually results from poor nutrition, infection, or chronic disease. This survey will assist the government to develop programs to prevent and treat anemia.<br><br>For the anemia testing, we will need a few drops of blood from a finger. The equipment used to take the blood is clean and completely safe. It has never been used before and will be thrown away after each test. The blood will be tested for anemia immediately, and the result will be told to you right away. The result will be kept strictly confidential and will not be shared with anyone other than members of our survey team.<br><br>Do you have any questions?<br>You can say yes to the test, or you can say no. It is up to you to decide.<br>Will you take the anemia test?		
253	CIRCLE THE APPROPRIATE CODE AND SIGN YOUR NAME.	GRANTED . . . . 1<br>RESPONDENT REFUSED . . . . . . 2<br>______<br>(SIGN)	GRANTED . . . . 1<br>RESPONDENT REFUSED . . . . . . 2<br>______<br>(SIGN)	GRANTED . . . . 1<br>RESPONDENT REFUSED . . . . . . 2<br>______<br>(SIGN)
254 **(12)**	AGE: CHECK COLUMN 7.	15-17 YEARS . . . . 1<br>18-49 YEARS . . . . 2<br>(GO TO 258)	15-17 YEARS . . . . 1<br>18-49 YEARS . . . . 2<br>(GO TO 258)	15-17 YEARS . . . . 1<br>18-49 YEARS . . . . 2<br>(GO TO 258)
255 **(12)**	MARITAL STATUS: CHECK COLUMN 8.	CODE 4 (NEVER IN UNION) . . . . . . 1<br>OTHER . . . . 2<br>(GO TO 258)	CODE 4 (NEVER IN UNION) . . . . . . 1<br>OTHER . . . . 2<br>(GO TO 258)	CODE 4 (NEVER IN UNION) . . . . . . 1<br>OTHER . . . . 2<br>(GO TO 258)
256 **(12)**	ASK CONSENT FOR DBS COLLECTION FROM PARENT/ OTHER ADULT IDENTIFIED IN 249 AS RESPONSIBLE FOR NEVER IN UNION MEN AGE 15-17.	As part of the survey we also are asking people all over the country to take an HIV test. HIV is the virus that causes AIDS. AIDS is a very serious illness. The HIV test is being done to see how big the AIDS problem is in (COUNTRY).<br><br>For the HIV test, we need a few (more) drops of blood from a finger. The equipment used to take the blood is clean and completely safe. It has never been used before and will be thrown away after each test. No names will be attached so we will not be able to tell you the test results. No one else will be able to know (NAME OF ADOLESCENT)'s test results either. If (NAME OF ADOLESCENT) wants to know his HIV status, I can provide him with a list of [nearby] facilities offering counseling and testing for HIV. I will also give him a voucher for free services that can be used at any of these facilities.<br><br>Do you have any questions?<br>You can say yes to the test for (NAME OF ADOLESCENT), or you can say no. It is up to you to decide.<br>Will you allow (NAME OF ADOLESCENT) to take the HIV test?		
257 **(12)**	CIRCLE THE APPROPRIATE CODE AND SIGN YOUR NAME.	GRANTED . . . . 1<br>PARENT/OTHER RESPONSIBLE ADULT REFUSED . . . . 2<br>______<br>(SIGN)<br>(IF REFUSED, GO TO 267)	GRANTED . . . . 1<br>PARENT/OTHER RESPONSIBLE ADULT REFUSED . . . . 2<br>______<br>(SIGN)<br>(IF REFUSED, GO TO 267)	GRANTED . . . . 1<br>PARENT/OTHER RESPONSIBLE ADULT REFUSED . . . . 2<br>______<br>(SIGN)<br>(IF REFUSED, GO TO 267)

		MAN 1	MAN 2	MAN 3
	NAME FROM COLUMN 2	NAME ______	NAME ______	NAME ______
258 **(12)**	ASK CONSENT FOR DBS COLLECTION FROM RESPONDENT	As part of the survey we also are asking people all over the country to take an HIV test. HIV is the virus that causes AIDS. AIDS is a very serious illness. The HIV test is being done to see how big the AIDS problem is in (COUNTRY).<br><br>For the HIV test, we need a few more drops of blood from a finger. The equipment used in taking the blood is clean and completely safe. It has never been used before and will be thrown away after each test. No names will be attached so we will not be able to tell you the test results. No one else will be able to know your test results either. If you want to know whether you have HIV, I can provide you with a list of [nearby] facilities offering counseling and testing for HIV. I will also give you a voucher for free services for you (and for your partner if you want) that you can use at any of these facilities.<br><br>Do you have any questions?<br>You can say yes to the test, or you can say no. It is up to you to decide.<br>Will you take the HIV test?		
259 **(12)**	CIRCLE THE APPROPRIATE CODE, SIGN YOUR NAME, AND ENTER YOUR INTERVIEWER NUMBER.	GRANTED . . . . 1<br>RESPONDENT REFUSED . . . . . . 2<br>______<br>(SIGN)<br>[ ][ ][ ]<br>(IF REFUSED, GO TO 267)	GRANTED . . . . 1<br>RESPONDENT REFUSED . . . . . . 2<br>______<br>(SIGN)<br>[ ][ ][ ]<br>(IF REFUSED, GO TO 267)	GRANTED . . . . 1<br>RESPONDENT REFUSED . . . . . . 2<br>______<br>(SIGN)<br>[ ][ ][ ]<br>(IF REFUSED, GO TO 267)
260 **(12)**	AGE: CHECK COLUMN 7.	15-17 YEARS . . . . 1<br>18-49 YEARS . . . . 2<br>(GO TO 264)	15-17 YEARS . . . . 1<br>18-49 YEARS . . . . 2<br>(GO TO 264)	15-17 YEARS . . . . 1<br>18-49 YEARS . . . . 2<br>(GO TO 264)
261 **(12)**	MARITAL STATUS: CHECK COLUMN 8.	CODE 4 (NEVER IN UNION) . . . . . . 1<br>OTHER . . . . 2<br>(GO TO 264)	CODE 4 (NEVER IN UNION) . . . . . . 1<br>OTHER . . . . 2<br>(GO TO 264)	CODE 4 (NEVER IN UNION) . . . . . . 1<br>OTHER . . . . 2<br>(GO TO 264)
262 **(12)**	ASK CONSENT FOR ADDITIONAL TESTING FROM PARENT/OTHER ADULT IDENTIFIED IN 249 AS RESPONSIBLE FOR NEVER IN UNION MEN AGE 15-17.	We ask you to allow [SURVEY IMPLEMENTING ORGANIZATION/MINISTRY OF HEALTH] to store part of the blood sample at the laboratory for additional tests or research. We are not certain about what additional tests might be done.<br><br>The blood sample will not have any name or other data attached that could identify (NAME OF ADOLESCENT). You do not have to agree. If you do not want the blood sample stored for additional testing, (NAME OF ADOLESCENT) can still participate in the HIV testing in this survey. Will you allow us to keep the blood sample stored for additional testing?		
263 **(12)**	CIRCLE THE APPROPRIATE CODE AND SIGN YOUR NAME.	GRANTED . . . . 1<br>PARENT/OTHER RESPONSIBLE ADULT REFUSED . . . . 2<br>______<br>(SIGN)<br>(IF REFUSED, GO TO 266)	GRANTED . . . . 1<br>PARENT/OTHER RESPONSIBLE ADULT REFUSED . . . . 2<br>______<br>(SIGN)<br>(IF REFUSED, GO TO 266)	GRANTED . . . . 1<br>PARENT/OTHER RESPONSIBLE ADULT REFUSED . . . . 2<br>______<br>(SIGN)<br>(IF REFUSED, GO TO 266)

		MAN 1	MAN 2	MAN 3
	NAME FROM COLUMN 2	NAME ____________	NAME ____________	NAME ____________
264 **(12)**	ASK CONSENT FOR ADDITIONAL TESTING FROM RESPONDENT.	We ask you to allow [SURVEY IMPLEMENTING ORGANIZATION/MINISTRY OF HEALTH] to store part of the blood sample at the laboratory for additional tests or research. We are not certain about what additional tests might be done.<br><br>The blood sample will not have any name or other data attached that could identify you. You do not have to agree. If you do not want the blood sample stored for additional testing, you can still participate in the HIV testing in this survey. Will you allow us to keep the blood sample stored for additional testing?		
265 **(12)**	CIRCLE THE APPROPRIATE CODE AND SIGN YOUR NAME.	GRANTED . . . . 1<br>RESPONDENT REFUSED . . . . . . 2<br>____________<br>(SIGN)<br>(IF GRANTED, GO TO 267)	GRANTED . . . . 1<br>RESPONDENT REFUSED . . . . . . 2<br>____________<br>(SIGN)<br>(IF GRANTED, GO TO 267)	GRANTED . . . . 1<br>RESPONDENT REFUSED . . . . . . 2<br>____________<br>(SIGN)<br>(IF GRANTED, GO TO 267)
266 **(12)**	ADDITIONAL TESTS	CHECK 263 AND 265:<br>IF CONSENT HAS NOT BEEN GRANTED WRITE "NO ADDITIONAL TEST" ON THE FILTER PAPER.	CHECK 263 AND 265:<br>IF CONSENT HAS NOT BEEN GRANTED WRITE "NO ADDITIONAL TEST" ON THE FILTER PAPER.	CHECK 263 AND 265:<br>IF CONSENT HAS NOT BEEN GRANTED WRITE "NO ADDITIONAL TEST" ON THE FILTER PAPER.
267 **(12)**	PREPARE EQUIPMENT AND SUPPLIES ONLY FOR THE TEST(S) FOR WHICH CONSENT HAS BEEN OBTAINED AND PROCEED WITH THE TEST(S).			
268	RECORD HEMO-GLOBIN LEVEL HERE AND IN ANEMIA PAMPHLET **(11)**.	G/DL . . . . [ ][ ].[ ]<br>NOT PRESENT . . . . 994<br>REFUSED . . . . 995<br>OTHER 996	G/DL . . . . [ ][ ].[ ]<br>NOT PRESENT . . . . 994<br>REFUSED . . . . 995<br>OTHER 996	G/DL . . . . [ ][ ].[ ]<br>NOT PRESENT . . . . 994<br>REFUSED . . . . 995<br>OTHER 996
269 **(12)**	BAR CODE LABEL	PUT THE 1ST BAR CODE LABEL HERE.<br>NOT PRESENT . . . . .99994<br>REFUSED . . . . 99995<br>OTHER . . . . 99996<br>PUT THE 2ND BAR CODE LABEL ON THE RESPONDENT'S FILTER PAPER AND THE 3RD ON THE TRANSMITTAL FORM.	PUT THE 1ST BAR CODE LABEL HERE.<br>NOT PRESENT . . . . .99994<br>REFUSED . . . . 99995<br>OTHER . . . . 99996<br>PUT THE 2ND BAR CODE LABEL ON THE RESPONDENT'S FILTER PAPER AND THE 3RD ON THE TRANSMITTAL FORM.	PUT THE 1ST BAR CODE LABEL HERE.<br>NOT PRESENT . . . . .99994<br>REFUSED . . . . 99995<br>OTHER . . . . 99996<br>PUT THE 2ND BAR CODE LABEL ON THE RESPONDENT'S FILTER PAPER AND THE 3RD ON THE TRANSMITTAL FORM.
270	GO BACK TO 245 IN NEXT COLUMN OF THIS QUESTIONNAIRE OR IN THE FIRST COLUMN OF AN ADDITIONAL QUESTIONNAIRE; IF NO MORE MEN, END INTERVIEW.			

FOOTNOTES

(1) This section should be adapted for country-specific survey design.
(2) In Q. 18, the year should refer to the school year that is in session at the time the survey begins. If the survey begins between two school years, then the year should refer to the school year that just ended.
(3) Coding categories to be developed locally and revised based on the pretest; however, the broad categories must be maintained.
(4) Each country should add to the list at least five items of furniture (such as a table, a chair, a sofa, a bed, an armoire, or a cupboard or cabinet). In addition, each country should add at least four additional household appliances so that the list includes at least three items that even a poor household may have, at least three items that a middle income household may have, and at least three items that a high income household may have. Some possible additions are clock, water pump, grain grinder, fan, blender, water heater, generator, washing machine, microwave oven, computer, VCR or DVD player, cassette or CD player, camera, air conditioner or cooler, color TV, sewing machine.
(5) Add other country-specific animals, such as oxen, water buffalo, camels, llamas, alpacas, pigs, ducks, geese, or elephants.
(6) The question should be deleted in countries that do not have an organized spraying program to prevent the transmission of malaria.
(7) The question should be deleted in countries that are not affected by malaria.
(8) There are many different kinds of iodine testing kits available. The proper test kit should be selected in each country depending on the type of iodine additive used in the country (potassium iodate or potassium iodide). If both of these additives are used in a country, then both types of test kits should be used.
(9) Year of fieldwork is assumed to be 2010. For fieldwork beginning in 2011 or 2012, the year should be 2006 or 2007, respectively.
(10) In countries where the weighing scale shows the weight to only one decimal place, retain only one box after the decimal point and delete the first '9' from the other three codes.
(11) In countries where some enumeration areas are higher than 1,000 meters, altitude information should be collected on a separate form for each enumeration area higher than 1,000 meters so that the anemia estimates can be adjusted appropriately.
(12) Questions should be omitted in countries in which HIV testing is not a component of the survey.

[THIS PAGE IS INTENTIONALLY BLANK]

DEMOGRAPHIC AND HEALTH SURVEYS
MODEL WOMAN'S QUESTIONNAIRE

[NAME OF COUNTRY]
[NAME OF ORGANIZATION]

**IDENTIFICATION (1)**	
PLACE NAME ______	
NAME OF HOUSEHOLD HEAD ______	
CLUSTER NUMBER	☐☐☐
HOUSEHOLD NUMBER	☐☐
NAME AND LINE NUMBER OF WOMAN ______	☐☐

**INTERVIEWER VISITS**				
	1	2	3	FINAL VISIT
DATE	______	______	______	DAY ☐☐<br>MONTH ☐☐<br>YEAR ☐☐☐☐
INTERVIEWER'S NAME	______	______	______	INT. NUMBER ☐☐☐
RESULT*	______	______	______	RESULT ☐
NEXT VISIT: DATE	______	______		TOTAL NUMBER OF VISITS ☐
TIME	______	______		

*RESULT CODES:

1	COMPLETED	4	REFUSED		
2	NOT AT HOME	5	PARTLY COMPLETED	7	OTHER ______
3	POSTPONED	6	INCAPACITATED		(SPECIFY)

COUNTRY-SPECIFIC INFORMATION: LANGUAGE OF QUESTIONNAIRE, LANGUAGE OF INTERVIEW, NATIVE LANGUAGE OF RESPONDENT, AND WHETHER TRANSLATOR USED

SUPERVISOR	FIELD EDITOR	OFFICE EDITOR	KEYED BY
NAME ______ ☐☐☐	NAME ______ ☐☐☐	☐☐	☐☐

(1) This section should be adapted for country-specific survey design.

Note: Questions with blue highlighting in the question number column are HIV related questions that may be deleted in some circumstances (see footnotes). Questions with pink highlighting in the question number column are malaria related questions that may be deleted in some circumstances (see footnotes). Questions with yellow highlighting in the question number column are other questions that may be deleted in some circumstances (see footnotes).

SECTION 1. RESPONDENT'S BACKGROUND

INTRODUCTION AND CONSENT

INFORMED CONSENT

Hello. My name is _______________________________________. I am working with (NAME OF ORGANIZATION). We are conducting a survey about health all over (NAME OF COUNTRY). The information we collect will help the government to plan health services. Your household was selected for the survey. The questions usually take about 30 to 60 minutes. All of the answers you give will be confidential and will not be shared with anyone other than members of our survey team. You don't have to be in the survey, but we hope you will agree to answer the questions since your views are important. If I ask you any question you don't want to answer, just let me know and I will go on to the next question or you can stop the interview at any time.

In case you need more information about the survey, you may contact the person listed on the card that has already been given to your household.

Do you have any questions? May I begin the interview now?

SIGNATURE OF INTERVIEWER: _______________________________ DATE: ____________________

RESPONDENT AGREES TO BE INTERVIEWED . . . 1 ↓ RESPONDENT DOES NOT AGREE TO BE INTERVIEWED . . . 2 → END

NO.	QUESTIONS AND FILTERS	CODING CATEGORIES	SKIP
101	RECORD THE TIME.	HOUR . . . . [ ][ ]<br>MINUTES . . . . [ ][ ]	
102	In what month and year were you born?	MONTH . . . . [ ][ ]<br>DON'T KNOW MONTH . . . . 98<br>YEAR . . . . [ ][ ][ ][ ]<br>DON'T KNOW YEAR 9998	
103	How old were you at your last birthday?<br><br>COMPARE AND CORRECT 102 AND/OR 103 IF INCONSISTENT.	AGE IN COMPLETED YEARS [ ][ ]	
104	Have you ever attended school?	YES . . . . 1<br>NO 2	 → 108
105	What is the highest level of school you attended: primary, secondary, or higher? (1)	PRIMARY . . . . 1<br>SECONDARY . . . . 2<br>HIGHER 3	
106	What is the highest (grade/form/year) you completed at that level? (1)<br><br>IF COMPLETED LESS THAN ONE YEAR AT THAT LEVEL, RECORD '00'.	GRADE/FORM/YEAR . . . . [ ][ ]	
107	CHECK 105:<br>PRIMARY [ ] ↓ SECONDARY OR HIGHER [ ] →		110

NO.	QUESTIONS AND FILTERS	CODING CATEGORIES	SKIP
108	Now I would like you to read this sentence to me.<br><br>SHOW CARD TO RESPONDENT. **(2)**<br><br>IF RESPONDENT CANNOT READ WHOLE SENTENCE, PROBE:<br>Can you read any part of the sentence to me?	CANNOT READ AT ALL . . . . 1<br>ABLE TO READ ONLY PARTS OF SENTENCE . . . . 2<br>ABLE TO READ WHOLE SENTENCE 3<br>NO CARD WITH REQUIRED LANGUAGE ________________ 4<br>(SPECIFY LANGUAGE)<br>BLIND/VISUALLY IMPAIRED 5	
109	CHECK 108:<br>CODE '2', '3' OR '4' CIRCLED ☐ ↓ CODE '1' OR '5' CIRCLED ☐ →		→ 111
110	Do you read a newspaper or magazine at least once a week, less than once a week or not at all?	AT LEAST ONCE A WEEK . . . . 1<br>LESS THAN ONCE A WEEK . . . . 2<br>NOT AT ALL 3	
111	Do you listen to the radio at least once a week, less than once a week or not at all?	AT LEAST ONCE A WEEK . . . . 1<br>LESS THAN ONCE A WEEK . . . . 2<br>NOT AT ALL 3	
112	Do you watch television at least once a week, less than once a week or not at all?	AT LEAST ONCE A WEEK . . . . 1<br>LESS THAN ONCE A WEEK . . . . 2<br>NOT AT ALL 3	
113	COUNTRY-SPECIFIC QUESTION ON RELIGION, IF APPROPRIATE.		
114	COUNTRY-SPECIFIC QUESTION ON ETHNICITY, IF APPROPRIATE.		
115<br>**(3)**	In the last 12 months, how many times have you been away from home for one or more nights?	NUMBER OF TIMES . . . . ☐☐<br>NONE 00	 → 201
116<br>**(3)**	In the last 12 months, have you been away from home for more than one month at a time?	YES . . . . 1<br>NO 2	

(1) Revise according to the local education system.
(2) Each card should have four simple sentences appropriate to the country (e.g., "Parents love their children.", "Farming is hard work.", "The child is reading a book.", "Children work hard at school."). Cards should be prepared for every language in which respondents are likely to be literate.
(3) The question may be considered for deletion in countries with a very low HIV prevalence.

NO.	QUESTIONS AND FILTERS	CODING CATEGORIES	SKIP
201	Now I would like to ask about all the births you have had during your life. Have you ever given birth?	YES . . . . . 1<br>NO 2	 → 206
202	Do you have any sons or daughters to whom you have given birth who are now living with you?	YES . . . . . 1<br>NO 2	 → 204
203	How many sons live with you?<br><br>And how many daughters live with you?<br><br>IF NONE, RECORD '00'.	SONS AT HOME . . . . . [ ][ ]<br><br>DAUGHTERS AT HOME . . . . . [ ][ ]	
204	Do you have any sons or daughters to whom you have given birth who are alive but do not live with you?	YES . . . . . 1<br>NO 2	 → 206
205	How many sons are alive but do not live with you?<br><br>And how many daughters are alive but do not live with you?<br><br>IF NONE, RECORD '00'.	SONS ELSEWHERE . . . . . [ ][ ]<br><br>DAUGHTERS ELSEWHERE [ ][ ]	
206	Have you ever given birth to a boy or girl who was born alive but later died?<br><br>IF NO, PROBE: Any baby who cried or showed signs of life but did not survive?	YES . . . . . 1<br>NO 2	 → 208
207	How many boys have died?<br><br>And how many girls have died?<br><br>IF NONE, RECORD '00'.	BOYS DEAD . . . . . [ ][ ]<br><br>GIRLS DEAD . . . . . [ ][ ]	
208	SUM ANSWERS TO 203, 205, AND 207, AND ENTER TOTAL.<br>IF NONE, RECORD '00'.	TOTAL BIRTHS . . . . . [ ][ ]	
209	CHECK 208:<br><br>Just to make sure that I have this right: you have had in TOTAL _____ births during your life. Is that correct?<br><br>YES [ ] ↓ NO [ ] → PROBE AND CORRECT 201-208 AS NECESSARY.		
210	CHECK 208:<br><br>ONE OR MORE BIRTHS [ ] ↓ NO BIRTHS [ ] →		226

211 Now I would like to record the names of all your births, whether still alive or not, starting with the first one you had.
RECORD NAMES OF ALL THE BIRTHS IN 212. RECORD TWINS AND TRIPLETS ON SEPARATE ROWS.
(IF THERE ARE MORE THAN 12 BIRTHS, USE AN ADDITIONAL QUESTIONNAIRE, STARTING WITH THE SECOND ROW).

212	213	214	215	216	217 IF ALIVE:	218 IF ALIVE:	219 IF ALIVE:	220 IF DEAD:	221
What name was given to your (first/next) baby?<br>RECORD NAME.<br>BIRTH HISTORY NUMBER	Is (NAME) a boy or a girl?	Were any of these births twins?	In what month and year was (NAME) born?<br>PROBE: When is his/her birthday?	Is (NAME) still alive?	How old was (NAME) at his/her last birthday?<br>RECORD AGE IN COM-PLETED YEARS.	Is (NAME) living with you?	RECORD HOUSE-HOLD LINE NUMBER OF CHILD (RECORD '00' IF CHILD NOT LISTED IN HOUSE-HOLD).	How old was (NAME) when he/she died?<br>IF '1 YR', PROBE: How many months old was (NAME)? RECORD DAYS IF LESS THAN 1 MONTH; MONTHS IF LESS THAN TWO YEARS; OR YEARS.	Were there any other live births between (NAME OF PREVIOUS BIRTH) and (NAME), including any children who died after birth?
01	BOY 1<br>GIRL 2	SING 1<br>MULT 2	MONTH<br>YEAR	YES . . 1<br>NO . . . 2 → 220	AGE IN YEARS	YES . . . 1<br>NO 2	HOUSEHOLD LINE NUMBER<br>(NEXT BIRTH)	DAYS . . . 1<br>MONTHS 2<br>YEARS . . 3	
02	BOY 1<br>GIRL 2	SING 1<br>MULT 2	MONTH<br>YEAR	YES . . 1<br>NO . . . 2 → 220	AGE IN YEARS	YES . . . 1<br>NO 2	HOUSEHOLD LINE NUMBER<br>(GO TO 221)	DAYS . . . 1<br>MONTHS 2<br>YEARS . . 3	YES . . . . 1 ADD BIRTH<br>NO . . . . . 2 NEXT BIRTH
03	BOY 1<br>GIRL 2	SING 1<br>MULT 2	MONTH<br>YEAR	YES . . 1<br>NO . . . 2 → 220	AGE IN YEARS	YES . . . 1<br>NO 2	HOUSEHOLD LINE NUMBER<br>(GO TO 221)	DAYS . . . 1<br>MONTHS 2<br>YEARS . . 3	YES . . . . 1 ADD BIRTH<br>NO . . . . . 2 NEXT BIRTH
04	BOY 1<br>GIRL 2	SING 1<br>MULT 2	MONTH<br>YEAR	YES . . 1<br>NO . . . 2 → 220	AGE IN YEARS	YES . . . 1<br>NO 2	HOUSEHOLD LINE NUMBER<br>(GO TO 221)	DAYS . . . 1<br>MONTHS 2<br>YEARS . . 3	YES . . . . 1 ADD BIRTH<br>NO . . . . . 2 NEXT BIRTH
05	BOY 1<br>GIRL 2	SING 1<br>MULT 2	MONTH<br>YEAR	YES . . 1<br>NO . . . 2 → 220	AGE IN YEARS	YES . . . 1<br>NO 2	HOUSEHOLD LINE NUMBER<br>(GO TO 221)	DAYS . . . 1<br>MONTHS 2<br>YEARS . . 3	YES . . . . 1 ADD BIRTH<br>NO . . . . . 2 NEXT BIRTH
06	BOY 1<br>GIRL 2	SING 1<br>MULT 2	MONTH<br>YEAR	YES . . 1<br>NO . . . 2 → 220	AGE IN YEARS	YES . . . 1<br>NO 2	HOUSEHOLD LINE NUMBER<br>(GO TO 221)	DAYS . . . 1<br>MONTHS 2<br>YEARS . . 3	YES . . . . 1 ADD BIRTH<br>NO . . . . . 2 NEXT BIRTH
07	BOY 1<br>GIRL 2	SING 1<br>MULT 2	MONTH<br>YEAR	YES . . 1<br>NO . . . 2 → 220	AGE IN YEARS	YES . . . 1<br>NO 2	HOUSEHOLD LINE NUMBER<br>(GO TO 221)	DAYS . . . 1<br>MONTHS 2<br>YEARS . . 3	YES . . . . 1 ADD BIRTH<br>NO . . . . . 2 NEXT BIRTH

212	213	214	215	216	217 IF ALIVE:	218 IF ALIVE:	219 IF ALIVE:	220 IF DEAD:	221
What name was given to your next baby?<br><br>RECORD NAME.<br><br>BIRTH HISTORY NUMBER	Is (NAME) a boy or a girl?	Were any of these births twins?	In what month and year was (NAME) born?<br><br>PROBE: When is his/her birthday?	Is (NAME) still alive?	How old was (NAME) at his/her last birthday?<br><br>RECORD AGE IN COM-PLETED YEARS.	Is (NAME) living with you?	RECORD HOUSE-HOLD LINE NUMBER OF CHILD (RECORD '00' IF CHILD NOT LISTED IN HOUSE-HOLD).	How old was (NAME) when he/she died?<br><br>IF '1 YR', PROBE: How many months old was (NAME)? RECORD DAYS IF LESS THAN 1 MONTH; MONTHS IF LESS THAN TWO YEARS; OR YEARS.	Were there any other live births between (NAME OF PREVIOUS BIRTH) and (NAME), including any children who died after birth?
08	BOY 1<br>GIRL 2	SING 1<br>MULT 2	MONTH<br>YEAR	YES . . 1<br>NO . . . 2 → 220	AGE IN YEARS	YES . . . 1<br>NO 2	HOUSEHOLD LINE NUMBER<br>(GO TO 221)	DAYS . . . 1<br>MONTHS 2<br>YEARS . . 3	YES . . . . 1 ADD BIRTH<br>NO . . . . . 2 NEXT BIRTH
09	BOY 1<br>GIRL 2	SING 1<br>MULT 2	MONTH<br>YEAR	YES . . 1<br>NO . . . 2 → 220	AGE IN YEARS	YES . . . 1<br>NO 2	HOUSEHOLD LINE NUMBER<br>(GO TO 221)	DAYS . . . 1<br>MONTHS 2<br>YEARS . . 3	YES . . . . 1 ADD BIRTH<br>NO . . . . . 2 NEXT BIRTH
10	BOY 1<br>GIRL 2	SING 1<br>MULT 2	MONTH<br>YEAR	YES . . 1<br>NO . . . 2 → 220	AGE IN YEARS	YES . . . 1<br>NO 2	HOUSEHOLD LINE NUMBER<br>(GO TO 221)	DAYS . . . 1<br>MONTHS 2<br>YEARS . . 3	YES . . . . 1 ADD BIRTH<br>NO . . . . . 2 NEXT BIRTH
11	BOY 1<br>GIRL 2	SING 1<br>MULT 2	MONTH<br>YEAR	YES . . 1<br>NO . . . 2 → 220	AGE IN YEARS	YES . . . 1<br>NO 2	HOUSEHOLD LINE NUMBER<br>(GO TO 221)	DAYS . . . 1<br>MONTHS 2<br>YEARS . . 3	YES . . . . 1 ADD BIRTH<br>NO . . . . . 2 NEXT BIRTH
12	BOY 1<br>GIRL 2	SING 1<br>MULT 2	MONTH<br>YEAR	YES . . 1<br>NO . . . 2 → 220	AGE IN YEARS	YES . . . 1<br>NO 2	HOUSEHOLD LINE NUMBER<br>(GO TO 221)	DAYS . . . 1<br>MONTHS 2<br>YEARS . . 3	YES . . . . 1 ADD BIRTH<br>NO . . . . . 2 NEXT BIRTH

222	Have you had any live births since the birth of (NAME OF LAST BIRTH)? IF YES, RECORD BIRTH(S) IN TABLE.	YES . . . . 1<br>NO 2	
223	COMPARE 208 WITH NUMBER OF BIRTHS IN HISTORY ABOVE AND MARK:<br>NUMBERS ARE SAME ☐ NUMBERS ARE DIFFERENT ☐ → (PROBE AND RECONCILE)		
224	CHECK 215:<br>ENTER THE NUMBER OF BIRTHS IN 2005 **(1)** OR LATER.	NUMBER OF BIRTHS . . . . ☐<br>NONE 0	→ 226

NO.	QUESTIONS AND FILTERS	CODING CATEGORIES	SKIP
225	C FOR EACH BIRTH SINCE JANUARY 2005 **(1)**, ENTER 'B' IN THE MONTH OF BIRTH IN THE CALENDAR. WRITE THE NAME OF THE CHILD TO THE LEFT OF THE 'B' CODE. FOR EACH BIRTH, ASK THE NUMBER OF MONTHS THE PREGNANCY LASTED AND RECORD 'P' IN EACH OF THE PRECEDING MONTHS ACCORDING TO THE DURATION OF PREGNANCY. (NOTE: THE NUMBER OF 'P's MUST BE ONE LESS THAN THE NUMBER OF MONTHS THAT THE PREGNANCY LASTED.)		
226	Are you pregnant now?	YES . . . . . 1<br>NO . . . . . 2<br>UNSURE 8	 2, 8 → 230
227	How many months pregnant are you?<br><br>RECORD NUMBER OF COMPLETED MONTHS.<br><br>C ENTER 'P's IN THE CALENDAR, BEGINNING WITH THE MONTH OF INTERVIEW AND FOR THE TOTAL NUMBER OF COMPLETED MONTHS.	MONTHS . . . . . [ ][ ]	
228	When you got pregnant, did you want to get pregnant at that time?	YES . . . . . 1<br>NO 2	1 → 230
229	Did you want to have a baby later on or did you not want any (more) children?	LATER . . . . . 1<br>NO MORE 2	
230	Have you ever had a pregnancy that miscarried, was aborted, or ended in a stillbirth?	YES . . . . . 1<br>NO 2	 2 → 238
231	When did the last such pregnancy end?	MONTH . . . . . [ ][ ]<br>YEAR . . . . . [ ][ ][ ][ ]	
232	CHECK 231:<br>LAST PREGNANCY ENDED IN JAN. 2005 **(1)** OR LATER [ ] ↓<br>LAST PREGNANCY ENDED BEFORE JAN. 2005 **(1)** [ ]		→ 238
233	How many months pregnant were you when the last such pregnancy ended?<br><br>C RECORD NUMBER OF COMPLETED MONTHS. ENTER 'T' IN THE CALENDAR IN THE MONTH THAT THE PREGNANCY TERMINATED AND 'P' FOR THE REMAINING NUMBER OF COMPLETED MONTHS.	MONTHS . . . . . [ ][ ]	
234	Since January 2005 **(1)**, have you had any other pregnancies that did not result in a live birth?	YES . . . . . 1<br>NO 2	 2 → 236
235	ASK THE DATE AND THE DURATION OF PREGNANCY FOR EACH EARLIER NON-LIVE BIRTH PREGNANCY BACK TO JANUARY 2005. **(1)**<br><br>C ENTER 'T' IN THE CALENDAR IN THE MONTH THAT EACH PREGNANCY TERMINATED AND 'P' FOR THE REMAINING NUMBER OF COMPLETED MONTHS.		
236	Did you have any miscarriages, abortions or stillbirths that ended before 2005 **(1)**?	YES . . . . . 1<br>NO 2	 2 → 238
237	When did the last such pregnancy that terminated before 2005 **(1)** end?	MONTH . . . . . [ ][ ]<br>YEAR . . . . . [ ][ ][ ][ ]	

NO.	QUESTIONS AND FILTERS	CODING CATEGORIES	SKIP
238	When did your last menstrual period start?<br><br>____________________<br>(DATE, IF GIVEN)	DAYS AGO . . . . 1 [ ][ ]<br>WEEKS AGO . . . . 2 [ ][ ]<br>MONTHS AGO . . . . 3 [ ][ ]<br>YEARS AGO . . . . 4 [ ][ ]<br><br>IN MENOPAUSE/<br>HAS HAD HYSTERECTOMY . . . 994<br><br>BEFORE LAST BIRTH . . . . 995<br><br>NEVER MENSTRUATED 996	
239	From one menstrual period to the next, are there certain days when a woman is more likely to become pregnant?	YES . . . . 1<br>NO . . . . 2<br>DON'T KNOW 8	 2, 8 → 301
240	Is this time just before her period begins, during her period, right after her period has ended, or halfway between two periods?	JUST BEFORE HER PERIOD<br>BEGINS . . . . 1<br>DURING HER PERIOD . . . . 2<br>RIGHT AFTER HER<br>PERIOD HAS ENDED . . . . 3<br>HALFWAY BETWEEN<br>TWO PERIODS . . . . 4<br><br>OTHER __________________ 6<br>(SPECIFY)<br>DON'T KNOW 8	

(1) Year of fieldwork is assumed to be 2010. For fieldwork beginning in 2011 or 2012, the year should be 2006 or 2007, respectively.

SECTION 3. CONTRACEPTION

301	Now I would like to talk about family planning - the various ways or methods that a couple can use to delay or avoid a pregnancy.<br><br>Have you ever heard of (METHOD)? **(1)**		
01	**Female Sterilization**. PROBE: Women can have an operation to avoid having any more children.	YES . . . . . . 1<br>NO 2	
02	**Male Sterilization**. PROBE: Men can have an operation to avoid having any more children.	YES . . . . . . 1<br>NO 2	
03	**IUD**. PROBE: Women can have a loop or coil placed inside them by a doctor or a nurse.	YES . . . . . . 1<br>NO 2	
04	**Injectables**. PROBE: Women can have an injection by a health provider that stops them from becoming pregnant for one or more months.	YES . . . . . . 1<br>NO 2	
05	**Implants**. PROBE: Women can have one or more small rods placed in their upper arm by a doctor or nurse which can prevent pregnancy for one or more years.	YES . . . . . . 1<br>NO 2	
06	**Pill**. PROBE: Women can take a pill every day to avoid becoming pregnant.	YES . . . . . . 1<br>NO 2	
07	**Condom**. PROBE: Men can put a rubber sheath on their penis before sexual intercourse.	YES . . . . . . 1<br>NO 2	
08	**Female Condom**. PROBE: Women can place a sheath in their vagina before sexual intercourse.	YES . . . . . . 1<br>NO 2	
09<br>**(2)**	**Lactational Amenorrhea Method (LAM)**. **(2)**	YES . . . . . . 1<br>NO 2	
10	**Rhythm Method**. PROBE: To avoid pregnancy, women do not have sexual intercourse on the days of the month they think they can get pregnant.	YES . . . . . . 1<br>NO 2	
11	**Withdrawal**. PROBE: Men can be careful and pull out before climax.	YES . . . . . . 1<br>NO 2	
12	**Emergency Contraception**. PROBE: As an emergency measure, within three days after they have unprotected sexual intercourse, women can take special pills to prevent pregnancy. **(3)**	YES . . . . . . 1<br>NO 2	
13	Have you heard of any other ways or methods that women or men can use to avoid pregnancy?	YES . . . . . . 1<br>__________<br>(SPECIFY)<br>__________<br>(SPECIFY)<br>NO 2	
302	CHECK 226:<br>NOT PREGNANT OR UNSURE ☐ ↓<br>PREGNANT ☐ → 311		311
303	Are you currently doing something or using any method to delay or avoid getting pregnant?	YES . . . . . . 1<br>NO 2	→ 311

NO.	QUESTIONS AND FILTERS	CODING CATEGORIES		SKIP
304	Which method are you using? **(4)**<br><br>CIRCLE ALL MENTIONED.<br><br>IF MORE THAN ONE METHOD MENTIONED, FOLLOW SKIP INSTRUCTION FOR HIGHEST METHOD IN LIST.	FEMALE STERILIZATION	A	→ 307
		MALE STERILIZATION	B	→ 307
		IUD	C	→ 308A
		INJECTABLES	D	→ 308A
		IMPLANTS	E	→ 308A
		PILL	F	
		CONDOM	G	→ 306
		FEMALE CONDOM	H	→ 308A
		DIAPHRAGM	I	→ 308A
		FOAM/JELLY	J	→ 308A
		LACTATIONAL AMEN. METHOD	K	→ 308A
		RHYTHM METHOD	L	→ 308A
		WITHDRAWAL	M	→ 308A
		OTHER MODERN METHOD	X	→ 308A
		OTHER TRADITIONAL METHOD . . .	Y	→ 308A
305	What is the brand name of the pills you are using?<br><br>IF DON'T KNOW THE BRAND,<br>ASK TO SEE THE PACKAGE.	BRAND A	01	→ 308A
		BRAND B	02	→ 308A
		BRAND C	03	→ 308A
		OTHER ______________________<br>(SPECIFY)	96	→ 308A
		DON'T KNOW	98	→ 308A
306	What is the brand name of the condoms you are using?<br><br>IF DON'T KNOW THE BRAND,<br>ASK TO SEE THE PACKAGE.	BRAND A	01	→ 308A
		BRAND B	02	→ 308A
		BRAND C	03	→ 308A
		OTHER ______________________<br>(SPECIFY)	96	→ 308A
		DON'T KNOW	98	→ 308A
307	In what facility did the sterilization take place? **(5)**<br><br>PROBE TO IDENTIFY THE TYPE OF SOURCE.<br><br>IF UNABLE TO DETERMINE IF PUBLIC OR PRIVATE SECTOR, WRITE THE NAME OF THE PLACE.<br><br>______________________________<br>(NAME OF PLACE)	PUBLIC SECTOR		
		GOVT. HOSPITAL	11	
		GOVT. HEALTH CENTER	12	
		FAMILY PLANNING CLINIC	13	
		MOBILE CLINIC	14	
		OTHER PUBLIC SECTOR ______________________<br>(SPECIFY)	16	
		PRIVATE MEDICAL SECTOR		
		PRIVATE HOSPITAL/CLINIC	21	
		PRIVATE DOCTOR'S OFFICE	23	
		MOBILE CLINIC	24	
		OTHER PRIVATE MEDICAL SECTOR ______________________<br>(SPECIFY)	26	
		OTHER ______________________<br>(SPECIFY)	96	
		DON'T KNOW	98	

NO.	QUESTIONS AND FILTERS	CODING CATEGORIES	SKIP
308	In what month and year was the sterilization performed?		
308A	Since what month and year have you been using (CURRENT METHOD) without stopping?<br><br>PROBE: For how long have you been using (CURRENT METHOD) now without stopping?	MONTH . . . . [ ][ ]<br>YEAR . . . . [ ][ ][ ][ ]	
309	CHECK 308/308A, 215 AND 231:<br><br>ANY BIRTH OR PREGNANCY TERMINATION AFTER MONTH AND YEAR OF START OF USE OF CONTRACEPTION IN 308/308A — YES [ ] / NO [ ]<br><br>GO BACK TO 308/308A, PROBE AND RECORD MONTH AND YEAR AT START OF CONTINUOUS USE OF CURRENT METHOD (MUST BE AFTER LAST BIRTH OR PREGNANCY TERMINATION).		
310	CHECK 308/308A:<br><br>YEAR IS 2005 **(6)** OR LATER [ ]<br>**C** ENTER CODE FOR METHOD USED IN MONTH OF INTERVIEW IN THE CALENDAR AND IN EACH MONTH BACK TO THE DATE STARTED USING.<br><br>YEAR IS 2004 **(7)** OR EARLIER [ ]<br>**C** ENTER CODE FOR METHOD USED IN MONTH OF INTERVIEW IN THE CALENDAR AND EACH MONTH BACK TO JANUARY 2005 **(6)**.<br>THEN SKIP TO →		322
311	I would like to ask you some questions about the times you or your partner may have used a method to avoid getting pregnant during the last few years.<br><br>USE CALENDAR TO PROBE FOR EARLIER PERIODS OF USE AND NONUSE, STARTING WITH MOST RECENT USE, BACK TO JANUARY 2005. **(6)**<br>USE NAMES OF CHILDREN, DATES OF BIRTH, AND PERIODS OF PREGNANCY AS REFERENCE POINTS.<br><br>**C** **IN COLUMN 1**, ENTER METHOD USE CODE OR '0' FOR NONUSE IN EACH BLANK MONTH.<br><br>ILLUSTRATIVE QUESTIONS:<br>* When was the last time you used a method? Which method was that?<br>* When did you start using that method? How long after the birth of (NAME)?<br>* How long did you use the method then?<br><br>**IN COLUMN 2**, ENTER CODES FOR DISCONTINUATION NEXT TO THE LAST MONTH OF USE. NUMBER OF CODES IN COLUMN 2 MUST BE SAME AS NUMBER OF INTERRUPTIONS OF METHOD USE IN COLUMN 1.<br><br>ASK WHY SHE STOPPED USING THE METHOD. IF A PREGNANCY FOLLOWED, ASK WHETHER SHE BECAME PREGNANT UNINTENTIONALLY WHILE USING THE METHOD OR DELIBERATELY STOPPED TO GET PREGNANT.<br><br>ILLUSTRATIVE QUESTIONS:<br>* Why did you stop using the (METHOD)? Did you become pregnant while using (METHOD), or did you stop to get pregnant, or did you stop for some other reason?<br>* IF DELIBERATELY STOPPED TO BECOME PREGNANT, ASK: How many months did it take you to get pregnant after you stopped using (METHOD)? AND ENTER '0' IN EACH SUCH MONTH IN COLUMN 1.		

NO.	QUESTIONS AND FILTERS	CODING CATEGORIES	SKIP
312	CHECK THE CALENDAR FOR USE OF ANY CONTRACEPTIVE METHOD IN ANY MONTH<br><br>NO METHOD USED ☐ ↓<br>ANY METHOD USED ☐		ANY METHOD USED → 314
313	Have you ever used anything or tried in any way to delay or avoid getting pregnant?	YES . . . . . 1<br>NO 2	YES/NO → 324
314	CHECK 304:<br><br>CIRCLE METHOD CODE:<br><br>IF MORE THAN ONE METHOD CODE CIRCLED IN 304, CIRCLE CODE FOR HIGHEST METHOD IN LIST.	NO CODE CIRCLED . . . . . 00<br>FEMALE STERILIZATION . . . . . 01<br>MALE STERILIZATION . . . . . 02<br>IUD . . . . . 03<br>INJECTABLES . . . . . 04<br>IMPLANTS . . . . . 05<br>PILL . . . . . 06<br>CONDOM . . . . . 07<br>FEMALE CONDOM . . . . . 08<br>DIAPHRAGM . . . . . 09<br>FOAM/JELLY . . . . . 10<br>LACTATIONAL AMEN. METHOD . . . . . 11<br>RHYTHM METHOD . . . . . 12<br>WITHDRAWAL . . . . . 13<br>OTHER MODERN METHOD . . . . . 95<br>OTHER TRADITIONAL METHOD 96	00 → 324<br>01 → 317A<br>02 → 326<br>11, 12 → 315A<br>13, 95, 96 → 326
315<br><br>315A	You first started using (CURRENT METHOD) in (DATE FROM 308/308A). Where did you get it at that time? **(5)**<br><br>Where did you learn how to use the rhythm/lactational amenorrhea method?<br><br>PROBE TO IDENTIFY THE TYPE OF SOURCE.<br><br>IF UNABLE TO DETERMINE IF PUBLIC OR PRIVATE SECTOR, WRITE THE NAME OF THE PLACE.<br><br>____________________<br>(NAME OF PLACE)	PUBLIC SECTOR<br>GOVT. HOSPITAL . . . . . 11<br>GOVT. HEALTH CENTER . . . . . 12<br>FAMILY PLANNING CLINIC . . . . . 13<br>MOBILE CLINIC . . . . . 14<br>FIELDWORKER . . . . . 15<br>OTHER PUBLIC SECTOR ______ 16<br>(SPECIFY)<br><br>PRIVATE MEDICAL SECTOR<br>PRIVATE HOSPITAL/CLINIC . . . . . 21<br>PHARMACY . . . . . 22<br>PRIVATE DOCTOR . . . . . 23<br>MOBILE CLINIC . . . . . 24<br>FIELDWORKER . . . . . 25<br>OTHER PRIVATE MEDICAL SECTOR ______ 26<br>(SPECIFY)<br><br>OTHER SOURCE<br>SHOP . . . . . 31<br>CHURCH . . . . . 32<br>FRIEND/RELATIVE . . . . . 33<br><br>OTHER ______ 96<br>(SPECIFY)	

NO.	QUESTIONS AND FILTERS	CODING CATEGORIES	SKIP
316	CHECK 304:<br><br>CIRCLE METHOD CODE:<br><br>IF MORE THAN ONE METHOD CODE CIRCLED IN 304, CIRCLE CODE FOR HIGHEST METHOD IN LIST.	IUD . . . . 03<br>INJECTABLES . . . . 04<br>IMPLANTS . . . . 05<br>PILL . . . . 06<br>CONDOM . . . . 07<br>FEMALE CONDOM . . . . 08<br>DIAPHRAGM . . . . 09<br>FOAM/JELLY . . . . 10<br>LACTATIONAL AMEN. METHOD . . . . . 11<br>RHYTHM METHOD 12	 <br><br><br>→ 323<br><br>(08–10) → 320<br><br>→ 326<br>→ 326
317<br><br><br><br>317A	At that time, were you told about side effects or problems you might have with the method?<br><br>When you got sterilized, were you told about side effects or problems you might have with the method?	YES . . . . 1<br>NO 2	→ 319
318	Were you ever told by a health or family planning worker about side effects or problems you might have with the method?	YES . . . . 1<br>NO 2	 → 320
319	Were you told what to do if you experienced side effects or problems?	YES . . . . 1<br>NO 2	
320	CHECK 317:<br><br>CODE '1' CIRCLED ↓ At that time, were you told about other methods of family planning that you could use?<br><br>CODE '1' NOT CIRCLED ↓ When you obtained (CURRENT METHOD FROM 314) from (SOURCE OF METHOD FROM 307 OR 315), were you told about other methods of family planning that you could use?	YES . . . . 1<br>NO 2	→ 322
321	Were you ever told by a health or family planning worker about other methods of family planning that you could use?	YES . . . . 1<br>NO 2	
322	CHECK 304:<br><br>CIRCLE METHOD CODE:<br><br>IF MORE THAN ONE METHOD CODE CIRCLED IN 304, CIRCLE CODE FOR HIGHEST METHOD IN LIST.	FEMALE STERILIZATION . . . . 01<br>MALE STERILIZATION . . . . 02<br>IUD . . . . 03<br>INJECTABLES . . . . 04<br>IMPLANTS . . . . 05<br>PILL . . . . 06<br>CONDOM . . . . 07<br>FEMALE CONDOM . . . . 08<br>DIAPHRAGM . . . . 09<br>FOAM/JELLY . . . . 10<br>LACTATIONAL AMEN. METHOD . . . . . 11<br>RHYTHM METHOD . . . . 12<br>WITHDRAWAL . . . . 13<br>OTHER MODERN METHOD . . . . 95<br>OTHER TRADITIONAL METHOD . . . 96	(01–02) → 326<br><br><br><br><br><br><br><br><br><br><br><br>(11–96) → 326

NO.	QUESTIONS AND FILTERS	CODING CATEGORIES	SKIP
323	Where did you obtain (CURRENT METHOD) the last time? **(5)**<br><br>PROBE TO IDENTIFY THE TYPE OF SOURCE.<br><br>IF UNABLE TO DETERMINE IF PUBLIC OR PRIVATE SECTOR, WRITE THE NAME OF THE PLACE.<br><br>____________________<br>(NAME OF PLACE)	PUBLIC SECTOR<br>GOVT. HOSPITAL . . . . 11<br>GOVT. HEALTH CENTER . . . . 12<br>FAMILY PLANNING CLINIC . . . . 13<br>MOBILE CLINIC . . . . 14<br>FIELDWORKER . . . . 15<br>OTHER PUBLIC<br>SECTOR __________ 16<br>(SPECIFY)<br><br>PRIVATE MEDICAL SECTOR<br>PRIVATE HOSPITAL/CLINIC . . . . . 21<br>PHARMACY . . . . 22<br>PRIVATE DOCTOR . . . . 23<br>MOBILE CLINIC . . . . 24<br>FIELDWORKER . . . . 25<br>OTHER PRIVATE MEDICAL<br>SECTOR __________ 26<br>(SPECIFY)<br><br>OTHER SOURCE<br>SHOP . . . . 31<br>CHURCH . . . . 32<br>FRIEND/RELATIVE . . . . 33<br><br>OTHER __________ 96<br>(SPECIFY)	→ 326
324	Do you know of a place where you can obtain a method of family planning?	YES . . . . 1<br>NO 2	 → 326
325	Where is that? **(5)**<br><br>Any other place?<br><br>PROBE TO IDENTIFY EACH TYPE OF SOURCE.<br><br>IF UNABLE TO DETERMINE IF PUBLIC OR PRIVATE SECTOR, WRITE THE NAME OF THE PLACE.<br><br>____________________<br>(NAME OF PLACE(S))	PUBLIC SECTOR<br>GOVT. HOSPITAL . . . . A<br>GOVT. HEALTH CENTER . . . . B<br>FAMILY PLANNING CLINIC . . . . C<br>MOBILE CLINIC . . . . D<br>FIELDWORKER . . . . E<br>OTHER PUBLIC<br>SECTOR __________ F<br>(SPECIFY)<br><br>PRIVATE MEDICAL SECTOR<br>PRIVATE HOSPITAL/CLINIC . . . . . G<br>PHARMACY . . . . H<br>PRIVATE DOCTOR . . . . I<br>MOBILE CLINIC . . . . J<br>FIELDWORKER . . . . K<br>OTHER PRIVATE MEDICAL<br>SECTOR __________ L<br>(SPECIFY)<br><br>OTHER SOURCE<br>SHOP . . . . M<br>CHURCH . . . . N<br>FRIEND/RELATIVE . . . . O<br><br>OTHER __________ X<br>(SPECIFY)	

NO.	QUESTIONS AND FILTERS	CODING CATEGORIES	SKIP
326	In the last 12 months, were you visited by a fieldworker who talked to you about family planning? **(8)**	YES . . . . 1<br>NO 2	
327	In the last 12 months, have you visited a health facility for care for yourself (or your children)?	YES . . . . 1<br>NO 2	 → 401
328	Did any staff member at the health facility speak to you about family planning methods?	YES . . . . 1<br>NO 2	

(1) If Standard Days Method is commonly used, it may be added to the table before Lactational Amenorrhea. "**Standard Days Method** (use local term, such as CycleBeads™ , as appropriate) PROBE: A woman uses a string of colored beads to know the days she can get pregnant. On the days she can get pregnant, she uses a condom or does not have sexual intercourse." If Standard Days Method is added to Q. 301, it should also be added before LAM to Qs. 304, 314, 316, 322, and Column 1 of the calendar.

(2) The LAM method should be deleted in countries that do not have a LAM program. In these countries, LAM should also be deleted as a coding category in Qs. 304, 314, 316, 322, and Column 1 of the calendar. A description of LAM should not be provided in Q. 301.

(3) Studies have indicated emergency contraception can be effective up to five days. Verify country program recommendations and modify wording if appropriate.

(4) Other commonly used methods may be added to the list, such as contraceptive patch, contraceptive vaginal ring, or sponge. Any codes added in Q. 304 must also be added to Qs. 314, 316, 322, and Column 1 of the calendar. These methods should not be added to Q. 301.

(5) Coding categories to be developed locally and revised based on the pretest; however, the broad categories must be maintained.

(6) Year of fieldwork is assumed to be 2010. For fieldwork beginning in 2011 or 2012, the year should be 2006 or 2007, respectively.

(7) Year of fieldwork is assumed to be 2010. For fieldwork beginning in 2011 or 2012, the year should be 2005 or 2006, respectively.

(8) In countries without national fieldworker programs that include family planning, Q. 326 should be deleted.

SECTION 4. PREGNANCY AND POSTNATAL CARE

401	CHECK 224: ONE OR MORE BIRTHS IN 2005 **(1)** OR LATER ↓ / NO BIRTHS IN 2005 **(1)** OR LATER → 556				
402	CHECK 215: ENTER IN THE TABLE THE BIRTH HISTORY NUMBER, NAME, AND SURVIVAL STATUS OF EACH BIRTH IN 2005 **(1)** OR LATER. ASK THE QUESTIONS ABOUT ALL OF THESE BIRTHS. BEGIN WITH THE LAST BIRTH. (IF THERE ARE MORE THAN 3 BIRTHS, USE LAST 2 COLUMNS OF ADDITIONAL QUESTIONNAIRES).<br><br>Now I would like to ask some questions about your children born in the last five years. (We will talk about each separately.)				
403	BIRTH HISTORY NUMBER FROM 212 IN BIRTH HISTORY	LAST BIRTH<br>BIRTH HISTORY NUMBER ☐☐	NEXT-TO-LAST BIRTH<br>BIRTH HISTORY NUMBER ☐☐	SECOND-FROM-LAST BIRTH<br>BIRTH HISTORY NUMBER ☐☐	
404	FROM 212 AND 216	NAME ________<br>LIVING ☐ DEAD ☐	NAME ________<br>LIVING ☐ DEAD ☐	NAME ________<br>LIVING ☐ DEAD ☐	
405	When you got pregnant with (NAME), did you want to get pregnant at that time?	YES . . . . 1<br>(SKIP TO 408)<br>NO 2	YES . . . . 1<br>(SKIP TO 430)<br>NO 2	YES . . . . 1<br>(SKIP TO 430)<br>NO 2	
406	Did you want to have a baby later on, or did you not want any (more) children?	LATER . . . . 1<br>NO MORE . . . . 2<br>(SKIP TO 408)	LATER . . . . 1<br>NO MORE . . . . 2<br>(SKIP TO 430)	LATER . . . . 1<br>NO MORE . . . . 2<br>(SKIP TO 430)	
407	How much longer did you want to wait?	MONTHS . . 1 ☐☐<br>YEARS . . 2 ☐☐<br>DON'T KNOW . . . 998	MONTHS . . 1 ☐☐<br>YEARS . . 2 ☐☐<br>DON'T KNOW . . . 998	MONTHS . . 1 ☐☐<br>YEARS . . 2 ☐☐<br>DON'T KNOW . . . 998	
408	Did you see anyone for antenatal care for this pregnancy?	YES . . . . 1<br>NO . . . . 2<br>(SKIP TO 415)			
409	Whom did you see? **(2)**<br><br>Anyone else?<br><br>PROBE TO IDENTIFY EACH TYPE OF PERSON AND RECORD ALL MENTIONED.	HEALTH PERSONNEL<br>DOCTOR . . . . A<br>NURSE/MIDWIFE B<br>AUXILIARY MIDWIFE . . . . . C<br>OTHER PERSON<br>TRADITIONAL BIRTH ATTENDANT D<br>COMMUNITY/ VILLAGE HEALTH WORKER . . . E<br><br>OTHER ________ X<br>(SPECIFY)			

NO.	QUESTIONS AND FILTERS	LAST BIRTH<br>NAME ________	NEXT-TO-LAST BIRTH<br>NAME ________	SECOND-FROM-LAST BIRTH<br>NAME ________
410	Where did you receive antenatal care for this pregnancy? **(2)**<br><br>Anywhere else?<br><br>PROBE TO IDENTIFY EACH TYPE OF SOURCE.<br><br>IF UNABLE TO DETERMINE IF PUBLIC OR PRIVATE SECTOR, WRITE THE NAME OF THE PLACE.<br><br>________<br>(NAME OF PLACE(S))	HOME<br>YOUR HOME . . . A<br>OTHER HOME . . . B<br><br>PUBLIC SECTOR<br>GOVT. HOSPITAL C<br>GOVT. HEALTH CENTER . . . . . D<br>GOVT. HEALTH POST . . . . E<br>OTHER PUBLIC SECTOR<br>________ F<br>(SPECIFY)<br>PRIVATE MED. SECTOR<br>PVT. HOSPITAL/ CLINIC . . . . G<br>OTHER PRIVATE MED. SECTOR<br>________ H<br>(SPECIFY)<br><br>OTHER________ X<br>(SPECIFY)		
411	How many months pregnant were you when you first received antenatal care for this pregnancy?	MONTHS . . . [ ][ ]<br><br>DON'T KNOW 98		
412	How many times did you receive antenatal care during this pregnancy?	NUMBER OF TIMES [ ][ ]<br><br>DON'T KNOW 98		
413	As part of your antenatal care during this pregnancy, were any of the following done at least once:<br><br>Was your blood pressure measured?<br>Did you give a urine sample?<br>Did you give a blood sample?	YES NO<br><br>BP . . . . 1 2<br>URINE . . . . . 1 2<br>BLOOD . . . 1 2		
414	During (any of) your antenatal care visit(s), were you told about things to look out for that might suggest problems with the pregnancy?	YES . . . . 1<br>NO . . . . 2<br>DON'T KNOW 8		
415	During this pregnancy, were you given an injection in the arm to prevent the baby from getting tetanus, that is, convulsions after birth? **(3)**	YES . . . . 1<br><br>NO . . . . 2<br>(SKIP TO 418) ←<br>DON'T KNOW 8		

NO.	QUESTIONS AND FILTERS	LAST BIRTH<br>NAME ________________	NEXT-TO-LAST BIRTH<br>NAME ________________	SECOND-FROM-LAST BIRTH<br>NAME ________________
416	During this pregnancy, how many times did you get a tetanus injection?	TIMES . . . . [ ]<br>DON'T KNOW 8		
417	CHECK 416:	2 OR MORE TIMES [ ] (SKIP TO 421)<br>OTHER [ ]		
418	At any time before this pregnancy, did you receive any tetanus injections?	YES . . . . 1<br>NO . . . . 2<br>(SKIP TO 421)<br>DON'T KNOW . . . 8		
419	Before this pregnancy, how many times did you receive a tetanus injection?<br><br>IF 7 OR MORE TIMES, RECORD '7'.	TIMES . . . . [ ]<br>DON'T KNOW 8		
420	How many years ago did you receive the last tetanus injection before this pregnancy?	YEARS AGO . . . . . [ ][ ]		
421	During this pregnancy, were you given or did you buy any iron tablets or iron syrup? **(4)**<br><br>SHOW TABLETS/SYRUP. **(4)**	YES . . . . 1<br>NO . . . . 2<br>(SKIP TO 423)<br>DON'T KNOW 8		
422	During the whole pregnancy, for how many days did you take the tablets or syrup? **(4,5)**<br><br>IF ANSWER IS NOT NUMERIC, PROBE FOR APPROXIMATE NUMBER OF DAYS.	DAYS [ ][ ][ ]<br>DON'T KNOW . . . 998		
423	During this pregnancy, did you take any drug for intestinal worms?	YES . . . . 1<br>NO . . . . 2<br>DON'T KNOW 8		
424<br>**(6)**	During this pregnancy, did you take any drugs to keep you from getting malaria?	YES . . . . 1<br>NO . . . . 2<br>(SKIP TO 430)<br>DON'T KNOW 8		
425<br>**(6)**	What drugs did you take?<br><br>RECORD ALL MENTIONED.<br>IF TYPE OF DRUG IS NOT DETERMINED, SHOW TYPICAL ANTIMALARIAL DRUGS TO RESPONDENT.	SP/FANSIDAR . . . . . A<br>CHLOROQUINE . . . B<br>OTHER ____________ X<br>(SPECIFY)<br>DON'T KNOW . . . . Z		

NO.	QUESTIONS AND FILTERS	LAST BIRTH<br>NAME ________	NEXT-TO-LAST BIRTH<br>NAME ________	SECOND-FROM-LAST BIRTH<br>NAME ________
426<br>**(6)**	CHECK 425:<br><br>SP/FANSIDAR TAKEN FOR MALARIA PREVENTION.	CODE 'A' CIRCLED ☐<br>CODE 'A' NOT CIRCLED ☐<br>(SKIP TO 430)		
427<br>**(6)**	How many times did you take (SP/Fansidar) during this pregnancy?	TIMES . . . . . ☐☐		
428<br>**(6)**	CHECK 409:<br><br>ANTENATAL CARE FROM HEALTH PERSONNEL DURING THIS PREGNANCY	CODE 'A', 'B' OR 'C' CIRCLED ☐<br>OTHER ☐<br>(SKIP TO 430)		
429<br>**(6)**	Did you get the (SP/Fansidar) during any antenatal care visit, during another visit to a health facility or from another source?	ANTENATAL VISIT . . 1<br>ANOTHER FACILITY VISIT . . . . 2<br>OTHER SOURCE 6		
430	When (NAME) was born, was he/she very large, larger than average, average, smaller than average, or very small?	VERY LARGE . . . . . 1<br>LARGER THAN AVERAGE . . . . . 2<br>AVERAGE . . . . 3<br>SMALLER THAN AVERAGE . . . . . 4<br>VERY SMALL . . . . . 5<br>DON'T KNOW 8	VERY LARGE . . . . . 1<br>LARGER THAN AVERAGE . . . . . 2<br>AVERAGE . . . . 3<br>SMALLER THAN AVERAGE . . . . . 4<br>VERY SMALL . . . . . 5<br>DON'T KNOW 8	VERY LARGE . . . . . 1<br>LARGER THAN AVERAGE . . . . . 2<br>AVERAGE . . . . 3<br>SMALLER THAN AVERAGE . . . . . 4<br>VERY SMALL . . . . . 5<br>DON'T KNOW 8
431	Was (NAME) weighed at birth?	YES . . . . 1<br>NO . . . . 2<br>(SKIP TO 433)<br>DON'T KNOW 8	YES . . . . 1<br>NO . . . . 2<br>(SKIP TO 433)<br>DON'T KNOW 8	YES . . . . 1<br>NO . . . . 2<br>(SKIP TO 433)<br>DON'T KNOW 8
432	How much did (NAME) weigh?<br><br>RECORD WEIGHT IN KILOGRAMS FROM HEALTH CARD, IF AVAILABLE.	KG FROM CARD<br>1 ☐ . ☐☐☐<br>KG FROM RECALL<br>2 ☐ . ☐☐☐<br>DON'T KNOW 99998	KG FROM CARD<br>1 ☐ . ☐☐☐<br>KG FROM RECALL<br>2 ☐ . ☐☐☐<br>DON'T KNOW 99998	KG FROM CARD<br>1 ☐ . ☐☐☐<br>KG FROM RECALL<br>2 ☐ . ☐☐☐<br>DON'T KNOW 99998
433	Who assisted with the delivery of (NAME)? **(2)**<br><br>Anyone else?<br><br>PROBE FOR THE TYPE(S) OF PERSON(S) AND RECORD ALL MENTIONED.<br><br>IF RESPONDENT SAYS NO ONE ASSISTED, PROBE TO DETERMINE WHETHER ANY ADULTS WERE PRESENT AT THE DELIVERY.	HEALTH PERSONNEL<br>DOCTOR . . . . . A<br>NURSE/MIDWIFE B<br>AUXILIARY MIDWIFE . . . C<br>OTHER PERSON<br>TRADITIONAL BIRTH ATTENDANT . . D<br>RELATIVE/FRIEND . E<br>OTHER ________ X<br>(SPECIFY)<br>NO ONE ASSISTED Y	HEALTH PERSONNEL<br>DOCTOR . . . . A<br>NURSE/MIDWIFE B<br>AUXILIARY MIDWIFE . . . C<br>OTHER PERSON<br>TRADITIONAL BIRTH ATTENDANT . . D<br>RELATIVE/FRIEND . E<br>OTHER ________ X<br>(SPECIFY)<br>NO ONE ASSISTED Y	HEALTH PERSONNEL<br>DOCTOR . . . . . A<br>NURSE/MIDWIFE B<br>AUXILIARY MIDWIFE . . . C<br>OTHER PERSON<br>TRADITIONAL BIRTH ATTENDANT . . D<br>RELATIVE/FRIEND . E<br>OTHER ________ X<br>(SPECIFY)<br>NO ONE ASSISTED Y

NO.	QUESTIONS AND FILTERS	LAST BIRTH<br>NAME ________	NEXT-TO-LAST BIRTH<br>NAME ________	SECOND-FROM-LAST BIRTH<br>NAME ________
434	Where did you give birth to (NAME)? **(2)**<br><br>PROBE TO IDENTIFY THE TYPE OF SOURCE.<br><br>IF UNABLE TO DETERMINE IF PUBLIC OR PRIVATE SECTOR, WRITE THE NAME OF THE PLACE.<br><br>________<br>(NAME OF PLACE)	HOME<br>YOUR HOME . . . 11<br>(SKIP TO 438)<br>OTHER HOME . . . 12<br><br>PUBLIC SECTOR<br>GOVT. HOSPITAL 21<br>GOVT. HEALTH CENTER . . . . . 22<br>GOVT. HEALTH POST . . . . 23<br>OTHER PUBLIC SECTOR ________ 26<br>(SPECIFY)<br><br>PRIVATE MED. SECTOR<br>PVT. HOSPITAL/ CLINIC . . . . 31<br>OTHER PRIVATE MED. SECTOR ________ 36<br>(SPECIFY)<br><br>OTHER________ 96<br>(SPECIFY)<br>(SKIP TO 438)	HOME<br>YOUR HOME . . . 11<br>(SKIP TO 448)<br>OTHER HOME . . . 12<br><br>PUBLIC SECTOR<br>GOVT. HOSPITAL 21<br>GOVT. HEALTH CENTER . . . . . 22<br>GOVT. HEALTH POST . . . . 23<br>OTHER PUBLIC SECTOR ________ 26<br>(SPECIFY)<br><br>PRIVATE MED. SECTOR<br>PVT. HOSPITAL/ CLINIC . . . . 31<br>OTHER PRIVATE MED. SECTOR ________ 36<br>(SPECIFY)<br><br>OTHER________ 96<br>(SPECIFY)<br>(SKIP TO 448)	HOME<br>YOUR HOME . . . 11<br>(SKIP TO 448)<br>OTHER HOME . . . 12<br><br>PUBLIC SECTOR<br>GOVT. HOSPITAL 21<br>GOVT. HEALTH CENTER . . . . . 22<br>GOVT. HEALTH POST . . . . 23<br>OTHER PUBLIC SECTOR ________ 26<br>(SPECIFY)<br><br>PRIVATE MED. SECTOR<br>PVT. HOSPITAL/ CLINIC . . . . 31<br>OTHER PRIVATE MED. SECTOR ________ 36<br>(SPECIFY)<br><br>OTHER________ 96<br>(SPECIFY)<br>(SKIP TO 448)
434A	How long after (NAME) was delivered did you stay there?<br><br>IF LESS THAN ONE DAY, RECORD HOURS.<br>IF LESS THAN ONE WEEK, RECORD DAYS.	HOURS 1 [ ][ ]<br>DAYS 2 [ ][ ]<br>WEEKS 3 [ ][ ]<br><br>DON'T KNOW . . . 998		
435	Was (NAME) delivered by caesarean, that is, did they cut your belly open to take the baby out?	YES . . . . 1<br>NO 2	YES . . . . 1<br>NO 2	YES . . . . 1<br>NO 2
436	I would like to talk to you about checks on your health after delivery, for example, someone asking you questions about your health or examining you. Did anyone check on your health while you were still in the facility?	YES . . . . 1<br>(SKIP TO 439)<br>NO 2		
437	Did anyone check on your health after you left the facility?	YES . . . . 1<br>(SKIP TO 439)<br>NO . . . . 2<br>(SKIP TO 442)		

NO.	QUESTIONS AND FILTERS	LAST BIRTH<br>NAME ________	NEXT-TO-LAST BIRTH<br>NAME ________	SECOND-FROM-LAST BIRTH<br>NAME ________
438	I would like to talk to you about checks on your health after delivery, for example, someone asking you questions about your health or examining you. Did anyone check on your health after you gave birth to (NAME)?	YES . . . . 1<br>NO . . . . 2<br>(SKIP TO 442)		
439	Who checked on your health at that time? **(2)**<br><br>PROBE FOR MOST QUALIFIED PERSON.	HEALTH PERSONNEL<br>DOCTOR . . . . 11<br>NURSE/MIDWIFE 12<br>AUXILIARY<br>MIDWIFE . . . . . 13<br>OTHER PERSON<br>TRADITIONAL BIRTH<br>ATTENDANT 21<br>COMMUNITY/<br>VILLAGE HEALTH<br>WORKER . . . 22<br><br>OTHER ____________ 96<br>(SPECIFY)		
440	How long after delivery did the first check take place?<br><br>IF LESS THAN ONE DAY, RECORD HOURS.<br>IF LESS THAN ONE WEEK, RECORD DAYS.	HOURS 1 [ ][ ]<br>DAYS 2 [ ][ ]<br>WEEKS 3 [ ][ ]<br><br>DON'T KNOW . . . 998		
442	In the two months after (NAME) was born, did any health care provider or a traditional birth attendant check on his/her health?	YES . . . . 1<br>NO . . . . 2<br>(SKIP TO 446)<br>DON'T KNOW 8		
443	How many hours, days or weeks after the birth of (NAME) did the first check take place?<br><br>IF LESS THAN ONE DAY, RECORD HOURS.<br>IF LESS THAN ONE WEEK, RECORD DAYS.	HRS AFTER<br>BIRTH . . 1 [ ][ ]<br>DAYS AFTER<br>BIRTH . . 2 [ ][ ]<br>WKS AFTER<br>BIRTH . . 3 [ ][ ]<br><br>DON'T KNOW . . . 998		
444	Who checked on (NAME)'s health at that time? **(2)**<br><br>PROBE FOR MOST QUALIFIED PERSON.	HEALTH PERSONNEL<br>DOCTOR . . . . 11<br>NURSE/MIDWIFE 12<br>AUXILIARY<br>MIDWIFE . . . . . 13<br>OTHER PERSON<br>TRADITIONAL BIRTH<br>ATTENDANT 21<br>COMMUNITY/<br>VILLAGE HEALTH<br>WORKER . . . 22<br><br>OTHER ____________ 96<br>(SPECIFY)		

NO.	QUESTIONS AND FILTERS	LAST BIRTH<br>NAME ________	NEXT-TO-LAST BIRTH<br>NAME ________	SECOND-FROM-LAST BIRTH<br>NAME ________
445	Where did this first check of (NAME) take place? **(2)**<br><br>PROBE TO IDENTIFY THE TYPE OF SOURCE AND CIRCLE THE APPROPRIATE CODE.<br><br>IF UNABLE TO DETERMINE IF PUBLIC OR PRIVATE SECTOR, WRITE THE NAME OF THE PLACE.<br><br>________<br>(NAME OF PLACE)	HOME<br>YOUR HOME . . . 11<br>OTHER HOME . . . 12<br><br>PUBLIC SECTOR<br>GOVT. HOSPITAL 21<br>GOVT. HEALTH CENTER . . . . . 22<br>GOVT. HEALTH POST . . . . 23<br>OTHER PUBLIC ________ 26<br>(SPECIFY)<br><br>PRIVATE MED. SECTOR<br>PVT. HOSPITAL/ CLINIC . . . . 31<br>OTHER PRIVATE MED. ________ 36<br>(SPECIFY)<br><br>OTHER ________ 96<br>(SPECIFY)		
446	In the first two months after delivery, did you receive a vitamin A dose like (this/any of these)?<br><br>SHOW COMMON TYPES OF AMPULES/CAPSULES/SYRUPS.	YES . . . . 1<br>NO . . . . 2<br>DON'T KNOW 8		
447	Has your menstrual period returned since the birth of (NAME)?	YES . . . . 1<br>(SKIP TO 449)←<br>NO . . . . 2<br>(SKIP TO 450)←		
448	Did your period return between the birth of (NAME) and your next pregnancy?		YES . . . . 1<br>NO . . . . 2<br>(SKIP TO 452)←	YES . . . . 1<br>NO . . . . 2<br>(SKIP TO 452)←
449	For how many months after the birth of (NAME) did you not have a period?	MONTHS . . . [ ][ ]<br>DON'T KNOW 98	MONTHS . . . [ ][ ]<br>DON'T KNOW 98	MONTHS . . . [ ][ ]<br>DON'T KNOW 98
450	CHECK 226:<br><br>IS RESPONDENT PREGNANT?	NOT PREG-NANT [ ]↓<br>PREGNANT OR UNSURE [ ]<br>(SKIP TO 452)←		
451	Have you had sexual intercourse since the birth of (NAME)?	YES . . . . 1<br>NO . . . . 2<br>(SKIP TO 453)←		

NO.	QUESTIONS AND FILTERS	LAST BIRTH<br>NAME ________	NEXT-TO-LAST BIRTH<br>NAME ________	SECOND-FROM-LAST BIRTH<br>NAME ________
452	For how many months after the birth of (NAME) did you not have sexual intercourse?	MONTHS . . . [ ][ ]<br>DON'T KNOW 98	MONTHS . . . [ ][ ]<br>DON'T KNOW 98	MONTHS . . . [ ][ ]<br>DON'T KNOW 98
453	Did you ever breastfeed (NAME)?	YES . . . . 1<br>(SKIP TO 455)←<br>NO 2	YES . . . . 1<br>NO 2	YES . . . . 1<br>NO 2
454	CHECK 404:<br><br>IS CHILD LIVING?	LIVING [ ] (SKIP TO 460)<br>DEAD [ ] (GO BACK TO 405 IN NEXT COLUMN; OR IF NO MORE BIRTHS, GO TO 501)		
455	How long after birth did you first put (NAME) to the breast?<br><br>IF LESS THAN 1 HOUR, RECORD '00' HOURS.<br>IF LESS THAN 24 HOURS, RECORD HOURS.<br>OTHERWISE, RECORD DAYS.	IMMEDIATELY . . . 000<br><br>HOURS 1 [ ][ ]<br>DAYS 2 [ ][ ]		
456	In the first three days after delivery, was (NAME) given anything to drink other than breast milk?	YES . . . . 1<br>NO . . . . 2<br>(SKIP TO 458)←		
457	What was (NAME) given to drink?<br><br>Anything else?<br><br>RECORD ALL LIQUIDS MENTIONED.	MILK (OTHER THAN BREAST MILK) A<br>PLAIN WATER . . . B<br>SUGAR OR GLU-COSE WATER . . . C<br>GRIPE WATER . . . D<br>SUGAR-SALT-WATER SOLUTION . . . . . E<br>FRUIT JUICE . . . . . F<br>INFANT FORMULA G<br>TEA/INFUSIONS . . . H<br>COFFEE . . . . I<br>HONEY . . . . J<br><br>OTHER ________ X<br>(SPECIFY)		
458	CHECK 404:<br><br>IS CHILD LIVING?	LIVING [ ] ↓<br>DEAD [ ] (GO BACK TO 405 IN NEXT COLUMN; OR, IF NO MORE BIRTHS, GO TO 501)	LIVING [ ] ↓<br>DEAD [ ] (GO BACK TO 405 IN NEXT COLUMN; OR, IF NO MORE BIRTHS, GO TO 501)	LIVING [ ] ↓<br>DEAD [ ] (GO BACK TO 405 IN NEXT-TO-LAST COLUMN OF NEW QUESTIONNAIRE; OR, IF NO MORE BIRTHS, GO TO 501)

NO.	QUESTIONS AND FILTERS	LAST BIRTH<br>NAME _______________	NEXT-TO-LAST BIRTH<br>NAME _______________	SECOND-FROM-LAST BIRTH<br>NAME _______________
459	Are you still breastfeeding (NAME)?	YES . . . . 1<br>NO 2		
460	Did (NAME) drink anything from a bottle with a nipple yesterday or last night?	YES . . . . 1<br>NO . . . . 2<br>DON'T KNOW 8	YES . . . . 1<br>NO . . . . 2<br>DON'T KNOW 8	YES . . . . 1<br>NO . . . . 2<br>DON'T KNOW 8
461		GO BACK TO 405 IN NEXT COLUMN; OR, IF NO MORE BIRTHS, GO TO 501.	GO BACK TO 405 IN NEXT COLUMN; OR, IF NO MORE BIRTHS, GO TO 501.	GO BACK TO 405 IN NEXT-TO-LAST COLUMN OF NEW QUESTIONNAIRE; OR, IF NO MORE BIRTHS, GO TO 501.

(1) Year of fieldwork is assumed to be 2010. For fieldwork beginning in 2011 or 2012, the year should be 2006 or 2007, respectively.

(2) Coding categories to be developed locally and revised based on the pretest; however, the broad categories must be maintained.

(3) Vaccination practices may vary; this question should specify where the injection is given, e.g. arm or shoulder.

(4) Syrup should be deleted in countries where syrup is not used.

(5) In countries where it is important to know the number of iron tablets taken per day, an appropriate question may be added.

(6) The question should be deleted in surveys in countries where there is no program for intermittent preventive treatment against malaria during pregnancy.

SECTION 5. CHILD IMMUNIZATION, HEALTH AND NUTRITION

501	ENTER IN THE TABLE THE BIRTH HISTORY NUMBER, NAME, AND SURVIVAL STATUS OF EACH BIRTH IN 2005**(1)** OR LATER.<br>ASK THE QUESTIONS ABOUT ALL OF THESE BIRTHS. BEGIN WITH THE LAST BIRTH.<br>(IF THERE ARE MORE THAN 3 BIRTHS, USE LAST 2 COLUMNS OF ADDITIONAL QUESTIONNAIRES).			
502	BIRTH HISTORY NUMBER FROM 212 IN BIRTH HISTORY	LAST BIRTH<br>BIRTH HISTORY NUMBER	NEXT-TO-LAST BIRTH<br>BIRTH HISTORY NUMBER	SECOND-FROM-LAST BIRTH<br>BIRTH HISTORY NUMBER . . . .
503	FROM 212 AND 216	NAME ______<br>LIVING / DEAD<br>(GO TO 503 IN NEXT COLUMN OR, IF NO MORE BIRTHS, GO TO 553)	NAME ______<br>LIVING / DEAD<br>(GO TO 503 IN NEXT COLUMN OR, IF NO MORE BIRTHS, GO TO 553)	NAME ______<br>LIVING / DEAD<br>(GO TO 503 IN NEXT-TO-LAST COLUMN OF NEW QUESTIONNAIRE, OR IF NO MORE BIRTHS, GO TO 553)
504	Do you have a card where (NAME)'s vaccinations are written down? **(2)**<br>IF YES:<br>May I see it please?	YES, SEEN . . . . 1<br>(SKIP TO 506)<br>YES, NOT SEEN . . . . 2<br>(SKIP TO 509)<br>NO CARD 3	YES, SEEN . . . . 1<br>(SKIP TO 506)<br>YES, NOT SEEN . . . . 2<br>(SKIP TO 509)<br>NO CARD 3	YES, SEEN . . . . 1<br>(SKIP TO 506)<br>YES, NOT SEEN . . . . 2<br>(SKIP TO 509)<br>NO CARD 3
505	Did you ever have a vaccination card for (NAME)? **(2)**	YES . . . . 1<br>(SKIP TO 509)<br>NO 2	YES . . . . 1<br>(SKIP TO 509)<br>NO 2	YES . . . . 1<br>(SKIP TO 509)<br>NO 2

506
(1) COPY DATES FROM THE CARD. **(2)**
(2) WRITE '44' IN 'DAY' COLUMN IF CARD SHOWS THAT A DOSE WAS GIVEN, BUT NO DATE IS RECORDED.

	LAST BIRTH DAY	MONTH	YEAR		NEXT-TO-LAST BIRTH DAY	MONTH	YEAR		SECOND-FROM-LAST BIRTH DAY	MONTH	YEAR
BCG				BCG				BCG			
POLIO 0 (POLIO GIVEN AT BIRTH)				P0				P0			
POLIO 1				P1				P1			
POLIO 2				P2				P2			
POLIO 3				P3				P3			
DPT 1				D1				D1			
DPT 2				D2				D2			
DPT 3				D3				D3			
MEASLES				MEA				MEA			
VITAMIN A (MOST RECENT)				VIT A				VIT A			

507	CHECK 506:	BCG TO MEASLES ALL RECORDED **(3)** (GO TO 511) / OTHER	BCG TO MEASLES ALL RECORDED **(3)** (GO TO 511) / OTHER	BCG TO MEASLES ALL RECORDED **(3)** (GO TO 511) / OTHER

NO.	QUESTIONS AND FILTERS	LAST BIRTH<br>NAME ________	NEXT-TO-LAST BIRTH<br>NAME ________	SECOND-FROM-LAST BIRTH<br>NAME ________
508	Has (NAME) had any vaccinations that are not recorded on this card, including vaccinations given in a national immunization day campaign?<br><br>RECORD 'YES' ONLY IF THE RESPONDENT MENTIONS AT LEAST ONE OF THE VACCINATIONS IN 506 THAT ARE NOT RECORDED AS HAVING BEEN GIVEN.	YES . . . . 1<br>(PROBE FOR VACCINATIONS AND WRITE '66' IN THE CORRESPONDING DAY COLUMN IN 506)<br>(SKIP TO 511)<br>NO . . . . 2<br>(SKIP TO 511)<br>DON'T KNOW 8	YES . . . . 1<br>(PROBE FOR VACCINATIONS AND WRITE '66' IN THE CORRESPONDING DAY COLUMN IN 506)<br>(SKIP TO 511)<br>NO . . . . 2<br>(SKIP TO 511)<br>DON'T KNOW 8	YES . . . . 1<br>(PROBE FOR VACCINATIONS AND WRITE '66' IN THE CORRESPONDING DAY COLUMN IN 506)<br>(SKIP TO 511)<br>NO . . . . 2<br>(SKIP TO 511)<br>DON'T KNOW 8
509	Did (NAME) ever have any vaccinations to prevent him/her from getting diseases, including vaccinations received in a national immunization day campaign?	YES . . . . 1<br>NO . . . . 2<br>(SKIP TO 511)<br>DON'T KNOW 8	YES . . . . 1<br>NO . . . . 2<br>(SKIP TO 511)<br>DON'T KNOW 8	YES . . . . 1<br>NO . . . . 2<br>(SKIP TO 511)<br>DON'T KNOW 8
510	Please tell me if (NAME) had any of the following vaccinations: **(4)**			
510A	A BCG vaccination against tuberculosis, that is, an injection in the arm or shoulder that usually causes a scar? **(5)**	YES . . . . 1<br>NO . . . . 2<br>DON'T KNOW 8	YES . . . . 1<br>NO . . . . 2<br>DON'T KNOW 8	YES . . . . 1<br>NO . . . . 2<br>DON'T KNOW 8
510B	Polio vaccine, that is, drops in the mouth?	YES . . . . 1<br>NO . . . . 2<br>(SKIP TO 510E)<br>DON'T KNOW 8	YES . . . . 1<br>NO . . . . 2<br>(SKIP TO 510E)<br>DON'T KNOW 8	YES . . . . 1<br>NO . . . . 2<br>(SKIP TO 510E)<br>DON'T KNOW 8
510C	Was the first polio vaccine given in the first two weeks after birth or later? **(6)**	FIRST 2 WEEKS . . . 1<br>LATER 2	FIRST 2 WEEKS . . . 1<br>LATER 2	FIRST 2 WEEKS . . . 1<br>LATER 2
510D	How many times was the polio vaccine given?	NUMBER OF TIMES . . . . . ☐	NUMBER OF TIMES . . . . . ☐	NUMBER OF TIMES . . . . . ☐
510E	A DPT vaccination, that is, an injection given in the thigh or buttocks, sometimes at the same time as polio drops? **(5)**	YES . . . . 1<br>NO . . . . 2<br>(SKIP TO 510G)<br>DON'T KNOW 8	YES . . . . 1<br>NO . . . . 2<br>(SKIP TO 510G)<br>DON'T KNOW 8	YES . . . . 1<br>NO . . . . 2<br>(SKIP TO 510G)<br>DON'T KNOW 8
510F	How many times was the DPT vaccination given?	NUMBER OF TIMES . . . . . ☐	NUMBER OF TIMES . . . . . ☐	NUMBER OF TIMES . . . . . ☐
510G	A measles injection or an MMR injection - that is, a shot in the arm at the age of 9 months or older - to prevent him/her from getting measles? **(7)**	YES . . . . 1<br>NO . . . . 2<br>DON'T KNOW 8	YES . . . . 1<br>NO . . . . 2<br>DON'T KNOW 8	YES . . . . 1<br>NO . . . . 2<br>DON'T KNOW 8

NO.	QUESTIONS AND FILTERS	LAST BIRTH<br>NAME ________	NEXT-TO-LAST BIRTH<br>NAME ________	SECOND-FROM-LAST BIRTH<br>NAME ________
511	Within the last six months, was (NAME) given a vitamin A dose like (this/any of these)?<br><br>SHOW COMMON TYPES OF AMPULES/CAPSULES/SYRUPS.	YES . . . . 1<br>NO . . . . 2<br>DON'T KNOW 8	YES . . . . 1<br>NO . . . . 2<br>DON'T KNOW 8	YES . . . . 1<br>NO . . . . 2<br>DON'T KNOW 8
512	In the last seven days, was (NAME) given iron pills, sprinkles with iron, or iron syrup like (this/any of these)?<br><br>SHOW COMMON TYPES OF PILLS/SPRINKLES/ SYRUPS.	YES . . . . 1<br>NO . . . . 2<br>DON'T KNOW 8	YES . . . . 1<br>NO . . . . 2<br>DON'T KNOW 8	YES . . . . 1<br>NO . . . . 2<br>DON'T KNOW 8
513	Was (NAME) given any drug for intestinal worms in the last six months?	YES . . . . 1<br>NO . . . . 2<br>DON'T KNOW 8	YES . . . . 1<br>NO . . . . 2<br>DON'T KNOW 8	YES . . . . 1<br>NO . . . . 2<br>DON'T KNOW 8
514	Has (NAME) had diarrhea in the last 2 weeks? **(8)**	YES . . . . 1<br>NO . . . . 2<br>(SKIP TO 525)<br>DON'T KNOW 8	YES . . . . 1<br>NO . . . . 2<br>(SKIP TO 525)<br>DON'T KNOW 8	YES . . . . 1<br>NO . . . . 2<br>(SKIP TO 525)<br>DON'T KNOW 8
515	Was there any blood in the stools?	YES . . . . 1<br>NO . . . . 2<br>DON'T KNOW 8	YES . . . . 1<br>NO . . . . 2<br>DON'T KNOW 8	YES . . . . 1<br>NO . . . . 2<br>DON'T KNOW 8
516	Now I would like to know how much (NAME) was given to drink during the diarrhea (including breastmilk).<br><br>Was he/she given less than usual to drink, about the same amount, or more than usual to drink?<br><br>IF LESS, PROBE: Was he/she given much less than usual to drink or somewhat less?	MUCH LESS . . . . . 1<br>SOMEWHAT LESS 2<br>ABOUT THE SAME 3<br>MORE . . . . 4<br>NOTHING TO DRINK 5<br>DON'T KNOW 8	MUCH LESS . . . . . 1<br>SOMEWHAT LESS 2<br>ABOUT THE SAME 3<br>MORE . . . . 4<br>NOTHING TO DRINK 5<br>DON'T KNOW 8	MUCH LESS . . . . . 1<br>SOMEWHAT LESS 2<br>ABOUT THE SAME 3<br>MORE . . . . 4<br>NOTHING TO DRINK 5<br>DON'T KNOW 8
517	When (NAME) had diarrhea, was he/she given less than usual to eat, about the same amount, more than usual, or nothing to eat?<br><br>IF LESS, PROBE: Was he/she given much less than usual to eat or somewhat less?	MUCH LESS . . . . . 1<br>SOMEWHAT LESS 2<br>ABOUT THE SAME 3<br>MORE . . . . 4<br>STOPPED FOOD 5<br>NEVER GAVE FOOD 6<br>DON'T KNOW 8	MUCH LESS . . . . . 1<br>SOMEWHAT LESS 2<br>ABOUT THE SAME 3<br>MORE . . . . 4<br>STOPPED FOOD 5<br>NEVER GAVE FOOD 6<br>DON'T KNOW 8	MUCH LESS . . . . . 1<br>SOMEWHAT LESS 2<br>ABOUT THE SAME 3<br>MORE . . . . 4<br>STOPPED FOOD 5<br>NEVER GAVE FOOD 6<br>DON'T KNOW 8
518	Did you seek advice or treatment for the diarrhea from any source?	YES . . . . 1<br>NO . . . . 2<br>(SKIP TO 522)	YES . . . . 1<br>NO . . . . 2<br>(SKIP TO 522)	YES . . . . 1<br>NO . . . . 2<br>(SKIP TO 522)

<table>
<tr><th>NO.</th><th>QUESTIONS AND FILTERS</th><th>LAST BIRTH
NAME ______________</th><th>NEXT-TO-LAST BIRTH
NAME ______________</th><th>SECOND-FROM-LAST BIRTH
NAME ______________</th></tr>
<tr><td>519</td><td>Where did you seek advice or treatment? (9)

Anywhere else?

PROBE TO IDENTIFY EACH TYPE OF SOURCE.

IF UNABLE TO DETERMINE IF PUBLIC OR PRIVATE SECTOR, WRITE THE NAME OF THE PLACE.

____
(NAME OF PLACE(S))</td><td>PUBLIC SECTOR
GOVT HOSPITAL A
GOVT HEALTH CENTER ..... B
GOVT HEALTH POST C
MOBILE CLINIC D
FIELDWORKER E
OTHER PUBLIC SECTOR
______________ F
(SPECIFY)

PRIVATE MEDICAL SECTOR
PVT. HOSPITAL/ CLINIC .... G
PHARMACY ... H
PVT DOCTOR ... I
MOBILE CLINIC J
FIELDWORKER K
OTHER PRIVATE MED. SECTOR
______________ L
(SPECIFY)

OTHER SOURCE
SHOP .... M
TRADITIONAL PRACTITIONER N
MARKET ..... O

OTHER __________ X
(SPECIFY)</td><td>PUBLIC SECTOR
GOVT HOSPITAL A
GOVT HEALTH CENTER ..... B
GOVT HEALTH POST C
MOBILE CLINIC D
FIELDWORKER E
OTHER PUBLIC SECTOR
______________ F
(SPECIFY)

PRIVATE MEDICAL SECTOR
PVT. HOSPITAL/ CLINIC .... G
PHARMACY ... H
PVT DOCTOR ... I
MOBILE CLINIC J
FIELDWORKER K
OTHER PRIVATE MED. SECTOR
______________ L
(SPECIFY)

OTHER SOURCE
SHOP .... M
TRADITIONAL PRACTITIONER N
MARKET ..... O

OTHER __________ X
(SPECIFY)</td><td>PUBLIC SECTOR
GOVT HOSPITAL A
GOVT HEALTH CENTER ..... B
GOVT HEALTH POST C
MOBILE CLINIC D
FIELDWORKER E
OTHER PUBLIC SECTOR
______________ F
(SPECIFY)

PRIVATE MEDICAL SECTOR
PVT. HOSPITAL/ CLINIC .... G
PHARMACY ... H
PVT DOCTOR ... I
MOBILE CLINIC J
FIELDWORKER K
OTHER PRIVATE MED. SECTOR
______________ L
(SPECIFY)

OTHER SOURCE
SHOP .... M
TRADITIONAL PRACTITIONER N
MARKET ..... O

OTHER __________ X
(SPECIFY)</td></tr>
<tr><td>520</td><td>CHECK 519:</td><td>TWO OR MORE CODES CIRCLED ☐
ONLY ONE CODE CIRCLED ☐
(SKIP TO 522)</td><td>TWO OR MORE CODES CIRCLED ☐
ONLY ONE CODE CIRCLED ☐
(SKIP TO 522)</td><td>TWO OR MORE CODES CIRCLED ☐
ONLY ONE CODE CIRCLED ☐
(SKIP TO 522)</td></tr>
<tr><td>521</td><td>Where did you first seek advice or treatment?

USE LETTER CODE FROM 519.</td><td>FIRST PLACE ... ☐</td><td>FIRST PLACE ... ☐</td><td>FIRST PLACE ... ☐</td></tr>
<tr><td>522</td><td>Was he/she given any of the following to drink at any time since he/she started having the diarrhea:</td><td>YES NO DK</td><td>YES NO DK</td><td>YES NO DK</td></tr>
<tr><td></td><td>a) A fluid made from a special packet called [LOCAL NAME FOR ORS PACKET]?</td><td>FLUID FROM ORS PKT 1 2 8</td><td>FLUID FROM ORS PKT 1 2 8</td><td>FLUID FROM ORS PKT 1 2 8</td></tr>
<tr><td></td><td>b) A pre-packaged ORS liquid? (10)</td><td>ORS LQD 1 2 8</td><td>ORS LQD 1 2 8</td><td>ORS LQD 1 2 8</td></tr>
<tr><td></td><td>c) A government-recommended homemade fluid? (11)</td><td>HOMEMADE FLUID ... 1 2 8</td><td>HOMEMADE FLUID ... 1 2 8</td><td>HOMEMADE FLUID ... 1 2 8</td></tr>
</table>

NO.	QUESTIONS AND FILTERS	LAST BIRTH<br>NAME ________	NEXT-TO-LAST BIRTH<br>NAME ________	SECOND-FROM-LAST BIRTH<br>NAME ________
523	Was anything (else) given to treat the diarrhea?	YES . . . . 1<br>NO . . . . 2<br>(SKIP TO 525)←<br>DON'T KNOW 8	YES . . . . 1<br>NO . . . . 2<br>(SKIP TO 525)←<br>DON'T KNOW 8	YES . . . . 1<br>NO . . . . 2<br>(SKIP TO 525)←<br>DON'T KNOW 8
524	What (else) was given to treat the diarrhea?<br><br>Anything else?<br><br>RECORD ALL TREATMENTS GIVEN.	PILL OR SYRUP<br>ANTIBIOTIC . . . . . A<br>ANTIMOTILITY B<br>ZINC . . . . C<br>OTHER (NOT ANTI-BIOTIC, ANTI-MOTILITY, OR ZINC) . . . . D<br>UNKNOWN PILL OR SYRUP . . . E<br><br>INJECTION<br>ANTIBIOTIC . . . . . F<br>NON-ANTIBIOTIC G<br>UNKNOWN INJECTION . . . H<br><br>(IV) INTRAVENOUS I<br><br>HOME REMEDY/ HERBAL MED-ICINE . . . . J<br><br>OTHER ________ X<br>(SPECIFY)	PILL OR SYRUP<br>ANTIBIOTIC . . . . . A<br>ANTIMOTILITY B<br>ZINC . . . . C<br>OTHER (NOT ANTI-BIOTIC, ANTI-MOTILITY, OR ZINC) . . . . D<br>UNKNOWN PILL OR SYRUP . . . E<br><br>INJECTION<br>ANTIBIOTIC . . . . . F<br>NON-ANTIBIOTIC G<br>UNKNOWN INJECTION . . . H<br><br>(IV) INTRAVENOUS I<br><br>HOME REMEDY/ HERBAL MED-ICINE . . . . J<br><br>OTHER ________ X<br>(SPECIFY)	PILL OR SYRUP<br>ANTIBIOTIC . . . . . A<br>ANTIMOTILITY B<br>ZINC . . . . C<br>OTHER (NOT ANTI-BIOTIC, ANTI-MOTILITY, OR ZINC) . . . . D<br>UNKNOWN PILL OR SYRUP . . . E<br><br>INJECTION<br>ANTIBIOTIC . . . . . F<br>NON-ANTIBIOTIC G<br>UNKNOWN INJECTION . . . H<br><br>(IV) INTRAVENOUS I<br><br>HOME REMEDY/ HERBAL MED-ICINE . . . . J<br><br>OTHER ________ X<br>(SPECIFY)
525	Has (NAME) been ill with a fever at any time in the last 2 weeks?	YES . . . . 1<br>NO . . . . 2<br>(SKIP TO 527)←<br>DON'T KNOW 8	YES . . . . 1<br>NO . . . . 2<br>(SKIP TO 527)←<br>DON'T KNOW 8	YES . . . . 1<br>NO . . . . 2<br>(SKIP TO 527)←<br>DON'T KNOW 8
526<br>**(12)**	At any time during the illness, did (NAME) have blood taken from his/her finger or heel for testing?	YES . . . . 1<br>NO . . . . 2<br>DON'T KNOW 8	YES . . . . 1<br>NO . . . . 2<br>DON'T KNOW 8	YES . . . . 1<br>NO . . . . 2<br>DON'T KNOW 8
527	Has (NAME) had an illness with a cough at any time in the last 2 weeks?	YES . . . . 1<br>NO . . . . 2<br>(SKIP TO 530)←<br>DON'T KNOW 8	YES . . . . 1<br>NO . . . . 2<br>(SKIP TO 530)←<br>DON'T KNOW 8	YES . . . . 1<br>NO . . . . 2<br>(SKIP TO 530)←<br>DON'T KNOW 8
528	When (NAME) had an illness with a cough, did he/she breathe faster than usual with short, rapid breaths or have difficulty breathing?	YES . . . . 1<br>NO . . . . 2<br>(SKIP TO 531)←<br>DON'T KNOW 8	YES . . . . 1<br>NO . . . . 2<br>(SKIP TO 531)←<br>DON'T KNOW 8	YES . . . . 1<br>NO . . . . 2<br>(SKIP TO 531)←<br>DON'T KNOW 8
529	Was the fast or difficult breathing due to a problem in the chest or to a blocked or runny nose?	CHEST ONLY . . . 1<br>NOSE ONLY . . . . . 2<br>BOTH . . . . 3<br>OTHER ________ 6<br>(SPECIFY)<br>DON'T KNOW . . . . . 8<br>(SKIP TO 531)←	CHEST ONLY . . . 1<br>NOSE ONLY . . . . . 2<br>BOTH . . . . 3<br>OTHER ________ 6<br>(SPECIFY)<br>DON'T KNOW . . . . . 8<br>(SKIP TO 531)←	CHEST ONLY . . . 1<br>NOSE ONLY . . . . . 2<br>BOTH . . . . 3<br>OTHER ________ 6<br>(SPECIFY)<br>DON'T KNOW . . . . . 8<br>(SKIP TO 531)←

NO.	QUESTIONS AND FILTERS	LAST BIRTH<br>NAME ________	NEXT-TO-LAST BIRTH<br>NAME ________	SECOND-FROM-LAST BIRTH<br>NAME ________
530	CHECK 525:<br>HAD FEVER?	YES ☐ NO OR DK ☐<br>(GO BACK TO 503 IN NEXT COLUMN; OR, IF NO MORE BIRTHS, GO TO 553)	YES ☐ NO OR DK ☐<br>(GO BACK TO 503 IN NEXT COLUMN; OR, IF NO MORE BIRTHS, GO TO 553)	YES ☐ NO OR DK ☐<br>(GO TO 503 IN NEXT-TO-LAST COLUMN OF NEW QUESTIONNAIRE; OR, IF NO MORE BIRTHS, GO TO 553)
531	Now I would like to know how much (NAME) was given to drink (including breastmilk) during the illness with a (fever/cough).<br>Was he/she given less than usual to drink, about the same amount, or more than usual to drink?<br>IF LESS, PROBE: Was he/she given much less than usual to drink or somewhat less?	MUCH LESS ..... 1<br>SOMEWHAT LESS 2<br>ABOUT THE SAME 3<br>MORE .... 4<br>NOTHING TO DRINK 5<br>DON'T KNOW 8	MUCH LESS ..... 1<br>SOMEWHAT LESS 2<br>ABOUT THE SAME 3<br>MORE .... 4<br>NOTHING TO DRINK 5<br>DON'T KNOW 8	MUCH LESS ..... 1<br>SOMEWHAT LESS 2<br>ABOUT THE SAME 3<br>MORE .... 4<br>NOTHING TO DRINK 5<br>DON'T KNOW 8
532	When (NAME) had a (fever/cough), was he/she given less than usual to eat, about the same amount, more than usual, or nothing to eat?<br>IF LESS, PROBE: Was he/she given much less than usual to eat or somewhat less?	MUCH LESS ..... 1<br>SOMEWHAT LESS 2<br>ABOUT THE SAME 3<br>MORE .... 4<br>STOPPED FOOD 5<br>NEVER GAVE FOOD 6<br>DON'T KNOW 8	MUCH LESS ..... 1<br>SOMEWHAT LESS 2<br>ABOUT THE SAME 3<br>MORE .... 4<br>STOPPED FOOD 5<br>NEVER GAVE FOOD 6<br>DON'T KNOW 8	MUCH LESS ..... 1<br>SOMEWHAT LESS 2<br>ABOUT THE SAME 3<br>MORE .... 4<br>STOPPED FOOD 5<br>NEVER GAVE FOOD 6<br>DON'T KNOW 8
533	Did you seek advice or treatment for the illness from any source?	YES .... 1<br>NO .... 2<br>(SKIP TO 537)	YES .... 1<br>NO .... 2<br>(SKIP TO 537)	YES .... 1<br>NO .... 2<br>(SKIP TO 537)

NO.	QUESTIONS AND FILTERS	LAST BIRTH<br>NAME ________	NEXT-TO-LAST BIRTH<br>NAME ________	SECOND-FROM-LAST BIRTH<br>NAME ________
534	Where did you seek advice or treatment? **(9)**<br><br>Anywhere else?<br><br>PROBE TO IDENTIFY EACH TYPE OF SOURCE.<br><br>IF UNABLE TO DETERMINE IF PUBLIC OR PRIVATE SECTOR, WRITE THE NAME OF THE PLACE.<br><br>________<br>(NAME OF PLACE(S))	PUBLIC SECTOR<br>GOVT HOSPITAL A<br>GOVT HEALTH CENTER . . . . . B<br>GOVT HEALTH POST . . . . C<br>MOBILE CLINIC D<br>FIELDWORKER E<br>OTHER PUBLIC SECTOR ________ F<br>(SPECIFY)<br><br>PRIVATE MEDICAL SECTOR<br>PVT HOSPITAL/ CLINIC . . . . G<br>PHARMACY . . . H<br>PVT DOCTOR . . . I<br>MOBILE CLINIC J<br>FIELDWORKER K<br>OTHER PRIVATE MED. SECTOR ________ L<br>(SPECIFY)<br><br>OTHER SOURCE<br>SHOP . . . . M<br>TRADITIONAL PRACTITIONER N<br>MARKET . . . . . O<br><br>OTHER ________ X<br>(SPECIFY)	PUBLIC SECTOR<br>GOVT HOSPITAL A<br>GOVT HEALTH CENTER . . . . . B<br>GOVT HEALTH POST . . . . C<br>MOBILE CLINIC D<br>FIELDWORKER E<br>OTHER PUBLIC SECTOR ________ F<br>(SPECIFY)<br><br>PRIVATE MEDICAL SECTOR<br>PVT HOSPITAL/ CLINIC . . . . G<br>PHARMACY . . . H<br>PVT DOCTOR . . . I<br>MOBILE CLINIC J<br>FIELDWORKER K<br>OTHER PRIVATE MED. SECTOR ________ L<br>(SPECIFY)<br><br>OTHER SOURCE<br>SHOP . . . . M<br>TRADITIONAL PRACTITIONER N<br>MARKET . . . . . O<br><br>OTHER ________ X<br>(SPECIFY)	PUBLIC SECTOR<br>GOVT HOSPITAL A<br>GOVT HEALTH CENTER . . . . . B<br>GOVT HEALTH POST . . . . C<br>MOBILE CLINIC D<br>FIELDWORKER E<br>OTHER PUBLIC SECTOR ________ F<br>(SPECIFY)<br><br>PRIVATE MEDICAL SECTOR<br>PVT HOSPITAL/ CLINIC . . . . G<br>PHARMACY . . . H<br>PVT DOCTOR . . . I<br>MOBILE CLINIC J<br>FIELDWORKER K<br>OTHER PRIVATE MED. SECTOR ________ L<br>(SPECIFY)<br><br>OTHER SOURCE<br>SHOP . . . . M<br>TRADITIONAL PRACTITIONER N<br>MARKET . . . . . O<br><br>OTHER ________ X<br>(SPECIFY)
535	CHECK 534:	TWO OR MORE CODES CIRCLED ☐<br>ONLY ONE CODE CIRCLED ☐<br>(SKIP TO 537)	TWO OR MORE CODES CIRCLED ☐<br>ONLY ONE CODE CIRCLED ☐<br>(SKIP TO 537)	TWO OR MORE CODES CIRCLED ☐<br>ONLY ONE CODE CIRCLED ☐<br>(SKIP TO 537)
536	Where did you first seek advice or treatment?<br><br>USE LETTER CODE FROM 534.	FIRST PLACE . . . ☐	FIRST PLACE . . . ☐	FIRST PLACE . . . ☐
537	At any time during the illness, did (NAME) take any drugs for the illness?	YES . . . . 1<br>NO . . . . 2<br>(GO BACK TO 503 IN NEXT COLUMN; OR, IF NO MORE BIRTHS, GO TO 553)<br>DON'T KNOW 8	YES . . . . 1<br>NO . . . . 2<br>(GO BACK TO 503 IN NEXT COLUMN; OR, IF NO MORE BIRTHS, GO TO 553)<br>DON'T KNOW 8	YES . . . . 1<br>NO . . . . 2<br>(GO TO 503 IN NEXT-TO-LAST COLUMN OF NEW QUESTIONNAIRE; OR, IF NO MORE BIRTHS, GO TO 553)<br>DON'T KNOW 8

NO.	QUESTIONS AND FILTERS	LAST BIRTH<br>NAME ________	NEXT-TO-LAST BIRTH<br>NAME ________	SECOND-FROM-LAST BIRTH<br>NAME ________
538	What drugs did (NAME) take? **(13)**<br><br>Any other drugs?<br><br>RECORD ALL MENTIONED.	ANTIMALARIAL DRUGS<br>SP/FANSIDAR . . . A<br>CHLOROQUINE B<br>AMODIAQUINE C<br>QUININE . . . . D<br>COMBINATION WITH ARTEMISININ E<br>OTHER ANTI-MALARIAL ________ . . . F<br>(SPECIFY)<br><br>ANTIBIOTIC DRUGS<br>PILL/SYRUP . . . G<br>INJECTION . . . H<br><br>OTHER DRUGS<br>ASPIRIN . . . . I<br>ACETA-MINOPHEN . . . J<br>IBUPROFEN . . . K<br><br>OTHER ________ X<br>(SPECIFY)<br>DON'T KNOW . . . . . Z	ANTIMALARIAL DRUGS<br>SP/FANSIDAR . . . A<br>CHLOROQUINE . B<br>AMODIAQUINE C<br>QUININE . . . . D<br>COMBINATION WITH ARTEMISININ E<br>OTHER ANTI-MALARIAL ________ . . . F<br>(SPECIFY)<br><br>ANTIBIOTIC DRUGS<br>PILL/SYRUP . . . G<br>INJECTION . . . H<br><br>OTHER DRUGS<br>ASPIRIN . . . . I<br>ACETA-MINOPHEN . . . J<br>IBUPROFEN . . . K<br><br>OTHER ________ X<br>(SPECIFY)<br>DON'T KNOW . . . . . Z	ANTIMALARIAL DRUGS<br>SP/FANSIDAR . . . A<br>CHLOROQUINE B<br>AMODIAQUINE C<br>QUININE . . . . D<br>COMBINATION WITH ARTEMISININ E<br>OTHER ANTI-MALARIAL ________ . . . F<br>(SPECIFY)<br><br>ANTIBIOTIC DRUGS<br>PILL/SYRUP . . . G<br>INJECTION . . . H<br><br>OTHER DRUGS<br>ASPIRIN . . . . I<br>ACETA-MINOPHEN . . . J<br>IBUPROFEN . . . K<br><br>OTHER ________ X<br>(SPECIFY)<br>DON'T KNOW . . . . . Z
539<br>**(12)**	CHECK 538:<br>ANY CODE A-F CIRCLED?	YES ☐ ↓<br>NO ☐ (GO BACK TO 503 IN NEXT COLUMN; OR, IF NO MORE BIRTHS, GO TO 553)	YES ☐ ↓<br>NO ☐ (GO BACK TO 503 IN NEXT COLUMN; OR, IF NO MORE BIRTHS, GO TO 553)	YES ☐ ↓<br>NO ☐ (GO TO 503 IN NEXT-TO-LAST COLUMN OF NEW QUESTIONNAIRE; OR, IF NO MORE BIRTHS, GO TO 553)
540<br>**(12)**	CHECK 538:<br><br>SP/FANSIDAR ('A') GIVEN	CODE 'A' CIRCLED ☐ ↓<br>CODE 'A' NOT CIRCLED ☐ (SKIP TO 542)	CODE 'A' CIRCLED ☐ ↓<br>CODE 'A' NOT CIRCLED ☐ (SKIP TO 542)	CODE 'A' CIRCLED ☐ ↓<br>CODE 'A' NOT CIRCLED ☐ (SKIP TO 542)
541<br>**(12)**	How long after the fever started did (NAME) first take (SP/Fansidar)?	SAME DAY . . . . 0<br>NEXT DAY . . . . 1<br>TWO DAYS AFTER FEVER . . . . 2<br>THREE OR MORE DAYS AFTER FEVER . . . . 3<br>DON'T KNOW . . . 8	SAME DAY . . . . 0<br>NEXT DAY . . . . 1<br>TWO DAYS AFTER FEVER . . . . 2<br>THREE OR MORE DAYS AFTER FEVER . . . . 3<br>DON'T KNOW . . . 8	SAME DAY . . . . 0<br>NEXT DAY . . . . 1<br>TWO DAYS AFTER FEVER . . . . 2<br>THREE OR MORE DAYS AFTER FEVER . . . . 3<br>DON'T KNOW . . . 8

NO.	QUESTIONS AND FILTERS	LAST BIRTH<br>NAME ________	NEXT-TO-LAST BIRTH<br>NAME ________	SECOND-FROM-LAST BIRTH<br>NAME ________
542<br>**(12)**	CHECK 538:<br><br>CHLOROQUINE ('B') GIVEN	CODE 'B' CIRCLED ↓<br>CODE 'B' NOT CIRCLED (SKIP TO 544)	CODE 'B' CIRCLED ↓<br>CODE 'B' NOT CIRCLED (SKIP TO 544)	CODE 'B' CIRCLED ↓<br>CODE 'B' NOT CIRCLED (SKIP TO 544)
543<br>**(12)**	How long after the fever started did (NAME) first take chloroquine?	SAME DAY . . . . 0<br>NEXT DAY . . . . 1<br>TWO DAYS AFTER FEVER . . . . 2<br>THREE OR MORE DAYS AFTER FEVER . . . . 3<br>DON'T KNOW . . . 8	SAME DAY . . . . 0<br>NEXT DAY . . . . 1<br>TWO DAYS AFTER FEVER . . . . 2<br>THREE OR MORE DAYS AFTER FEVER . . . . 3<br>DON'T KNOW . . . 8	SAME DAY . . . . 0<br>NEXT DAY . . . . 1<br>TWO DAYS AFTER FEVER . . . . 2<br>THREE OR MORE DAYS AFTER FEVER . . . . 3<br>DON'T KNOW . . . 8
544<br>**(12)**	CHECK 538:<br><br>AMODIAQUINE ('C') GIVEN	CODE 'C' CIRCLED ↓<br>CODE 'C' NOT CIRCLED (SKIP TO 546)	CODE 'C' CIRCLED ↓<br>CODE 'C' NOT CIRCLED (SKIP TO 546)	CODE 'C' CIRCLED ↓<br>CODE 'C' NOT CIRCLED (SKIP TO 546)
545<br>**(12)**	How long after the fever started did (NAME) first take amodiaquine?	SAME DAY . . . . 0<br>NEXT DAY . . . . 1<br>TWO DAYS AFTER FEVER . . . . 2<br>THREE OR MORE DAYS AFTER FEVER . . . . 3<br>DON'T KNOW . . . 8	SAME DAY . . . . 0<br>NEXT DAY . . . . 1<br>TWO DAYS AFTER FEVER . . . . 2<br>THREE OR MORE DAYS AFTER FEVER . . . . 3<br>DON'T KNOW . . . 8	SAME DAY . . . . 0<br>NEXT DAY . . . . 1<br>TWO DAYS AFTER FEVER . . . . 2<br>THREE OR MORE DAYS AFTER FEVER . . . . 3<br>DON'T KNOW . . . 8
546<br>**(12)**	CHECK 538:<br><br>QUININE ('D') GIVEN	CODE 'D' CIRCLED ↓<br>CODE 'D' NOT CIRCLED (SKIP TO 548)	CODE 'D' CIRCLED ↓<br>CODE 'D' NOT CIRCLED (SKIP TO 548)	CODE 'D' CIRCLED ↓<br>CODE 'D' NOT CIRCLED (SKIP TO 548)
547<br>**(12)**	How long after the fever started did (NAME) first take quinine?	SAME DAY . . . . 0<br>NEXT DAY . . . . 1<br>TWO DAYS AFTER FEVER . . . . 2<br>THREE OR MORE DAYS AFTER FEVER . . . . 3<br>DON'T KNOW . . . 8	SAME DAY . . . . 0<br>NEXT DAY . . . . 1<br>TWO DAYS AFTER FEVER . . . . 2<br>THREE OR MORE DAYS AFTER FEVER . . . . 3<br>DON'T KNOW . . . 8	SAME DAY . . . . 0<br>NEXT DAY . . . . 1<br>TWO DAYS AFTER FEVER . . . . 2<br>THREE OR MORE DAYS AFTER FEVER . . . . 3<br>DON'T KNOW . . . 8
548<br>**(12)**	CHECK 538:<br><br>COMBINATION WITH ARTEMISININ ('E') GIVEN	CODE 'E' CIRCLED ↓<br>CODE 'E' NOT CIRCLED (SKIP TO 550)	CODE 'E' CIRCLED ↓<br>CODE 'E' NOT CIRCLED (SKIP TO 550)	CODE 'E' CIRCLED ↓<br>CODE 'E' NOT CIRCLED (SKIP TO 550)

NO.	QUESTIONS AND FILTERS	LAST BIRTH<br>NAME ________	NEXT-TO-LAST BIRTH<br>NAME ________	SECOND-FROM-LAST BIRTH<br>NAME ________
549<br>**(12)**	How long after the fever started did (NAME) first take (COMBINATION WITH ARTEMISININ)?	SAME DAY . . . . 0<br>NEXT DAY . . . . 1<br>TWO DAYS AFTER<br>FEVER . . . . 2<br>THREE OR MORE<br>DAYS AFTER<br>FEVER . . . . 3<br>DON'T KNOW . . . 8	SAME DAY . . . . 0<br>NEXT DAY . . . . 1<br>TWO DAYS AFTER<br>FEVER . . . . 2<br>THREE OR MORE<br>DAYS AFTER<br>FEVER . . . . 3<br>DON'T KNOW . . . 8	SAME DAY . . . . 0<br>NEXT DAY . . . . 1<br>TWO DAYS AFTER<br>FEVER . . . . 2<br>THREE OR MORE<br>DAYS AFTER<br>FEVER . . . . 3<br>DON'T KNOW . . . 8
550<br>**(12)**	CHECK 538:<br><br>OTHER ANTIMALARIAL ('F') GIVEN	CODE 'F' CIRCLED ☐<br>CODE 'F' NOT CIRCLED ☐ (GO BACK TO 503 IN NEXT COLUMN; OR, IF NO MORE BIRTHS, GO TO 553)	CODE 'F' CIRCLED ☐<br>CODE 'F' NOT CIRCLED ☐ (GO BACK TO 503 IN NEXT COLUMN; OR, IF NO MORE BIRTHS, GO TO 553)	CODE 'F' CIRCLED ☐<br>CODE 'F' NOT CIRCLED ☐ (GO TO 503 IN NEXT-TO-LAST COLUMN OF NEW QUESTIONNAIRE; OR, IF NO MORE BIRTHS, GO TO 553)
551<br>**(12)**	How long after the fever started did (NAME) first take (OTHER ANTIMALARIAL)?	SAME DAY . . . . 0<br>NEXT DAY . . . . 1<br>TWO DAYS AFTER<br>FEVER . . . . 2<br>THREE OR MORE<br>DAYS AFTER<br>FEVER . . . . 3<br>DON'T KNOW . . . 8	SAME DAY . . . . 0<br>NEXT DAY . . . . 1<br>TWO DAYS AFTER<br>FEVER . . . . 2<br>THREE OR MORE<br>DAYS AFTER<br>FEVER . . . . 3<br>DON'T KNOW . . . 8	SAME DAY . . . . 0<br>NEXT DAY . . . . 1<br>TWO DAYS AFTER<br>FEVER . . . . 2<br>THREE OR MORE<br>DAYS AFTER<br>FEVER . . . . 3<br>DON'T KNOW . . . 8
552		GO BACK TO 503 IN NEXT COLUMN; OR, IF NO MORE BIRTHS, GO TO 553.	GO BACK TO 503 IN NEXT COLUMN; OR, IF NO MORE BIRTHS, GO TO 553.	GO TO 503 IN NEXT-TO-LAST COLUMN OF NEW QUESTIONNAIRE; OR, IF NO MORE BIRTHS, GO TO 553.

NO.	QUESTIONS AND FILTERS	CODING CATEGORIES	SKIP
553	CHECK 215 AND 218, ALL ROWS:<br><br>NUMBER OF CHILDREN BORN IN 2005 **(1)** OR LATER LIVING WITH THE RESPONDENT<br><br>ONE OR MORE ☐ NONE ☐<br><br>RECORD NAME OF YOUNGEST CHILD LIVING WITH HER AND CONTINUE WITH 554<br><br>______________________<br>(NAME)		NONE → 556
554	The last time (NAME FROM 553) passed stools, what was done to dispose of the stools?	CHILD USED TOILET OR LATRINE . . . 01<br>PUT/RINSED<br>INTO TOILET OR LATRINE . . . . 02<br>PUT/RINSED<br>INTO DRAIN OR DITCH . . . . 03<br>THROWN INTO GARBAGE . . . . 04<br>BURIED . . . . 05<br>LEFT IN THE OPEN . . . . 06<br>OTHER ______________________ 96<br>(SPECIFY)	
555	CHECK 522(a) AND 522(b), ALL COLUMNS:<br><br>NO CHILD RECEIVED FLUID FROM ORS PACKET OR PRE-PACKAGED ORS LIQUID **(14)** ☐<br><br>ANY CHILD RECEIVED FLUID FROM ORS PACKET OR PRE-PACKAGED ORS LIQUID **(14)** ☐		ANY CHILD → 557
556	Have you ever heard of a special product called [LOCAL NAME FOR ORS PACKET OR PRE-PACKAGED ORS LIQUID] **(14)** you can get for the treatment of diarrhea?	YES . . . . 1<br>NO 2	
557	CHECK 215 AND 218, ALL ROWS:<br><br>NUMBER OF CHILDREN BORN IN 2008 **(15)** OR LATER LIVING WITH THE RESPONDENT<br><br>ONE OR MORE ☐ NONE ☐<br><br>RECORD NAME OF YOUNGEST CHILD LIVING WITH HER AND CONTINUE WITH 558<br><br>______________________<br>(NAME)		NONE → 601

NO.	QUESTIONS AND FILTERS	CODING CATEGORIES	SKIP
558	Now I would like to ask you about liquids or foods that (NAME FROM 557) had yesterday during the day or at night. I am interested in whether your child had the item I mention even if it was combined with other foods. **(16)**<br><br>Did (NAME FROM 557) (drink/eat):	YES NO DK	
	a) Plain water?	**a)** 1 2 8	
	b) Juice or juice drinks?	**b)** 1 2 8	
	c) Clear broth?	**c)** 1 2 8	
	d) Milk such as tinned, powdered, or fresh animal milk?<br>IF YES: How many times did (NAME) drink milk?<br>IF 7 OR MORE TIMES, RECORD '7'.	**d)** 1 2 8<br>NUMBER OF TIMES DRANK MILK ☐	
	e) Infant formula?<br>IF YES: How many times did (NAME) drink infant formula?<br>IF 7 OR MORE TIMES, RECORD '7'.	**e)** 1 2 8<br>NUMBER OF TIMES DRANK FORMULA ☐	
	f) Any other liquids?	**f)** 1 2 8	
	g) Yogurt?<br>IF YES: How many times did (NAME) eat yogurt?<br>IF 7 OR MORE TIMES, RECORD '7'.	**g)** 1 2 8<br>NUMBER OF TIMES ATE YOGURT ☐	
	h) Any [BRAND NAME OF COMMERCIALLY FORTIFIED BABY FOOD, E.G., Cerelac]? **(17)**	**h)** 1 2 8	
	i) Bread, rice, noodles, porridge, or other foods made from grains? **(18)**	**i)** 1 2 8	
	j) Pumpkin, carrots, squash or sweet potatoes that are yellow or orange inside? **(19)**	**j)** 1 2 8	
	k) White potatoes, white yams, manioc, cassava, or any other foods made from roots?	**k)** 1 2 8	
	l) Any dark green, leafy vegetables? **(20)**	**l)** 1 2 8	
	m) Ripe mangoes, papayas or [INSERT ANY OTHER LOCALLY AVAILABLE VITAMIN A-RICH FRUITS]?	**m)** 1 2 8	
	n) Any other fruits or vegetables?	**n)** 1 2 8	
	o) Liver, kidney, heart or other organ meats?	**o)** 1 2 8	
	p) Any meat, such as beef, pork, lamb, goat, chicken, or duck?	**p)** 1 2 8	
	q) Eggs?	**q)** 1 2 8	
	r) Fresh or dried fish or shellfish?	**r)** 1 2 8	
	s) Any foods made from beans, peas, lentils, or nuts?	**s)** 1 2 8	
	t) Cheese or other food made from milk?	**t)** 1 2 8	
	u) Any other solid, semi-solid, or soft food?	**u)** 1 2 8	
559	CHECK 558 (CATEGORIES "g" THROUGH "u"):<br>NOT A SINGLE "YES" ☐ ↓	AT LEAST ONE "YES" ☐ →	561

NO.	QUESTIONS AND FILTERS	CODING CATEGORIES	SKIP
560	Did (NAME) eat any solid, semi-solid, or soft foods yesterday during the day or at night?<br><br>IF 'YES' PROBE: What kind of solid, semi-solid or soft foods did (NAME) eat?	YES . . . . 1<br>(GO BACK TO 558 TO RECORD FOOD EATEN YESTERDAY)<br><br>NO 2	 <br><br><br>→ 601
561	How many times did (NAME FROM 557) eat solid, semi-solid, or soft foods yesterday during the day or at night?<br><br>IF 7 OR MORE TIMES, RECORD '7'.	NUMBER OF TIMES . . . . [ ]<br><br>DON'T KNOW 8	

SECTION 5 FOOTNOTES

(1) Year of fieldwork is assumed to be 2010. For fieldwork beginning in 2011 or 2012, the year should be 2006 or 2007, respectively.
(2) To be developed locally since immunization practices may vary from country to country, as may the terms used for the written record and for the vaccinations. Add yellow fever, rubella, MMR, Hib (3 doses), and hepatitis B (3 doses) in Q. 506 in countries where these vaccinations are listed on the vaccination card.
(3) Filter should reflect the vaccination list in Q. 506.
(4) To be developed locally since immunization practices may vary from country to country, as may the terms used for the vaccinations. Include question on pentavalent injection or injections for yellow fever, rubella, MMR, Hib, and Hepatitis B where these are included in Q. 506.
(5) Adapt question locally after determining the most common injection site.
(6) Delete this question in countries where Polio 0 is not part of the immunization schedule.
(7) Adapt question locally, some countries do not give measles vaccination until 12-15 months of age.
(8) The term(s) used for diarrhea should encompass the expressions used for all forms of diarrhea, including bloody stools (consistent with dysentery), watery stools, etc.
(9) Coding categories to be developed locally and revised based on the pretest; however, the broad categories must be maintained.
(10) Include in the question the common names/brands for pre-packaged ORS liquids. If pre-packaged ORS liquids are not available in the country, this item should be deleted.
(11) This item should be adapted to include the terms used locally for the recommended home fluid. The ingredients promoted by the government for making the recommended home fluid should be reflected in the category. If the government does not recommend a homemade fluid, then the word "government" should be dropped from the question.
(12) The question should be deleted in countries that are not affected by malaria.
(13) Coding categories to be developed locally and revised based on the pretest. All antimalarials commonly used in the country should be included in the response categories. Common brand names of drugs, such as Bayer, Tylenol or Paracetamol, should be added to the response categories for aspirin, acetaminophen, or ibuprofen as appropriate.
(14) Delete "OR PRE-PACKAGED ORS LIQUID" in countries where such liquid is not available.
(15) Year of fieldwork is assumed to be 2010. For fieldwork beginning in 2011 or 2012, the year should be 2009 or 2010, respectively.
(16) A separate category: "Foods made with red palm oil, palm nut, or palm nut pulp sauce" must be added in countries where these items are consumed. A separate category: "Grubs, snails, insects or other small protein food" must be added in countries where these items are eaten. Items in each food group should be modified to include only those foods that are locally available and/or consumed in the country. Local terms should be used.
(17) In the case of fortified foods, the interviewer should ask to see the package and/or brand label (if available), to confirm that the food is fortified.
(18) Grains include millet, sorghum, maize, rice, wheat, or other local grains. Start with local foods, e.g. ugali, nshima, fufu, chapati, then follow with bread, rice, noodles, etc.
(19) Items in this category should be modified to include only vitamin A rich tubers, starches, or red, orange, or yellow vegetables that are consumed in the country.
(20) These include cassava leaves, bean leaves, kale, spinach, pepper leaves, taro leaves, amaranth leaves, or other dark green, leafy vegetables.

SECTION 6. MARRIAGE AND SEXUAL ACTIVITY

NO.	QUESTIONS AND FILTERS	CODING CATEGORIES	SKIP
601	Are you currently married or living together with a man as if married?	YES, CURRENTLY MARRIED . . . . 1<br>YES, LIVING WITH A MAN . . . . 2<br>NO, NOT IN UNION 3	1, 2 → 604
602	Have you ever been married or lived together with a man as if married?	YES, FORMERLY MARRIED . . . . 1<br>YES, LIVED WITH A MAN . . . . 2<br>NO 3	3 → 612
603	What is your marital status now: are you widowed, divorced, or separated?	WIDOWED . . . . 1<br>DIVORCED . . . . 2<br>SEPARATED 3	→ 609
604	Is your (husband/partner) living with you now or is he staying elsewhere?	LIVING WITH HER . . . . 1<br>STAYING ELSEWHERE 2	
605	RECORD THE HUSBAND'S/PARTNER'S NAME AND LINE NUMBER FROM THE HOUSEHOLD QUESTIONNAIRE.<br>IF HE IS NOT LISTED IN THE HOUSEHOLD, RECORD '00'.	NAME ____________________<br>LINE NO. . . . . [ ][ ]	
606<br>**(1)**	Does your (husband/partner) have other wives or does he live with other women as if married?	YES . . . . 1<br>NO . . . . 2<br>DON'T KNOW 8	2, 8 → 609
607<br>**(1)**	Including yourself, in total, how many wives or live-in partners does he have?	TOTAL NUMBER OF WIVES AND LIVE-IN PARTNERS . . . . . [ ][ ]<br>DON'T KNOW 98	
608<br>**(1)**	Are you the first, second, ... wife?	RANK . . . . [ ][ ]	
609	Have you been married or lived with a man only once or more than once?	ONLY ONCE . . . . 1<br>MORE THAN ONCE 2	
610	CHECK 609:<br>MARRIED/ LIVED WITH A MAN ONLY ONCE [ ] ↓<br>In what month and year did you start living with your (husband/partner)?<br>MARRIED/ LIVED WITH A MAN MORE THAN ONCE [ ] ↓<br>Now I would like to ask about your first (husband/partner). In what month and year did you start living with him?	MONTH . . . . [ ][ ]<br>DON'T KNOW MONTH . . . . 98<br>YEAR . . . . [ ][ ][ ][ ]<br>DON'T KNOW YEAR 9998	YEAR → 612
611	How old were you when you first started living with him?	AGE . . . . [ ][ ]	
612	CHECK FOR THE PRESENCE OF OTHERS. BEFORE CONTINUING, MAKE EVERY EFFORT TO ENSURE PRIVACY.		
613	Now I would like to ask some questions about sexual activity in order to gain a better understanding of some important life issues.<br>How old were you when you had sexual intercourse for the very first time?	NEVER HAD SEXUAL INTERCOURSE . . . . .00<br>AGE IN YEARS . . . . [ ][ ]<br>FIRST TIME WHEN STARTED LIVING WITH (FIRST) HUSBAND/PARTNER 95	00 → 628

NO.	QUESTIONS AND FILTERS	CODING CATEGORIES	SKIP
614	Now I would like to ask you some questions about your recent sexual activity. Let me assure you again that your answers are completely confidential and will not be told to anyone. If we should come to any question that you don't want to answer, just let me know and we will go to the next question.		
615	When was the last time you had sexual intercourse?<br><br>IF LESS THAN 12 MONTHS, ANSWER MUST BE RECORDED IN DAYS, WEEKS OR MONTHS.<br>IF 12 MONTHS (ONE YEAR) OR MORE, ANSWER MUST BE RECORDED IN YEARS.	DAYS AGO . . . . 1<br>WEEKS AGO . . . . 2<br>MONTHS AGO . . . . 3<br>YEARS AGO 4	 <br><br>→ 627

		LAST SEXUAL PARTNER	SECOND-TO-LAST SEXUAL PARTNER	THIRD-TO-LAST SEXUAL PARTNER
616	When was the last time you had sexual intercourse with this person?		DAYS AGO 1<br>WEEKS AGO 2<br>MONTHS AGO 3	DAYS AGO 1<br>WEEKS AGO 2<br>MONTHS AGO 3
617	The last time you had sexual intercourse (with this second/third person), was a condom used? **(2)**	YES . . . . 1<br>NO . . . . 2<br>(SKIP TO 619)	YES . . . . 1<br>NO . . . . 2<br>(SKIP TO 619)	YES . . . . 1<br>NO . . . . 2<br>(SKIP TO 619)
618	Was a condom used every time you had sexual intercourse with this person in the last 12 months?	YES . . . . 1<br>NO 2	YES . . . . 1<br>NO 2	YES . . . . 1<br>NO 2
619	What was your relationship to this person with whom you had sexual intercourse?<br><br>IF BOYFRIEND:<br>Were you living together as if married?<br>IF YES, CIRCLE '2'.<br>IF NO, CIRCLE '3'.	HUSBAND . . . . 1<br>LIVE-IN PARTNER . . . 2<br>BOYFRIEND NOT LIVING WITH RESPONDENT . . . 3<br>CASUAL ACQUAINTANCE . . . 4<br>CLIENT/PROSTITUTE 5<br>OTHER ____________ 6<br>(SPECIFY)<br>(SKIP TO 622)	HUSBAND . . . . 1<br>LIVE-IN PARTNER . . . 2<br>BOYFRIEND NOT LIVING WITH RESPONDENT . . . 3<br>CASUAL ACQUAINTANCE . . . 4<br>CLIENT/PROSTITUTE 5<br>OTHER ____________ 6<br>(SPECIFY)<br>(SKIP TO 622)	HUSBAND . . . . 1<br>LIVE-IN PARTNER . . . 2<br>BOYFRIEND NOT LIVING WITH RESPONDENT . . . 3<br>CASUAL ACQUAINTANCE . . . 4<br>CLIENT/PROSTITUTE 5<br>OTHER ____________ 6<br>(SPECIFY)<br>(SKIP TO 622)
620	CHECK 609:	MARRIED ONLY ONCE<br>MARRIED MORE THAN ONCE (SKIP TO 622)	MARRIED ONLY ONCE<br>MARRIED MORE THAN ONCE (SKIP TO 622)	MARRIED ONLY ONCE<br>MARRIED MORE THAN ONCE (SKIP TO 622)
621	CHECK 613:	FIRST TIME WHEN STARTED LIVING WITH FIRST HUSBAND (SKIP TO 623)<br>OTHER	FIRST TIME WHEN STARTED LIVING WITH FIRST HUSBAND (SKIP TO 623)<br>OTHER	FIRST TIME WHEN STARTED LIVING WITH FIRST HUSBAND (SKIP TO 623)<br>OTHER
622	How long ago did you first have sexual intercourse with this (second/third) person?	DAYS AGO 1<br>WEEKS AGO 2<br>MONTHS AGO 3<br>YEARS AGO 4	DAYS AGO 1<br>WEEKS AGO 2<br>MONTHS AGO 3<br>YEARS AGO 4	DAYS AGO 1<br>WEEKS AGO 2<br>MONTHS AGO 3<br>YEARS AGO 4
623	How many times during the last 12 months did you have sexual intercourse with this person?<br>IF NON-NUMERIC ANSWER, PROBE TO GET AN ESTIMATE.<br>IF NUMBER OF TIMES IS 95 OR MORE, WRITE '95'.	NUMBER OF TIMES	NUMBER OF TIMES	NUMBER OF TIMES
624	How old is this person?	AGE OF PARTNER<br>DON'T KNOW 98	AGE OF PARTNER<br>DON'T KNOW 98	AGE OF PARTNER<br>DON'T KNOW 98
625	Apart from (this person/these two people), have you had sexual intercourse with any other person in the last 12 months?	YES . . . . 1<br>(GO BACK TO 616 IN NEXT COLUMN)<br>NO . . . . 2<br>(SKIP TO 627)	YES . . . . 1<br>(GO BACK TO 616 IN NEXT COLUMN)<br>NO . . . . 2<br>(SKIP TO 627)	
626	In total, with how many different people have you had sexual intercourse in the last 12 months?<br>IF NON-NUMERIC ANSWER, PROBE TO GET AN ESTIMATE.<br>IF NUMBER OF PARTNERS IS 95 OR MORE, WRITE '95'.			NUMBER OF PARTNERS LAST 12 MONTHS . . .<br>DON'T KNOW . . . 98

NO.	QUESTIONS AND FILTERS	CODING CATEGORIES	SKIP
627	In total, with how many different people have you had sexual intercourse in your lifetime?<br><br>IF NON-NUMERIC ANSWER, PROBE TO GET AN ESTIMATE.<br><br>IF NUMBER OF PARTNERS IS 95 OR MORE, WRITE '95'.	NUMBER OF PARTNERS IN LIFETIME . . . . [ ][ ]<br><br>DON'T KNOW 98	
628	PRESENCE OF OTHERS DURING THIS SECTION	YES NO<br>CHILDREN <10 . . . . 1 2<br>MALE ADULTS . . . . 1 2<br>FEMALE ADULTS 1 2	
629	Do you know of a place where a person can get condoms?	YES . . . . 1<br>NO 2	 → 632
630	Where is that? **(3)**<br><br>Any other place?<br><br>PROBE TO IDENTIFY EACH TYPE OF SOURCE.<br><br>IF UNABLE TO DETERMINE IF PUBLIC OR PRIVATE SECTOR, WRITE THE NAME OF THE PLACE.<br><br>____________________<br>(NAME OF PLACE(S))	PUBLIC SECTOR<br>GOVERNMENT HOSPITAL . . . . A<br>GOVT. HEALTH CENTER . . . . B<br>FAMILY PLANNING CLINIC . . . . C<br>MOBILE CLINIC . . . . D<br>FIELDWORKER . . . . E<br>OTHER PUBLIC SECTOR __________ F<br>(SPECIFY)<br><br>PRIVATE MEDICAL SECTOR<br>PRIVATE HOSPITAL/CLINIC . . . . . G<br>PHARMACY . . . . H<br>PRIVATE DOCTOR . . . . I<br>MOBILE CLINIC . . . . J<br>FIELDWORKER . . . . K<br>OTHER PRIVATE MEDICAL SECTOR __________ L<br>(SPECIFY)<br><br>OTHER SOURCE<br>SHOP . . . . M<br>CHURCH . . . . N<br>FRIENDS/RELATIVES . . . . O<br><br>OTHER __________ X<br>(SPECIFY)	
631	If you wanted to, could you yourself get a condom?	YES . . . . 1<br>NO . . . . 2<br>DON'T KNOW/UNSURE 8	
632<br>**(4)**	Do you know of a place where a person can get female condoms?	YES . . . . 1<br>NO 2	 → 701

NO.	QUESTIONS AND FILTERS	CODING CATEGORIES		SKIP
633 **(4)**	Where is that? **(3)**<br><br>Any other place?<br><br>PROBE TO IDENTIFY EACH TYPE OF SOURCE.<br><br>IF UNABLE TO DETERMINE IF PUBLIC OR PRIVATE SECTOR, WRITE THE NAME OF THE PLACE.<br><br>____________________<br>(NAME OF PLACE(S))	PUBLIC SECTOR		
		GOVERNMENT HOSPITAL	A	
		GOVT. HEALTH CENTER	B	
		FAMILY PLANNING CLINIC	C	
		MOBILE CLINIC	D	
		FIELDWORKER	E	
		OTHER PUBLIC SECTOR __________ (SPECIFY)	F	
		PRIVATE MEDICAL SECTOR		
		PRIVATE HOSPITAL/CLINIC	G	
		PHARMACY	H	
		PRIVATE DOCTOR	I	
		MOBILE CLINIC	J	
		FIELDWORKER	K	
		OTHER PRIVATE MEDICAL SECTOR __________ (SPECIFY)	L	
		OTHER SOURCE		
		SHOP	M	
		CHURCH	N	
		FRIENDS/RELATIVES	O	
		OTHER __________ (SPECIFY)	X	
634 **(4)**	If you wanted to, could you yourself get a female condom?	YES	1	
		NO	2	
		DON'T KNOW/UNSURE	8	

(1) The question should be deleted in countries where polygyny is not practiced.
(2) In countries with an active female condom program, the wording of the question should be modified to include reference to both the male and female condom.
(3) Coding categories to be developed locally and revised based on the pretest; however, the broad categories must be maintained.
(4) The question should be deleted in countries where female condoms are not actively promoted.

SECTION 7. FERTILITY PREFERENCES

NO.	QUESTIONS AND FILTERS	CODING CATEGORIES	SKIP
701	CHECK 304:<br>NEITHER STERILIZED ☐ ↓ HE OR SHE STERILIZED ☐ → 712		712
702	CHECK 226:<br>PREGNANT ☐ ↓ NOT PREGNANT OR UNSURE ☐ → 704		704
703	Now I have some questions about the future. After the child you are expecting now, would you like to have another child, or would you prefer not to have any more children?	HAVE ANOTHER CHILD . . . . 1<br>NO MORE . . . . 2<br>UNDECIDED/DON'T KNOW 8	→ 705<br><br>→ 711 (2, 8)
704	Now I have some questions about the future. Would you like to have (a/another) child, or would you prefer not to have any (more) children?	HAVE (A/ANOTHER) CHILD . . . . 1<br>NO MORE/NONE . . . . 2<br>SAYS SHE CAN'T GET PREGNANT 3<br>UNDECIDED/DON'T KNOW 8	 → 707<br>→ 712<br>→ 710
705	CHECK 226:<br>NOT PREGNANT OR UNSURE ☐ ↓<br>How long would you like to wait from now before the birth of (a/another) child?<br>PREGNANT ☐ ↓<br>After the birth of the child you are expecting now, how long would you like to wait before the birth of another child?	MONTHS . . . . 1 ☐☐<br>YEARS . . . . 2 ☐☐<br>SOON/NOW . . . . 993<br>SAYS SHE CAN'T GET PREGNANT 994<br>AFTER MARRIAGE . . . . 995<br>OTHER ______________________ 996<br>(SPECIFY)<br>DON'T KNOW 998	 <br>→ 710<br>→ 712<br><br>→ 710 (995, 996, 998)
706	CHECK 226:<br>NOT PREGNANT OR UNSURE ☐ ↓ PREGNANT ☐ → 711		711
707	CHECK 303: USING A CONTRACEPTIVE METHOD?<br>NOT CURRENTLY USING ☐ ↓ CURRENTLY USING ☐ → 712		712
708	CHECK 705:<br>NOT ASKED ☐ ↓ 24 OR MORE MONTHS OR 02 OR MORE YEARS ☐ ↓ 00-23 MONTHS OR 00-01 YEAR ☐ → 711		711

NO.	QUESTIONS AND FILTERS	CODING CATEGORIES	SKIP
709	CHECK 704:<br><br>WANTS TO HAVE A/ANOTHER CHILD ☐<br>You have said that you do not want (a/another) child soon.<br>Can you tell me why you are not using a method to prevent pregnancy?<br>Any other reason?<br><br>WANTS NO MORE/ NONE ☐<br>You have said that you do not want any (more) children.<br>Can you tell me why you are not using a method to prevent pregnancy?<br>Any other reason?<br><br>RECORD ALL REASONS MENTIONED.	NOT MARRIED . . . . A<br><br>FERTILITY-RELATED REASONS<br>NOT HAVING SEX . . . . B<br>INFREQUENT SEX . . . . C<br>MENOPAUSAL/HYSTERECTOMY D<br>CAN'T GET PREGNANT . . . . E<br>NOT MENSTRUATED SINCE LAST BIRTH . . . . F<br>BREASTFEEDING . . . . G<br>UP TO GOD/FATALISTIC . . . . H<br><br>OPPOSITION TO USE<br>RESPONDENT OPPOSED . . . . I<br>HUSBAND/PARTNER OPPOSED . . . J<br>OTHERS OPPOSED . . . . K<br>RELIGIOUS PROHIBITION . . . . L<br><br>LACK OF KNOWLEDGE<br>KNOWS NO METHOD . . . . M<br>KNOWS NO SOURCE . . . . N<br><br>METHOD-RELATED REASONS<br>SIDE EFFECTS/HEALTH CONCERNS . . . . O<br>LACK OF ACCESS/TOO FAR . . . . . P<br>COSTS TOO MUCH . . . . Q<br>PREFERRED METHOD NOT AVAILABLE . . . . R<br>NO METHOD AVAILABLE . . . . S<br>INCONVENIENT TO USE . . . . T<br>INTERFERES WITH BODY'S NORMAL PROCESSES . . . . U<br><br>OTHER ______________________ X<br>(SPECIFY)<br>DON'T KNOW . . . . Z	
710	CHECK 303: USING A CONTRACEPTIVE METHOD?<br><br>NOT ASKED ☐ NO, NOT CURRENTLY USING ☐ YES, CURRENTLY USING ☐		→ 712
711	Do you think you will use a contraceptive method to delay or avoid pregnancy at any time in the future?	YES . . . . 1<br>NO . . . . 2<br>DON'T KNOW 8	
712	CHECK 216:<br><br>HAS LIVING CHILDREN ☐<br>If you could go back to the time you did not have any children and could choose exactly the number of children to have in your whole life, how many would that be?<br><br>NO LIVING CHILDREN ☐<br>If you could choose exactly the number of children to have in your whole life, how many would that be?<br><br>PROBE FOR A NUMERIC RESPONSE.	NONE . . . . 00<br><br>NUMBER . . . . ☐☐<br><br>OTHER ______________________ 96<br>(SPECIFY)	→ 714<br><br><br><br>→ 714

NO.	QUESTIONS AND FILTERS	CODING CATEGORIES	SKIP
713	How many of these children would you like to be boys, how many would you like to be girls and for how many would it not matter if it's a boy or a girl?	BOYS GIRLS EITHER<br>NUMBER [ ][ ] [ ][ ] [ ][ ]<br>OTHER ______ 96<br>(SPECIFY)	
714	In the last few months have you:<br>Heard about family planning on the radio?<br>Seen anything about family planning on the television?<br>Read about family planning in a newspaper or magazine?	YES NO<br>RADIO . . . . 1 2<br>TELEVISION . . . . 1 2<br>NEWSPAPER OR MAGAZINE . . . 1 2	
715	COUNTRY-SPECIFIC QUESTIONS ON MEDIA MESSAGES ABOUT FAMILY PLANNING.		
716	CHECK 601:<br>YES, CURRENTLY MARRIED [ ]<br>YES, LIVING WITH A MAN [ ]<br>NO, NOT IN UNION [ ]		801
717	CHECK 303: USING A CONTRACEPTIVE METHOD?<br>CURRENTLY USING [ ]<br>NOT CURRENTLY USING OR NOT ASKED [ ]		720
718	Would you say that using contraception is mainly your decision, mainly your (husband's/partner's) decision, or did you both decide together?	MAINLY RESPONDENT . . . . 1<br>MAINLY HUSBAND/PARTNER . . . . . 2<br>JOINT DECISION . . . . 3<br>OTHER ______ 6<br>(SPECIFY)	
719	CHECK 304:<br>NEITHER STERILIZED [ ]<br>HE OR SHE STERILIZED [ ]		801
720	Does your (husband/partner) want the same number of children that you want, or does he want more or fewer than you want?	SAME NUMBER . . . . 1<br>MORE CHILDREN . . . . 2<br>FEWER CHILDREN . . . . 3<br>DON'T KNOW 8	

SECTION 8. HUSBAND'S BACKGROUND AND WOMAN'S WORK

NO.	QUESTIONS AND FILTERS	CODING CATEGORIES	SKIP
801	CHECK 601 AND 602:<br>CURRENTLY MARRIED/ LIVING WITH A MAN ☐ ↓<br>FORMERLY MARRIED/ LIVED WITH A MAN ☐ → 803<br>NEVER MARRIED AND NEVER LIVED WITH A MAN ☐ → 807		→ 803<br>→ 807
802	How old was your (husband/partner) on his last birthday?	AGE IN COMPLETED YEARS ☐☐	
803	Did your (last) (husband/partner) ever attend school?	YES . . . . 1<br>NO 2	 → 806
804	What was the highest level of school he attended: primary, secondary, or higher? **(1)**	PRIMARY . . . . 1<br>SECONDARY . . . . 2<br>HIGHER . . . . 3<br>DON'T KNOW 8	 <br><br>→ 806
805	What was the highest (grade/form/year) he completed at that level? **(1)**<br>IF COMPLETED LESS THAN ONE YEAR AT THAT LEVEL, RECORD '00'.	GRADE . . . . ☐☐<br>DON'T KNOW 98	
806	CHECK 801:<br>CURRENTLY MARRIED/ LIVING WITH A MAN ☐ ↓<br>What is your (husband's/ partner's) occupation? That is, what kind of work does he mainly do?<br>FORMERLY MARRIED/ LIVED WITH A MAN ☐ ↓<br>What was your (last) (husband's/ partner's) occupation? That is, what kind of work did he mainly do?	____________ ☐☐<br>____________<br>____________	
807	Aside from your own housework, have you done any work in the last seven days?	YES . . . . 1<br>NO 2	→ 811
808	As you know, some women take up jobs for which they are paid in cash or kind. Others sell things, have a small business or work on the family farm or in the family business.<br>In the last seven days, have you done any of these things or any other work?	YES . . . . 1<br>NO 2	→ 811
809	Although you did not work in the last seven days, do you have any job or business from which you were absent for leave, illness, vacation, maternity leave, or any other such reason?	YES . . . . 1<br>NO 2	→ 811
810	Have you done any work in the last 12 months?	YES . . . . 1<br>NO 2	 → 815
811	What is your occupation, that is, what kind of work do you mainly do?	____________ ☐☐<br>____________<br>____________	
812	Do you do this work for a member of your family, for someone else, or are you self-employed?	FOR FAMILY MEMBER . . . . 1<br>FOR SOMEONE ELSE . . . . 2<br>SELF-EMPLOYED 3	

NO.	QUESTIONS AND FILTERS	CODING CATEGORIES	SKIP
813	Do you usually work throughout the year, or do you work seasonally, or only once in a while?	THROUGHOUT THE YEAR . . . . 1<br>SEASONALLY/PART OF THE YEAR 2<br>ONCE IN A WHILE 3	
814	Are you paid in cash or kind for this work or are you not paid at all?	CASH ONLY . . . . 1<br>CASH AND KIND . . . . 2<br>IN KIND ONLY . . . . 3<br>NOT PAID 4	
815	CHECK 601:<br>CURRENTLY MARRIED/LIVING WITH A MAN ☐ ↓<br>NOT IN UNION ☐		823
816	CHECK 814:<br>CODE 1 OR 2 CIRCLED ☐ ↓<br>OTHER ☐		819
817	Who usually decides how the money you earn will be used: you, your (husband/partner), or you and your (husband/partner) jointly?	RESPONDENT . . . . 1<br>HUSBAND/PARTNER . . . . 2<br>RESPONDENT AND<br>HUSBAND/PARTNER JOINTLY . . . 3<br>OTHER ______________ 6<br>(SPECIFY)	
818	Would you say that the money that you earn is more than what your (husband/partner) earns, less than what he earns, or about the same?	MORE THAN HIM . . . . 1<br>LESS THAN HIM . . . . 2<br>ABOUT THE SAME . . . . 3<br>HUSBAND/PARTNER HAS<br>NO EARNINGS . . . . 4<br>DON'T KNOW 8	 <br><br><br>→ 820
819	Who usually decides how your (husband's/partner's) earnings will be used: you, your (husband/partner), or you and your (husband/partner) jointly?	RESPONDENT . . . . 1<br>HUSBAND/PARTNER . . . . 2<br>RESPONDENT AND<br>HUSBAND/PARTNER JOINTLY . . . 3<br>HUSBAND/PARTNER HAS<br>NO EARNINGS . . . . 4<br>OTHER ______________ 6<br>(SPECIFY)	
820	Who usually makes decisions about health care for yourself: you, your (husband/partner), you and your (husband/partner) jointly, or someone else?	RESPONDENT . . . . 1<br>HUSBAND/PARTNER . . . . 2<br>RESPONDENT AND<br>HUSBAND/PARTNER JOINTLY . . . 3<br>SOMEONE ELSE . . . . 4<br>OTHER 6	
821	Who usually makes decisions about making major household purchases?	RESPONDENT . . . . 1<br>HUSBAND/PARTNER . . . . 2<br>RESPONDENT AND<br>HUSBAND/PARTNER JOINTLY . . . 3<br>SOMEONE ELSE . . . . 4<br>OTHER 6	
822	Who usually makes decisions about visits to your family or relatives?	RESPONDENT . . . . 1<br>HUSBAND/PARTNER . . . . 2<br>RESPONDENT AND<br>HUSBAND/PARTNER JOINTLY . . . 3<br>SOMEONE ELSE . . . . 4<br>OTHER 6	

NO.	QUESTIONS AND FILTERS	CODING CATEGORIES	SKIP
823	Do you own this or any other house either alone or jointly with someone else?	ALONE ONLY . . . . 1<br>JOINTLY ONLY . . . . 2<br>BOTH ALONE AND JOINTLY . . . . 3<br>DOES NOT OWN 4	
824	Do you own any land either alone or jointly with someone else?	ALONE ONLY . . . . 1<br>JOINTLY ONLY . . . . 2<br>BOTH ALONE AND JOINTLY . . . . 3<br>DOES NOT OWN 4	
825	PRESENCE OF OTHERS AT THIS POINT (PRESENT AND LISTENING, PRESENT BUT NOT LISTENING, OR NOT PRESENT)	PRES./LISTEN. / PRES./NOT LISTEN. / NOT PRES.<br>CHILDREN < 10 . . . . . 1 2 3<br>HUSBAND . . . . 1 2 3<br>OTHER MALES . . . . . 1 2 3<br>OTHER FEMALES . . . 1 2 3	
826	In your opinion, is a husband justified in hitting or beating his wife in the following situations:<br><br>If she goes out without telling him?<br>If she neglects the children?<br>If she argues with him?<br>If she refuses to have sex with him?<br>If she burns the food?	YES / NO / DK<br><br>GOES OUT . . . . 1 2 8<br>NEGL. CHILDREN . . . 1 2 8<br>ARGUES . . . . 1 2 8<br>REFUSES SEX . . . . . 1 2 8<br>BURNS FOOD 1 2 8	

(1) Revise according to the local educational system.

SECTION 9. HIV/AIDS

NO.	QUESTIONS AND FILTERS	CODING CATEGORIES	SKIP
901	Now I would like to talk about something else. Have you ever heard of an illness called AIDS?	YES . . . . 1<br>NO 2	 → 937
902	Can people reduce their chance of getting the AIDS virus by having just one uninfected sex partner who has no other sex partners?	YES . . . . 1<br>NO . . . . 2<br>DON'T KNOW 8	
903 **(1)**	Can people get the AIDS virus from mosquito bites?	YES . . . . 1<br>NO . . . . 2<br>DON'T KNOW 8	
904	Can people reduce their chance of getting the AIDS virus by using a condom every time they have sex?	YES . . . . 1<br>NO . . . . 2<br>DON'T KNOW 8	
905 **(1)**	Can people get the AIDS virus by sharing food with a person who has AIDS?	YES . . . . 1<br>NO . . . . 2<br>DON'T KNOW 8	
906 **(1)**	Can people get the AIDS virus because of witchcraft or other supernatural means?	YES . . . . 1<br>NO . . . . 2<br>DON'T KNOW 8	
907	Is it possible for a healthy-looking person to have the AIDS virus?	YES . . . . 1<br>NO . . . . 2<br>DON'T KNOW 8	
908	Can the virus that causes AIDS be transmitted from a mother to her baby:<br>During pregnancy?<br>During delivery?<br>By breastfeeding?	YES NO DK<br>DURING PREG. . . . . . 1 2 8<br>DURING DELIVERY . . . 1 2 8<br>BREASTFEEDING . . . 1 2 8	
909	CHECK 908:<br>AT LEAST ONE 'YES' ☐ ↓	OTHER ☐	→ 911
910	Are there any special drugs that a doctor or a nurse can give to a woman infected with the AIDS virus to reduce the risk of transmission to the baby?	YES . . . . 1<br>NO . . . . 2<br>DON'T KNOW 8	
911 **(2)**	CHECK 208 AND 215:<br>LAST BIRTH SINCE JANUARY 2008 **(3)** ☐ ↓	NO BIRTHS ☐<br>LAST BIRTH BEFORE JANUARY 2008 **(3)** ☐	→ 926<br>→ 926
912 **(2)**	CHECK 408 FOR LAST BIRTH:<br>HAD ANTENATAL CARE ☐ ↓	NO ANTENATAL CARE ☐	→ 920
913 **(2)**	CHECK FOR PRESENCE OF OTHERS. BEFORE CONTINUING, MAKE EVERY EFFORT TO ENSURE PRIVACY.		
914 **(2)**	During any of the antenatal visits for your last birth were you given any information about:<br>Babies getting the AIDS virus from their mother?<br>Things that you can do to prevent getting the AIDS virus?<br>Getting tested for the AIDS virus?	YES NO DK<br>AIDS FROM MOTHER 1 2 8<br>THINGS TO DO 1 2 8<br>TESTED FOR AIDS 1 2 8	

NO.	QUESTIONS AND FILTERS	CODING CATEGORIES	SKIP
915 **(2)**	Were you offered a test for the AIDS virus as part of your antenatal care?	YES . . . . . 1<br>NO 2	
916 **(2)**	I don't want to know the results, but were you tested for the AIDS virus as part of your antenatal care?	YES . . . . . 1<br>NO 2	 2 → 920
917 **(2)**	Where was the test done? **(4)**<br><br>PROBE TO IDENTIFY THE TYPE OF SOURCE.<br><br>IF UNABLE TO DETERMINE IF PUBLIC OR PRIVATE SECTOR, WRITE THE NAME OF THE PLACE.<br><br>________________<br>(NAME OF PLACE)	PUBLIC SECTOR<br>GOVERNMENT HOSPITAL . . . . . 11<br>GOVT. HEALTH CENTER . . . . . 12<br>STAND-ALONE VCT CENTER . . . 13<br>FAMILY PLANNING CLINIC . . . . . 14<br>MOBILE CLINIC . . . . . 15<br>FIELDWORKER . . . . . 16<br>SCHOOL BASED CLINIC . . . . . 17<br>OTHER PUBLIC SECTOR ________ 18<br>(SPECIFY)<br>PRIVATE MEDICAL SECTOR<br>PRIVATE HOSPITAL/CLINIC/ PRIVATE DOCTOR . . . . . 21<br>STAND-ALONE VCT CENTER . . . . . 22<br>PHARMACY . . . . . 23<br>MOBILE CLINIC . . . . . 24<br>FIELDWORKER . . . . . 25<br>SCHOOL BASED CLINIC . . . . . 26<br>OTHER PRIVATE MEDICAL SECTOR<br>________ 27<br>(SPECIFY)<br>OTHER SOURCE<br>HOME . . . . . 31<br>CORRECTIONAL FACILITY . . . . . 32<br><br>OTHER ________ 96<br>(SPECIFY)	
918 **(2)**	I don't want to know the results, but did you get the results of the test?	YES . . . . . 1<br>NO 2	 2 → 924
919 **(2)**	All women are supposed to receive counseling after being tested. After you were tested, did you receive counseling?	YES . . . . . 1<br>NO . . . . . 2<br>DON'T KNOW 8	1, 2, 8 → 924
920 **(2)**	CHECK 434 FOR LAST BIRTH:<br>ANY CODE 21-36 CIRCLED ☐ ↓	OTHER ☐	→ 926
921 **(2)**	Between the time you went for delivery but before the baby was born, were you offered a test for the AIDS virus?	YES . . . . . 1<br>NO 2	
922 **(2)**	I don't want to know the results, but were you tested for the AIDS virus at that time?	YES . . . . . 1<br>NO 2	 2 → 926
923 **(2)**	I don't want to know the results, but did you get the results of the test?	YES . . . . . 1<br>NO 2	
924 **(2)**	Have you been tested for the AIDS virus since that time you were tested during your pregnancy?	YES . . . . . 1<br>NO 2	1 → 927
925 **(2)**	How many months ago was your most recent HIV test?	MONTHS AGO . . . . . ☐☐<br><br>TWO OR MORE YEARS 95	→ 932

NO.	QUESTIONS AND FILTERS	CODING CATEGORIES	SKIP
926	I don't want to know the results, but have you ever been tested to see if you have the AIDS virus?	YES . . . . 1<br>NO 2	 → 930
927	How many months ago was your most recent HIV test?	MONTHS AGO . . . . [ ][ ]<br>TWO OR MORE YEARS 95	
928	I don't want to know the results, but did you get the results of the test?	YES . . . . 1<br>NO 2	
929	Where was the test done? **(4)**<br><br>PROBE TO IDENTIFY THE TYPE OF SOURCE.<br><br>IF UNABLE TO DETERMINE IF PUBLIC OR PRIVATE SECTOR, WRITE THE NAME OF THE PLACE.<br><br>____________<br>(NAME OF PLACE)	PUBLIC SECTOR<br>GOVERNMENT HOSPITAL . . . . 11<br>GOVT. HEALTH CENTER . . . . 12<br>STAND-ALONE VCT CENTER . . . . . 13<br>FAMILY PLANNING CLINIC . . . . 14<br>MOBILE CLINIC . . . . 15<br>FIELDWORKER . . . . 16<br>SCHOOL BASED CLINIC . . . . 17<br>OTHER PUBLIC SECTOR ________ 18<br>(SPECIFY)<br>PRIVATE MEDICAL SECTOR<br>PRIVATE HOSPITAL/CLINIC/ PRIVATE DOCTOR . . . . 21<br>STAND-ALONE VCT CENTER . . . . . 22<br>PHARMACY . . . . 23<br>MOBILE CLINIC . . . . 24<br>FIELDWORKER . . . . 25<br>SCHOOL BASED CLINIC . . . . 26<br>OTHER PRIVATE MEDICAL SECTOR<br>________ 27<br>(SPECIFY)<br>OTHER SOURCE<br>HOME . . . . 31<br>CORRECTIONAL FACILITY . . . . 32<br><br>OTHER ________ 96<br>(SPECIFY)	→ 932
930	Do you know of a place where people can go to get tested for the AIDS virus?	YES . . . . 1<br>NO 2	 → 932

NO.	QUESTIONS AND FILTERS	CODING CATEGORIES	SKIP
931	Where is that? **(4)**<br><br>Any other place?<br><br>PROBE TO IDENTIFY EACH TYPE OF SOURCE.<br><br>IF UNABLE TO DETERMINE IF PUBLIC OR PRIVATE SECTOR, WRITE THE NAME OF THE PLACE.<br><br>____________________<br>(NAME OF PLACE(S))	PUBLIC SECTOR<br>GOVERNMENT HOSPITAL . . . . A<br>GOVT. HEALTH CENTER . . . . B<br>STAND-ALONE VCT CENTER . . . . . C<br>FAMILY PLANNING CLINIC . . . . D<br>MOBILE CLINIC . . . . E<br>FIELDWORKER . . . . F<br>OTHER PUBLIC SECTOR ________ G<br>(SPECIFY)<br><br>PRIVATE MEDICAL SECTOR<br>PRIVATE HOSPITAL/CLINIC/ PRIVATE DOCTOR . . . . H<br>STAND-ALONE VCT CENTER . . . . . I<br>PHARMACY . . . . J<br>MOBILE CLINIC . . . . K<br>FIELDWORKER . . . . L<br>OTHER PRIVATE MEDICAL SECTOR<br>________ M<br>(SPECIFY)<br><br>OTHER ________ X<br>(SPECIFY)	
932	Would you buy fresh vegetables from a shopkeeper or vendor if you knew that this person had the AIDS virus?	YES . . . . 1<br>NO . . . . 2<br>DON'T KNOW 8	
933	If a member of your family got infected with the AIDS virus, would you want it to remain a secret or not?	YES, REMAIN A SECRET . . . . 1<br>NO . . . . 2<br>DK/NOT SURE/DEPENDS 8	
934	If a member of your family became sick with AIDS, would you be willing to care for her or him in your own household?	YES . . . . 1<br>NO . . . . 2<br>DK/NOT SURE/DEPENDS 8	
935	In your opinion, if a female teacher has the AIDS virus but is not sick, should she be allowed to continue teaching in the school?	SHOULD BE ALLOWED . . . . 1<br>SHOULD NOT BE ALLOWED . . . . 2<br>DK/NOT SURE/DEPENDS 8	
936 **(2)**	Should children age 12-14 be taught about using a condom to avoid getting AIDS?	YES . . . . 1<br>NO . . . . 2<br>DK/NOT SURE/DEPENDS 8	
937	CHECK 901:<br>HEARD ABOUT AIDS ☐ → Apart from AIDS, have you heard about other infections that can be transmitted through sexual contact?<br>NOT HEARD ABOUT AIDS ☐ → Have you heard about infections that can be transmitted through sexual contact?	YES . . . . 1<br>NO 2	
938	CHECK 613:<br>HAS HAD SEXUAL INTERCOURSE ☐ ↓<br>NEVER HAD SEXUAL INTERCOURSE ☐ →		946

NO.	QUESTIONS AND FILTERS	CODING CATEGORIES	SKIP
939	CHECK 937: HEARD ABOUT OTHER SEXUALLY TRANSMITTED INFECTIONS?<br>YES ☐ (↓) NO ☐		→ 941
940	Now I would like to ask you some questions about your health in the last 12 months. During the last 12 months, have you had a disease which you got through sexual contact?	YES . . . . . . 1<br>NO . . . . . . 2<br>DON'T KNOW 8	
941	Sometimes women experience a bad-smelling abnormal genital discharge.<br>During the last 12 months, have you had a bad-smelling abnormal genital discharge?	YES . . . . . . 1<br>NO . . . . . . 2<br>DON'T KNOW 8	
942	Sometimes women have a genital sore or ulcer. During the last 12 months, have you had a genital sore or ulcer?	YES . . . . . . 1<br>NO . . . . . . 2<br>DON'T KNOW 8	
943	CHECK 940, 941, AND 942:<br>HAS HAD AN INFECTION (ANY 'YES') ☐ (↓)<br>HAS NOT HAD AN INFECTION OR DOES NOT KNOW ☐		→ 946
944	The last time you had (PROBLEM FROM 940/941/942), did you seek any kind of advice or treatment?	YES . . . . . . 1<br>NO 2	 → 946
945	Where did you go? **(4)**<br><br>Any other place?<br><br>PROBE TO IDENTIFY EACH TYPE OF SOURCE.<br><br>IF UNABLE TO DETERMINE IF PUBLIC OR PRIVATE SECTOR, WRITE THE NAME OF THE PLACE.<br><br>________________<br>(NAME OF PLACE(S))	PUBLIC SECTOR<br>GOVERNMENT HOSPITAL . . . . A<br>GOVT. HEALTH CENTER . . . . B<br>STAND-ALONE VCT CENTER . . . C<br>FAMILY PLANNING CLINIC . . . . D<br>MOBILE CLINIC . . . . E<br>FIELDWORKER . . . . F<br>OTHER PUBLIC SECTOR ________ G<br>(SPECIFY)<br><br>PRIVATE MEDICAL SECTOR<br>PRIVATE HOSPITAL/CLINIC/ PRIVATE DOCTOR . . . . H<br>STAND-ALONE VCT CENTER . . . I<br>PHARMACY . . . . J<br>MOBILE CLINIC . . . . K<br>FIELDWORKER . . . . L<br>OTHER PRIVATE MEDICAL SECTOR ________ M<br>(SPECIFY)<br><br>OTHER SOURCE<br>SHOP . . . . N<br>OTHER ________ X<br>(SPECIFY)	
946 **(2)**	If a wife knows her husband has a disease that she can get during sexual intercourse, is she justified in asking that they use a condom when they have sex?	YES . . . . . . 1<br>NO . . . . . . 2<br>DON'T KNOW 8	
947	Is a wife justified in refusing to have sex with her husband when she knows he has sex with other women? **(5)**	YES . . . . . . 1<br>NO . . . . . . 2<br>DON'T KNOW 8	

NO.	QUESTIONS AND FILTERS	CODING CATEGORIES	SKIP
948 (2)	CHECK 601: CURRENTLY MARRIED/ LIVING WITH A MAN ☐ NOT IN UNION ☐		1001
949 (2)	Can you say no to your (husband/partner) if you do not want to have sexual intercourse?	YES . . . . . . 1<br>NO . . . . . . 2<br>DEPENDS/NOT SURE 8	
950 (2)	Could you ask your (husband/partner) to use a condom if you wanted him to?	YES . . . . . . 1<br>NO . . . . . . 2<br>DEPENDS/NOT SURE 8	

(1) If Qs. 903, 905 and/or 906 do not apply to the local context, replace the question using a specific local misconception. At least two questions related to misconceptions are needed.
(2) The question may be considered for deletion in countries with a very low HIV prevalence.
(3) Year of fieldwork is assumed to be 2010. For fieldwork in 2011 or 2012, the year should be 2009 and 2010, respectively.
(4) Coding categories to be developed locally and revised based on the pretest; however, the broad categories must be maintained.
(5) In polygynous societies, the phrase 'other women' should be replaced by the phrase 'women other than his wives.'

SECTION 10. OTHER HEALTH ISSUES

NO.	QUESTIONS AND FILTERS	CODING CATEGORIES	SKIP
1001	Now I would like to ask you some other questions relating to health matters. Have you had an injection for any reason in the last 12 months?<br><br>IF YES: How many injections have you had?<br><br>IF NUMBER OF INJECTIONS IS 90 OR MORE,<br>OR DAILY FOR 3 MONTHS OR MORE, RECORD '90'.<br><br>IF NON-NUMERIC ANSWER, PROBE TO GET AN ESTIMATE.	NUMBER OF INJECTIONS . . . [ ][ ]<br><br>NONE 00	 <br>→ 1004
1002	Among these injections, how many were administered by a doctor, a nurse, a pharmacist, a dentist, or any other health worker?<br><br>IF NUMBER OF INJECTIONS IS 90 OR MORE,<br>OR DAILY FOR 3 MONTHS OR MORE, RECORD '90'.<br><br>IF NON-NUMERIC ANSWER, PROBE TO GET AN ESTIMATE.	NUMBER OF INJECTIONS . . . [ ][ ]<br><br>NONE 00	 <br>→ 1004
1003	The last time you got an injection from a health worker, did he/she take the syringe and needle from a new, unopened package?	YES . . . . 1<br>NO . . . . 2<br>DON'T KNOW 8	
1004	Do you currently smoke cigarettes?	YES . . . . 1<br>NO 2	 → 1006
1005	In the last 24 hours, how many cigarettes did you smoke?	NUMBER OF<br>CIGARETTES . . . . [ ][ ]	
1006	Do you currently smoke or use any (other) type of tobacco? **(1)**	YES . . . . 1<br>NO 2	 → 1008
1007	What (other) type of tobacco do you currently smoke or use? **(1)**<br><br>RECORD ALL MENTIONED.	PIPE . . . . A<br>CHEWING TOBACCO . . . . B<br>SNUFF . . . . C<br><br>OTHER ______________________ X<br>(SPECIFY)	
1008	Many different factors can prevent women from getting medical advice or treatment for themselves. When you are sick and want to get medical advice or treatment, is each of the following a big problem or not?	BIG PROBLEM / NOT A BIG PROBLEM	
	Getting permission to go to the doctor?	PERMISSION TO GO . . . 1 2	
	Getting money needed for advice or treatment?	GETTING MONEY 1 2	
	The distance to the health facility?	DISTANCE 1 2	
	Not wanting to go alone?	GO ALONE 1 2	
1009	Are you covered by any health insurance? **(2)**	YES . . . . 1<br>NO 2	 → 1011

NO.	QUESTIONS AND FILTERS	CODING CATEGORIES	SKIP
1010	What type of health insurance are you covered by? **(2)**<br><br>RECORD ALL MENTIONED.	MUTUAL HEALTH ORGANIZATION/<br>COMMUNITY-BASED HEALTH<br>INSURANCE . . . . A<br>HEALTH INSURANCE THROUGH<br>EMPLOYER . . . . B<br>SOCIAL SECURITY . . . . C<br>OTHER PRIVATELY PURCHASED<br>COMMERCIAL HEALTH INSURANCE D<br>OTHER ______________________ X<br>(SPECIFY)	
1011	RECORD THE TIME.	HOUR . . . . [ ][ ]<br>MINUTES . . . . [ ][ ]	

(1) Add local terms.
(2) If a health service prepayment plan or other types of plans are available in the country, add those types of plans to the question.

INTERVIEWER'S OBSERVATIONS

TO BE FILLED IN AFTER COMPLETING INTERVIEW

COMMENTS ABOUT RESPONDENT:

COMMENTS ON SPECIFIC QUESTIONS:

ANY OTHER COMMENTS:

SUPERVISOR'S OBSERVATIONS

NAME OF SUPERVISOR: ____________________ DATE: ____________________

EDITOR'S OBSERVATIONS

NAME OF EDITOR: ____________________ DATE: ____________________

INSTRUCTIONS:
ONLY ONE CODE SHOULD APPEAR IN ANY BOX.
COLUMN 1 REQUIRES A CODE IN EVERY MONTH.

INFORMATION TO BE CODED FOR EACH COLUMN

COLUMN 1: BIRTHS, PREGNANCIES, CONTRACEPTIVE USE**

B BIRTHS
P PREGNANCIES
T TERMINATIONS

0 NO METHOD
1 FEMALE STERILIZATION
2 MALE STERILIZATION
3 IUD
4 INJECTABLES
5 IMPLANTS
6 PILL
7 CONDOM
8 FEMALE CONDOM
9 DIAPHRAGM
J FOAM OR JELLY
K LACTATIONAL AMENORRHEA METHOD
L RHYTHM METHOD
M WITHDRAWAL
X OTHER MODERN METHOD
Y OTHER TRADITIONAL METHOD

COLUMN 2: DISCONTINUATION OF CONTRACEPTIVE USE

0 INFREQUENT SEX/HUSBAND AWAY
1 BECAME PREGNANT WHILE USING
2 WANTED TO BECOME PREGNANT
3 HUSBAND/PARTNER DISAPPROVED
4 WANTED MORE EFFECTIVE METHOD
5 SIDE EFFECTS/HEALTH CONCERNS
6 LACK OF ACCESS/TOO FAR
7 COSTS TOO MUCH
8 INCONVENIENT TO USE
F UP TO GOD/FATALISTIC
A DIFFICULT TO GET PREGNANT/MENOPAUSAL
D MARITAL DISSOLUTION/SEPARATION
X OTHER ______________________
(SPECIFY)
Z DON'T KNOW

				1	2	
	12	DEC	01			
	11	NOV	02			
	10	OCT	03			
	09	SEP	04			
2	08	AUG	05			2
0	07	JUL	06			0
1	06	JUN	07			1
0	05	MAY	08			0
*	04	APR	09			*
	03	MAR	10			
	02	FEB	11			
	01	JAN	12			
	12	DEC	13			
	11	NOV	14			
	10	OCT	15			
	09	SEP	16			
2	08	AUG	17			2
0	07	JUL	18			0
0	06	JUN	19			0
9	05	MAY	20			9
*	04	APR	21			*
	03	MAR	22			
	02	FEB	23			
	01	JAN	24			
	12	DEC	25			
	11	NOV	26			
	10	OCT	27			
	09	SEP	28			
2	08	AUG	29			2
0	07	JUL	30			0
0	06	JUN	31			0
8	05	MAY	32			8
*	04	APR	33			*
	03	MAR	34			
	02	FEB	35			
	01	JAN	36			
	12	DEC	37			
	11	NOV	38			
	10	OCT	39			
	09	SEP	40			
2	08	AUG	41			2
0	07	JUL	42			0
0	06	JUN	43			0
7	05	MAY	44			7
*	04	APR	45			*
	03	MAR	46			
	02	FEB	47			
	01	JAN	48			
	12	DEC	49			
	11	NOV	50			
	10	OCT	51			
	09	SEP	52			
2	08	AUG	53			2
0	07	JUL	54			0
0	06	JUN	55			0
6	05	MAY	56			6
*	04	APR	57			*
	03	MAR	58			
	02	FEB	59			
	01	JAN	60			
	12	DEC	61			
	11	NOV	62			
	10	OCT	63			
	09	SEP	64			
2	08	AUG	65			2
0	07	JUL	66			0
0	06	JUN	67			0
5	05	MAY	68			5
*	04	APR	69			*
	03	MAR	70			
	02	FEB	71			
	01	JAN	72			

* Year of fieldwork is assumed to be 2010. For fieldwork beginning in 2011 or 2012, the years should be adjusted.

** Response categories may be added for other methods, including fertility awareness methods.

[THIS PAGE IS INTENTIONALLY BLANK]

DEMOGRAPHIC AND HEALTH SURVEYS
MODEL MAN'S QUESTIONNAIRE

[NAME OF COUNTRY]
[NAME OF ORGANIZATION]

**IDENTIFICATION (1)**	
PLACE NAME ______________________	
NAME OF HOUSEHOLD HEAD ______________________	
CLUSTER NUMBER	☐☐☐
HOUSEHOLD NUMBER	☐☐
NAME AND LINE NUMBER OF MAN ______________________	☐☐

INTERVIEWER VISITS

	1	2	3	FINAL VISIT
DATE	________	________	________	DAY ☐☐<br>MONTH ☐☐<br>YEAR ☐☐☐☐
INTERVIEWER'S NAME	________	________	________	INT. NUMBER ☐☐☐
RESULT*	________	________	________	RESULT ☐
NEXT VISIT: DATE<br>TIME	________<br>________	________<br>________		TOTAL NUMBER OF VISITS ☐

*RESULT CODES:
1 COMPLETED
2 NOT AT HOME
3 POSTPONED
4 REFUSED
5 PARTLY COMPLETED
6 INCAPACITATED
7 OTHER ______________________ (SPECIFY)

COUNTRY-SPECIFIC INFORMATION: LANGUAGE OF QUESTIONNAIRE, LANGUAGE OF INTERVIEW, NATIVE LANGUAGE OF RESPONDENT, AND WHETHER TRANSLATOR USED

SUPERVISOR	FIELD EDITOR	OFFICE EDITOR	KEYED BY
NAME ________ ☐☐☐	NAME ________ ☐☐☐	☐☐	☐☐

(1) This section should be adapted for country-specific survey design.

Note: Questions with blue highlighting in the question number column are HIV related questions that may be deleted in some circumstances (see footnotes). Questions with yellow highlighting in the question number column are other questions that may be deleted in some circumstances (see footnotes).

SECTION 1. RESPONDENT'S BACKGROUND

INTRODUCTION AND CONSENT

INFORMED CONSENT

Hello. My name is ______________________________________. I am working with (NAME OF ORGANIZATION). We are conducting a survey about health all over (NAME OF COUNTRY). The information we collect will help the government to plan health services. Your household was selected for the survey. The questions usually take about 20 minutes. All of the answers you give will be confidential and will not be shared with anyone other than members of our survey team. You don't have to be in the survey, but we hope you will agree to answer the questions since your views are important. If I ask you any question you don't want to answer, just let me know and I will go on to the next question or you can stop the interview at any time.

In case you need more information about the survey, you may contact the person listed on the card that has already been given to your household.
Do you have any questions? May I begin the interview now?

SIGNATURE OF INTERVIEWER:______________________________ DATE: ____________________

RESPONDENT AGREES TO BE INTERVIEWED . . . . . 1 ↓ RESPONDENT DOES NOT AGREE TO BE INTERVIEWED . . . 2 → END

NO.	QUESTIONS AND FILTERS	CODING CATEGORIES	SKIP
101	RECORD THE TIME.	HOUR . . . . [ ][ ]<br>MINUTES . . . . [ ][ ]	
102	In what month and year were you born?	MONTH . . . . [ ][ ]<br>DON'T KNOW MONTH . . . . 98<br>YEAR . . . . [ ][ ][ ][ ]<br>DON'T KNOW YEAR 9998	
103	How old were you at your last birthday?<br><br>COMPARE AND CORRECT 102 AND/OR 103 IF INCONSISTENT.	AGE IN COMPLETED YEARS [ ][ ]	
104	Have you ever attended school?	YES . . . . 1<br>NO 2	 → 108
105	What is the highest level of school you attended: primary, secondary, or higher? **(1)**	PRIMARY . . . . 1<br>SECONDARY . . . . 2<br>HIGHER 3	
106	What is the highest (grade/form/year) you completed at that level? **(1)**<br><br>IF COMPLETED LESS THAN ONE YEAR AT THAT LEVEL, RECORD '00'.	GRADE/FORM/YEAR . . . . [ ][ ]	

NO.	QUESTIONS AND FILTERS	CODING CATEGORIES	SKIP
107	CHECK 105:<br>PRIMARY ☐ ↓ SECONDARY OR HIGHER ☐		→ 110
108	Now I would like you to read this sentence to me.<br><br>SHOW CARD TO RESPONDENT. **(2)**<br><br>IF RESPONDENT CANNOT READ WHOLE SENTENCE, PROBE:<br>Can you read any part of the sentence to me?	CANNOT READ AT ALL . . . . 1<br>ABLE TO READ ONLY PARTS OF SENTENCE . . . . 2<br>ABLE TO READ WHOLE SENTENCE 3<br>NO CARD WITH REQUIRED LANGUAGE ______ 4<br>(SPECIFY LANGUAGE)<br>BLIND/VISUALLY IMPAIRED 5	
109	CHECK 108:<br>CODE '2', '3' OR '4' CIRCLED ☐ ↓ CODE '1' OR '5' CIRCLED ☐		→ 111
110	Do you read a newspaper or magazine, at least once a week, less than once a week or not at all?	AT LEAST ONCE A WEEK . . . . 1<br>LESS THAN ONCE A WEEK . . . . 2<br>NOT AT ALL 3	
111	Do you listen to the radio, at least once a week, less than once a week or not at all?	AT LEAST ONCE A WEEK . . . . 1<br>LESS THAN ONCE A WEEK . . . . 2<br>NOT AT ALL 3	
112	Do you watch television, at least once a week, less than once a week or not at all?	AT LEAST ONCE A WEEK . . . . 1<br>LESS THAN ONCE A WEEK . . . . 2<br>NOT AT ALL 3	
113	COUNTRY-SPECIFIC QUESTION ON RELIGION, IF APPROPRIATE.		
114	COUNTRY-SPECIFIC QUESTION ON ETHNICITY, IF APPROPRIATE.		
115 **(3)**	In the last 12 months, how many times have you been away from home for one or more nights?	NUMBER OF TIMES . . . . ☐☐<br>NONE 00	 → 201
116 **(3)**	In the last 12 months, have you been away from home for more than one month at a time?	YES . . . . 1<br>NO 2	

(1) Revise according to the local education system.
(2) Each card should have four simple sentences appropriate to the country (e.g., "Parents love their children.", "Farming is hard work.", "The child is reading a book.", "Children work hard at school."). Cards should be prepared for every language in which respondents are likely to be literate.
(3) The question may be considered for deletion in countries with a very low HIV prevalence.

SECTION 2. REPRODUCTION

NO.	QUESTIONS AND FILTERS	CODING CATEGORIES	SKIP
201	Now I would like to ask about any children you have had during your life. I am interested in all of the children that are biologically yours, even if they are not legally yours or do not have your last name.<br><br>Have you ever fathered any children with any woman?	YES . . . . . 1<br>NO . . . . . 2<br>DON'T KNOW 8	 2, 8 → 206
202	Do you have any sons or daughters that you have fathered who are now living with you?	YES . . . . . 1<br>NO 2	 → 204
203	How many sons live with you?<br><br>And how many daughters live with you?<br><br>IF NONE, RECORD '00'.	SONS AT HOME . . . . . [ ][ ]<br>DAUGHTERS AT HOME . . . . . [ ][ ]	
204	Do you have any sons or daughters that you have fathered who are alive but do not live with you?	YES . . . . . 1<br>NO 2	 → 206
205	How many sons are alive but do not live with you?<br><br>And how many daughters are alive but do not live with you?<br><br>IF NONE, RECORD '00'.	SONS ELSEWHERE . . . . . [ ][ ]<br>DAUGHTERS ELSEWHERE . . . [ ][ ]	
206	Have you ever fathered a son or a daughter who was born alive but later died?<br><br>IF NO, PROBE: Any baby who cried or showed signs of life but did not survive?	YES . . . . . 1<br>NO . . . . . 2<br>DON'T KNOW 8	 2, 8 → 208
207	How many boys have died?<br><br>And how many girls have died?<br><br>IF NONE, RECORD '00'.	BOYS DEAD . . . . . [ ][ ]<br>GIRLS DEAD . . . . . [ ][ ]	
208	SUM ANSWERS TO 203, 205, AND 207, AND ENTER TOTAL.<br><br>IF NONE, RECORD '00'.	TOTAL CHILDREN . . . . . [ ][ ]	
209	CHECK 208:<br>HAS HAD MORE THAN ONE CHILD [ ] ↓<br>HAS HAD ONLY ONE CHILD [ ] → 212<br>HAS NOT HAD ANY CHILDREN [ ] → 301		212<br>301
210	Did all of the children you have fathered have the same biological mother?	YES . . . . . 1<br>NO 2	→ 212
211	In all, how many women have you fathered children with?	NUMBER OF WOMEN . . . . . [ ][ ]	
212	How old were you when your (first) child was born?	AGE IN YEARS . . . . . [ ][ ]	
213	CHECK 203 AND 205:<br>AT LEAST ONE LIVING CHILD [ ] ↓<br>NO LIVING CHILDREN [ ] → 301		301
214	How old is your (youngest) child?	AGE IN YEARS . . . . . [ ][ ]	

NO.	QUESTIONS AND FILTERS	CODING CATEGORIES	SKIP
215	CHECK 214:<br>(YOUNGEST) CHILD IS AGE 0-2 YEARS ☐ (continue)<br>OTHER ☐		→ 301
216	What is the name of your (youngest) child?<br>WRITE NAME OF (YOUNGEST) CHILD<br>____________________<br>(NAME OF (YOUNGEST) CHILD)		
217	When (NAME)'s mother was pregnant with (NAME), did she have any antenatal check-ups?	YES . . . . 1<br>NO . . . . 2<br>DON'T KNOW 8	 2, 8 → 219
218	Were you ever present during any of those antenatal check-ups?	PRESENT . . . . 1<br>NOT PRESENT 2	
219	Was (NAME) born in a hospital or health facility?	HOSPITAL/HEALTH FACILITY . . . . . 1<br>OTHER 2	
220	When a child has diarrhea, how much should he or she be given to drink: more than usual, about the same as usual, less than usual, or nothing to drink at all?	MORE THAN USUAL . . . . 1<br>ABOUT THE SAME . . . . 2<br>LESS THAN USUAL . . . . 3<br>NOTHING TO DRINK . . . . 4<br>DON'T KNOW 8	

301	Now I would like to talk about family planning - the various ways or methods that a couple can use to delay or avoid a pregnancy.<br><br>Have you ever heard of (METHOD)? **(1)**		
01	**Female Sterilization**. PROBE: Women can have an operation to avoid having any more children.	YES . . . . 1<br>NO 2	
02	**Male Sterilization**. PROBE: Men can have an operation to avoid having any more children.	YES . . . . 1<br>NO 2	
03	**IUD**. PROBE: Women can have a loop or coil placed inside them by a doctor or a nurse.	YES . . . . 1<br>NO 2	
04	**Injectables**. PROBE: Women can have an injection by a health provider that stops them from becoming pregnant for one or more months.	YES . . . . 1<br>NO 2	
05	**Implants**. PROBE: Women can have one or more small rods placed in their upper arm by a doctor or nurse which can prevent pregnancy for one or more years.	YES . . . . 1<br>NO 2	
06	**Pill**. PROBE: Women can take a pill every day to avoid becoming pregnant.	YES . . . . 1<br>NO 2	
07	**Condom**. PROBE: Men can put a rubber sheath on their penis before sexual intercourse.	YES . . . . 1<br>NO 2	
08	**Female Condom**. PROBE: Women can place a sheath in their vagina before sexual intercourse.	YES . . . . 1<br>NO 2	
09<br>**(2)**	**Lactational Amenorrhea Method (LAM)**. **(2)**	YES . . . . 1<br>NO 2	
10	**Rhythm Method**. PROBE: To avoid pregnancy, women do not have sexual intercourse on the days of the month they think they can get pregnant.	YES . . . . 1<br>NO 2	
11	**Withdrawal**. PROBE: Men can be careful and pull out before climax.	YES . . . . 1<br>NO 2	
12	**Emergency Contraception**. PROBE: As an emergency measure, within three days after they have unprotected sexual intercourse, women can take special pills to prevent pregnancy. **(3)**	YES . . . . 1<br>NO 2	
13	Have you heard of any other ways or methods that women or men can use to avoid pregnancy?	YES . . . . 1<br>__________________<br>(SPECIFY)<br>__________________<br>(SPECIFY)<br>NO 2	

NO.	QUESTIONS AND FILTERS	CODING CATEGORIES	SKIP
302	In the last few months have you:<br>Heard about family planning on the radio?<br>Seen anything about family planning on the television?<br>Read about family planning in a newspaper or magazine?	YES NO<br>RADIO . . . . 1 2<br>TELEVISION . . . . 1 2<br>NEWSPAPER OR MAGAZINE 1 2	
303	In the last few months, have you discussed family planning with a health worker or health professional?	YES . . . . 1<br>NO 2	
304	Now I would like to ask you about a woman's risk of pregnancy.<br>From one menstrual period to the next, are there certain days when a woman is more likely to become pregnant when she has sexual relations?	YES . . . . 1<br>NO . . . . 2<br>DON'T KNOW 8	NO, DON'T KNOW → 306
305	Is this time just before her period begins, during her period, right after her period has ended, or halfway between two periods?	JUST BEFORE HER<br>PERIOD BEGINS . . . . 1<br>DURING HER PERIOD . . . . 2<br>RIGHT AFTER HER<br>PERIOD HAS ENDED . . . . 3<br>HALFWAY BETWEEN<br>TWO PERIODS . . . . 4<br>OTHER ______________ 6<br>(SPECIFY)<br>DON'T KNOW 8	
306	I will now read you some statements about contraception. Please tell me if you agree or disagree with each one.<br>a) Contraception is a woman's business and a man should not have to worry about it.<br>b) Women who use contraception may become promiscuous.	AGREE DIS-AGREE DK<br>CONTRACEPTION<br>WOMAN'S BUSINESS 1 2 8<br>WOMEN MAY BECOME<br>PROMISCUOUS 1 2 8	
307	CHECK 301 (07): KNOWS MALE CONDOM<br>YES ☐ ↓ NO ☐ →		NO → 311
308	Do you know of a place where a person can get condoms?	YES . . . . 1<br>NO 2	NO → 311
309	Where is that? **(4)**<br>Any other place?<br>PROBE TO IDENTIFY EACH TYPE OF SOURCE.<br>IF UNABLE TO DETERMINE IF PUBLIC OR PRIVATE SECTOR, WRITE THE NAME OF THE PLACE.<br>______________________________<br>(NAME OF PLACE(S))	PUBLIC SECTOR<br>GOVERNMENT HOSPITAL . . . . A<br>GOVT. HEALTH CENTER . . . . B<br>FAMILY PLANNING CLINIC . . . . C<br>MOBILE CLINIC . . . . D<br>FIELDWORKER . . . . E<br>OTHER PUBLIC<br>SECTOR ______________ F<br>(SPECIFY)<br>PRIVATE MEDICAL SECTOR<br>PRIVATE HOSPITAL/CLINIC . . . . . G<br>PHARMACY . . . . H<br>PRIVATE DOCTOR . . . . I<br>MOBILE CLINIC . . . . J<br>FIELDWORKER . . . . K<br>OTHER PRIVATE MEDICAL<br>SECTOR ______________ L<br>(SPECIFY)<br>OTHER SOURCE<br>SHOP . . . . M<br>CHURCH . . . . N<br>FRIENDS/RELATIVES . . . . O<br>OTHER ______________ X<br>(SPECIFY)	

NO.	QUESTIONS AND FILTERS	CODING CATEGORIES	SKIP
310	If you wanted to, could you yourself get a condom?	YES . . . . . 1<br>NO 2	
311<br>**(5)**	CHECK 301 (08): KNOWS FEMALE CONDOM<br>YES ☐ NO ☐		NO → 401
312<br>**(5)**	Do you know of a place where a person can get female condoms?	YES . . . . . 1<br>NO 2	 → 401
313<br>**(5)**	Where is that? **(4)**<br><br>Any other place?<br><br>PROBE TO IDENTIFY EACH TYPE OF SOURCE.<br><br>IF UNABLE TO DETERMINE IF PUBLIC OR PRIVATE SECTOR, WRITE THE NAME OF THE PLACE.<br><br>________________<br>(NAME OF PLACE(S))	PUBLIC SECTOR<br>GOVERNMENT HOSPITAL . . . . . A<br>GOVT. HEALTH CENTER . . . . . B<br>FAMILY PLANNING CLINIC . . . . . C<br>MOBILE CLINIC . . . . . D<br>FIELDWORKER . . . . . E<br>OTHER PUBLIC SECTOR ________ F<br>(SPECIFY)<br><br>PRIVATE MEDICAL SECTOR<br>PRIVATE HOSPITAL/CLINIC . . . . . G<br>PHARMACY . . . . . H<br>PRIVATE DOCTOR . . . . . I<br>MOBILE CLINIC . . . . . J<br>FIELDWORKER . . . . . K<br>OTHER PRIVATE MEDICAL SECTOR ________ L<br>(SPECIFY)<br><br>OTHER SOURCE<br>SHOP . . . . . M<br>CHURCH . . . . . N<br>FRIEND/RELATIVE . . . . . O<br><br>OTHER ________ X<br>(SPECIFY)	
314<br>**(5)**	If you wanted to, could you yourself get a female condom?	YES . . . . . 1<br>NO 2	

(1) If Standard Days Method is commonly used, it may be added to the table before Lactational Amenorrhea. "**Standard Days Method** (use local term, such as CycleBeads™ , as appropriate) PROBE: A woman uses a string of colored beads to know the days she can get pregnant. On the days she can get pregnant, she uses a condom or does not have sexual intercourse."

(2) The LAM method should be deleted in countries that do not have a LAM program. In these countries, LAM should also be deleted as a coding category in Q. 439. A description of LAM should not be provided in Q. 301.

(3) Studies have indicated emergency contraception can be effective up to five days. Verify country program recommendations and modify wording if appropriate.

(4) Coding categories to be developed locally and revised based on the pretest; however, the broad categories must be maintained.

(5) The question should be deleted in countries where female condoms are not actively promoted.

NO.	QUESTIONS AND FILTERS	CODING CATEGORIES	SKIP
401	Are you currently married or living together with a woman as if married?	YES, CURRENTLY MARRIED . . . . 1<br>YES, LIVING WITH A WOMAN . . . . . 2<br>NO, NOT IN UNION 3	1, 2 → 404
402	Have you ever been married or lived together with a woman as if married?	YES, FORMERLY MARRIED . . . . 1<br>YES, LIVED WITH A WOMAN . . . . 2<br>NO 3	3 → 413
403	What is your marital status now: are you widowed, divorced, or separated?	WIDOWED . . . . 1<br>DIVORCED . . . . 2<br>SEPARATED 3	1, 2, 3 → 410
404	Is your (wife/partner) living with you now or is she staying elsewhere?	LIVING WITH HIM . . . . 1<br>STAYING ELSEWHERE 2	
405<br>**(1)**	Do you have other wives or do you live with other women as if married?	YES (MORE THAN ONE) . . . . 1<br>NO (ONLY ONE) 2	2 → 407
406<br>**(1)**	Altogether, how many wives or live-in partners do you have?	TOTAL NUMBER OF WIVES AND LIVE-IN PARTNERS . . . __ __	
407<br>**(1)**	CHECK 405:<br>ONE WIFE/ PARTNER ☐ ↓ Please tell me the name of (your wife/the woman you are living with as if married).<br>MORE THAN ONE WIFE/ PARTNER ☐ ↓ Please tell me the name of each of your wives or each woman you are living with as if married.<br>RECORD THE NAME AND THE LINE NUMBER FROM THE HOUSEHOLD QUESTIONNAIRE FOR EACH WIFE AND LIVE-IN PARTNER.<br>IF A WOMAN IS NOT LISTED IN THE HOUSEHOLD, RECORD '00'.	NAME / LINE NUMBER / 408 How old was (NAME) on her last birthday? **(1)** AGE<br>______ / __ __ / __ __<br>______ / __ __ / __ __<br>______ / __ __ / __ __<br>______ / __ __ / __ __	
408<br>**(1)**	ASK 408 FOR EACH PERSON.		
409<br>**(1)**	CHECK 407:<br>ONE WIFE/ PARTNER ☐ ↓<br>MORE THAN ONE WIFE/ PARTNER ☐		→ 411A
410	Have you been married or lived with a woman only once or more than once?	ONLY ONCE . . . . 1<br>MORE THAN ONCE 2	2 → 411A

NO.	QUESTIONS AND FILTERS	CODING CATEGORIES	SKIP
411<br><br>411A	In what month and year did you start living with your (wife/partner)?<br><br>Now I would like to ask about your first (wife/partner). In what month and year did you start living with her?	MONTH . . . . [ ][ ]<br>DON'T KNOW MONTH . . . . 98<br>YEAR . . . . [ ][ ][ ][ ]<br>DON'T KNOW YEAR 9998	→ 413
412	How old were you when you first started living with her?	AGE . . . . [ ][ ]	
413	CHECK FOR THE PRESENCE OF OTHERS.<br><br>BEFORE CONTINUING, MAKE EVERY EFFORT TO ENSURE PRIVACY.		
414	Now I would like to ask some questions about sexual activity in order to gain a better understanding of some important life issues.<br><br>How old were you when you had sexual intercourse for the very first time?	NEVER HAD SEXUAL INTERCOURSE . . . . .00<br>AGE IN YEARS . . . . [ ][ ]<br>FIRST TIME WHEN STARTED LIVING WITH (FIRST) WIFE/PARTNER 95	→ 501
415	Now I would like to ask you some questions about your recent sexual activity. Let me assure you again that your answers are completely confidential and will not be told to anyone. If we should come to any question that you don't want to answer, just let me know and we will go to the next question.		
416	When was the <u>last</u> time you had sexual intercourse?<br><br>IF LESS THAN 12 MONTHS, ANSWER MUST BE RECORDED IN DAYS, WEEKS OR MONTHS.<br>IF 12 MONTHS (ONE YEAR) OR MORE, ANSWER MUST BE RECORDED IN YEARS.	DAYS AGO . . . . 1 [ ][ ]<br>WEEKS AGO . . . . 2 [ ][ ]<br>MONTHS AGO . . . . 3 [ ][ ]<br>YEARS AGO . . . . 4 [ ][ ]	→ 430

		LAST SEXUAL PARTNER	SECOND-TO-LAST SEXUAL PARTNER	THIRD-TO-LAST SEXUAL PARTNER
417	When was the last time you had sexual intercourse with this person?		DAYS AGO 1<br>WEEKS AGO 2<br>MONTHS AGO 3	DAYS AGO 1<br>WEEKS AGO 2<br>MONTHS AGO 3
418	The last time you had sexual intercourse (with this second/third person), was a condom used? **(2)**	YES . . . . 1<br>NO . . . . 2<br>(SKIP TO 420)	YES . . . . 1<br>NO . . . . 2<br>(SKIP TO 420)	YES . . . . 1<br>NO . . . . 2<br>(SKIP TO 420)
419	Was a condom used every time you had sexual intercourse with this person in the last 12 months?	YES . . . . 1<br>NO 2	YES . . . . 1<br>NO 2	YES . . . . 1<br>NO 2
420	What was your relationship to this person with whom you had sexual intercourse? **(3)**<br><br>IF GIRLFRIEND:<br>Were you living together as if married?<br><br>IF YES, CIRCLE '2'.<br>IF NO, CIRCLE '3'.	WIFE . . . . 1<br>LIVE-IN PARTNER . . . . 2<br>GIRLFRIEND NOT LIVING WITH RESPONDENT . . . . 3<br>CASUAL ACQUAINTANCE . . . 4<br>CLIENT/PROSTITUTE 5<br>OTHER _____________ 6<br>(SPECIFY)<br>(SKIP TO 423)	WIFE . . . . 1<br>LIVE-IN PARTNER . . . . 2<br>GIRLFRIEND NOT LIVING WITH RESPONDENT . . . . 3<br>CASUAL ACQUAINTANCE . . . 4<br>CLIENT/PROSTITUTE 5<br>OTHER _____________ 6<br>(SPECIFY)<br>(SKIP TO 423)	WIFE . . . . 1<br>LIVE-IN PARTNER . . . . 2<br>GIRLFRIEND NOT LIVING WITH RESPONDENT . . . . 3<br>CASUAL ACQUAINTANCE . . . 4<br>CLIENT/PROSTITUTE 5<br>OTHER _____________ 6<br>(SPECIFY)<br>(SKIP TO 423)
421	CHECK 410:	MARRIED ONLY ONCE ☐<br>MARRIED MORE THAN ONCE OR BLANK ☐ (SKIP TO 423)	MARRIED ONLY ONCE ☐<br>MARRIED MORE THAN ONCE OR BLANK ☐ (SKIP TO 423)	MARRIED ONLY ONCE ☐<br>MARRIED MORE THAN ONCE OR BLANK ☐ (SKIP TO 423)
422	CHECK 414:	FIRST TIME WHEN STARTED LIVING WITH FIRST WIFE (SKIP TO 424)<br>OTHER ☐	FIRST TIME WHEN STARTED LIVING WITH FIRST WIFE (SKIP TO 424)<br>OTHER ☐	FIRST TIME WHEN STARTED LIVING WITH FIRST WIFE (SKIP TO 424)<br>OTHER ☐
423	How long ago did you first have sexual intercourse with this (second/third) person?	DAYS AGO 1<br>WEEKS AGO 2<br>MONTHS AGO 3<br>YEARS AGO 4	DAYS AGO 1<br>WEEKS AGO 2<br>MONTHS AGO 3<br>YEARS AGO 4	DAYS AGO 1<br>WEEKS AGO 2<br>MONTHS AGO 3<br>YEARS AGO 4
424	How many times during the last 12 months did you have sexual intercourse with this person?<br><br>IF NON-NUMERIC ANSWER, PROBE TO GET AN ESTIMATE.<br>IF NUMBER OF TIMES IS 95 OR MORE, WRITE '95'.	NUMBER OF TIMES	NUMBER OF TIMES	NUMBER OF TIMES

		LAST SEXUAL PARTNER	SECOND-TO-LAST SEXUAL PARTNER	THIRD-TO-LAST SEXUAL PARTNER
425	How old is this person?	AGE OF PARTNER [ ][ ]<br>DON'T KNOW 98	AGE OF PARTNER [ ][ ]<br>DON'T KNOW 98	AGE OF PARTNER [ ][ ]<br>DON'T KNOW 98
426	Apart from (this person/these two people), have you had sexual intercourse with any other person in the last 12 months?	YES . . . . 1<br>(GO BACK TO 417 IN NEXT COLUMN)<br>NO . . . . 2<br>(SKIP TO 428)	YES . . . . 1<br>(GO BACK TO 417 IN NEXT COLUMN)<br>NO . . . . 2<br>(SKIP TO 428)	
427	In total, with how many different people have you had sexual intercourse in the last 12 months?<br>IF NON-NUMERIC ANSWER, PROBE TO GET AN ESTIMATE.<br>IF NUMBER OF PARTNERS IS 95 OR MORE, WRITE '95'.			NUMBER OF PARTNERS LAST 12 MONTHS . . . [ ][ ]<br>DON'T KNOW . . . 98

NO.	QUESTIONS AND FILTERS	CODING CATEGORIES	SKIP
428	CHECK 420 (ALL COLUMNS):<br>AT LEAST ONE PARTNER IS PROSTITUTE ☐ ↓<br>NO PARTNERS ARE PROSTITUTES ☐ → 430		430
429	CHECK 420 AND 418 (ALL COLUMNS):<br>CONDOM USED WITH EVERY PROSTITUTE ☐ → 433<br>OTHER ☐ → 434		433<br>434
430	In the last 12 months, did you pay anyone in exchange for having sexual intercourse?	YES . . . . . 1<br>NO 2	1 → 432
431	Have you ever paid anyone in exchange for having sexual intercourse?	YES . . . . . 1<br>NO 2	1, 2 → 434
432	The last time you paid someone in exchange for having sexual intercourse, was a condom used? **(2)**	YES . . . . . 1<br>NO 2	2 → 434
433	Was a condom used during sexual intercourse every time you paid someone in exchange for having sexual intercourse in the last 12 months?	YES . . . . . 1<br>NO . . . . . 2<br>DON'T KNOW 8	
434	In total, with how many different people have you had sexual intercourse in your lifetime?<br>IF NON-NUMERIC ANSWER, PROBE TO GET AN ESTIMATE.<br>IF NUMBER OF PARTNERS IS 95 OR MORE, WRITE '95'.	NUMBER OF PARTNERS IN LIFETIME . . . . . ☐☐<br>DON'T KNOW 98	
435<br>**(2)**	CHECK 418, MOST RECENT PARTNER (FIRST COLUMN):<br>NOT ASKED ☐ → 438<br>CONDOM USED ☐ ↓<br>NO CONDOM USED ☐ → 438		438<br>438
436<br>**(2)**	You told me that a condom was used the last time you had sex. What is the brand name of the condom used at that time?<br>IF BRAND NOT KNOWN, ASK TO SEE THE PACKAGE.	BRAND A . . . . . 01<br>BRAND B . . . . . 02<br>BRAND C . . . . . 03<br>OTHER ______ 96<br>(SPECIFY)<br>DON'T KNOW 98	

NO.	QUESTIONS AND FILTERS	CODING CATEGORIES	SKIP
437<br>**(2)**	From where did you obtain the condom the last time? **(4)**<br><br>PROBE TO IDENTIFY TYPE OF SOURCE.<br><br>IF UNABLE TO DETERMINE IF PUBLIC OR PRIVATE SECTOR, WRITE THE NAME OF THE PLACE.<br><br>__________<br>(NAME OF PLACE)	PUBLIC SECTOR<br>GOVERNMENT HOSPITAL . . . . 11<br>GOVT. HEALTH CENTER . . . . 12<br>FAMILY PLANNING CLINIC . . . . 13<br>MOBILE CLINIC . . . . 14<br>FIELDWORKER . . . . 15<br>OTHER PUBLIC SECTOR ________ 16<br>(SPECIFY)<br><br>PRIVATE MEDICAL SECTOR<br>PRIVATE HOSPITAL/CLINIC . . . . . 21<br>PHARMACY . . . . 22<br>PRIVATE DOCTOR . . . . 23<br>MOBILE CLINIC . . . . 24<br>FIELDWORKER . . . . 25<br>OTHER PRIVATE MEDICAL SECTOR ________ 26<br>(SPECIFY)<br><br>OTHER SOURCE<br>SHOP . . . . 31<br>CHURCH . . . . 32<br>FRIEND/RELATIVE . . . . 33<br><br>OTHER ________ 96<br>(SPECIFY)	
438	The last time you had sex did you or your partner use any method (other than a condom) to avoid or prevent a pregnancy?	YES . . . . 1<br>NO . . . . 2<br>DON'T KNOW 8	 2, 8 → 501
439<br>**(5)**	What method did you or your partner use?<br><br>PROBE:<br>Did you or your partner use any other method to prevent pregnancy?<br><br>RECORD ALL MENTIONED.	FEMALE STERILIZATION . . . . A<br>MALE STERILIZATION . . . . B<br>IUD . . . . C<br>INJECTABLES . . . . D<br>IMPLANTS . . . . E<br>PILL . . . . F<br>FEMALE CONDOM . . . . G<br>DIAPHRAGM . . . . H<br>FOAM/JELLY . . . . I<br>LAM . . . . J<br>RHYTHM METHOD . . . . K<br>WITHDRAWAL . . . . L<br>OTHER MODERN METHOD . . . . X<br>OTHER TRADITIONAL METHOD . . . . . Y	

(1) Delete Qs. 405-409 in countries where polygyny is not practiced and replace with Q. 605 from the Woman's Questionnaire with the word 'HUSBAND'S' replaced with 'WIFE'S' and 'HE' replaced with 'SHE'.
(2) In countries with an active female condom program, the wording of the question should be modified to include reference to both the male and female condom.
(3) High polygyny high HIV prevalence countries may want to add line number of wife from Q. 407 here in the response category.
(4) Coding categories to be developed locally and revised based on the pretest; however, the broad categories must be maintained.
(5) The LAM method coding category should be deleted in countries that do not have a LAM program.

SECTION 5. FERTILITY PREFERENCES

NO.	QUESTIONS AND FILTERS	CODING CATEGORIES	SKIP
501	CHECK 401:<br>CURRENTLY MARRIED OR LIVING WITH A PARTNER ☐ ↓<br>NOT CURRENTLY MARRIED AND NOT LIVING WITH A PARTNER ☐ → 509		509
502	CHECK 439:<br>MAN NOT STERILIZED ☐ ↓<br>MAN STERILIZED ☐ → 509		509
503<br>**(1)**	(Is your (wife/partner)/Are any of your (wives/partners)) currently pregnant?	YES . . . . . 1<br>NO . . . . . 2<br>DON'T KNOW 8	 NO/DON'T KNOW → 505
504<br>**(1)**	Now I have some questions about the future. After the (child/children) you and your (wife(wives)/partner(s)) are expecting now, would you like to have another child, or would you prefer not have any more children?	HAVE ANOTHER CHILD . . . . . 1<br>NO MORE . . . . . 2<br>UNDECIDED/DON'T KNOW 8	1 → 506<br>2, 8 → 509
505<br>**(1)**	Now I have some questions about the future. Would you like to have (a/another) child, or would you prefer not to have any (more) children?	HAVE (A/ANOTHER) CHILD . . . . . 1<br>NO MORE/NONE . . . . . 2<br>SAYS COUPLE<br>CAN'T GET PREGNANT . . . . . 3<br>WIFE (WIVES)/PARTNER(S)<br>STERILIZED . . . . . 4<br>UNDECIDED/DON'T KNOW 8	2, 3, 4, 8 → 509
506<br>**(2)**	CHECK 407:<br>ONE WIFE/ PARTNER ☐ ↓<br>MORE THAN ONE WIFE/ PARTNER ☐ → 508		508
507	CHECK 503:<br>WIFE/PARTNER NOT PREGNANT OR DON'T KNOW ☐ ↓<br>How long would you like to wait from now before the birth of (a/another) child?<br><br>WIFE/PARTNER PREGNANT ☐ ↓<br>After the birth of the child you are expecting now, how long would you like to wait before the birth of another child?	MONTHS . . . . . 1 [ ][ ]<br>YEARS . . . . . 2 [ ][ ]<br>SOON/NOW . . . . . 993<br>COUPLE INFECUND . . . . . 994<br>OTHER ______________ 996<br>(SPECIFY)<br>DON'T KNOW 998	→ 509
508<br>**(2)**	How long would you like to wait from now before the birth of (a/another) child?	MONTHS . . . . . 1 [ ][ ]<br>YEARS . . . . . 2 [ ][ ]<br>SOON/NOW . . . . . 993<br>HE/ALL HIS WIVES/PARTNERS<br>ARE INFECUND . . . . . 994<br>OTHER ______________ 996<br>(SPECIFY)<br>DON'T KNOW 998	

NO.	QUESTIONS AND FILTERS	CODING CATEGORIES	SKIP
509	CHECK 203 AND 205:<br>HAS LIVING CHILDREN ☐ ↓ If you could go back to the time you did not have any children and could choose exactly the number of children to have in your whole life, how many would that be?<br>NO LIVING CHILDREN ☐ ↓ If you could choose exactly the number of children to have in your whole life, how many would that be?<br>PROBE FOR A NUMERIC RESPONSE.	NONE . . . . 00<br>NUMBER . . . . ☐☐<br>OTHER ________________________ 96<br>(SPECIFY)	→ 601<br><br>→ 601
510	How many of these children would you like to be boys, how many would you like to be girls and for how many would it not matter if it’s a boy or a girl?	BOYS GIRLS EITHER<br>NUMBER ☐☐ ☐☐ ☐☐<br>OTHER ________________________ 96<br>(SPECIFY)	

(1) The wording of this question should be modified in countries where polygyny is not practiced.
In Q. 503, change question to 'Is your (wife/partner) currently pregnant?'
In Q. 504, change '(child/children)' to '(child)' and change '(wife/(wives)/partner(s))' to '(wife/partner)'.
In Q. 505, change response category 4 from 'WIFE (WIVES)/PARTNER(S) STERILIZED' to 'WIFE/PARTNER STERILIZED'.

(2) This question should be deleted in countries where polygyny is not practiced.

SECTION 6. EMPLOYMENT AND GENDER ROLES

NO.	QUESTIONS AND FILTERS	CODING CATEGORIES	SKIP
601	Have you done any work in the last seven days?	YES . . . . . . 1<br>NO 2	→ 604 (YES)
602	Although you did not work in the last seven days, do you have any job or business from which you were absent for leave, illness, vacation, or any other such reason?	YES . . . . . . 1<br>NO 2	→ 604 (YES)
603	Have you done any work in the last 12 months?	YES . . . . . . 1<br>NO 2	→ 607 (NO)
604	What is your occupation, that is, what kind of work do you mainly do?	__________ ☐☐<br>__________<br>__________	
605	Do you usually work throughout the year, or do you work seasonally, or only once in a while?	THROUGHOUT THE YEAR . . . . . . 1<br>SEASONALLY/PART OF THE YEAR 2<br>ONCE IN A WHILE 3	
606	Are you paid in cash or kind for this work or are you not paid at all?	CASH ONLY . . . . . . 1<br>CASH AND KIND . . . . . . 2<br>IN KIND ONLY . . . . . . 3<br>NOT PAID 4	
607	CHECK 401:<br>CURRENTLY MARRIED OR LIVING WITH A PARTNER ☐ ↓<br>NOT CURRENTLY MARRIED AND NOT LIVING WITH A PARTNER ☐ →		612
608	CHECK 606:<br>CODE 1 OR 2 CIRCLED ☐ ↓<br>OTHER ☐ →		610
609	Who usually decides how the money you earn will be used: you, your (wife/partner), or you and your (wife/partner) jointly?	RESPONDENT . . . . . . 1<br>WIFE/PARTNER . . . . . . 2<br>RESPONDENT AND WIFE/<br>PARTNER JOINTLY . . . . . . 3<br>OTHER __________ 6<br>(SPECIFY)	
610	Who usually makes decisions about health care for yourself: you, your (wife/partner), you and your (wife/partner) jointly, or someone else?	RESPONDENT . . . . . . 1<br>WIFE/PARTNER . . . . . . 2<br>RESPONDENT AND WIFE/<br>PARTNER JOINTLY . . . . . . 3<br>SOMEONE ELSE . . . . . . 4<br>OTHER __________ 6<br>(SPECIFY)	
611	Who usually makes decisions about making major household purchases?	RESPONDENT . . . . . . 1<br>WIFE/PARTNER . . . . . . 2<br>RESPONDENT AND WIFE/<br>PARTNER JOINTLY . . . . . . 3<br>SOMEONE ELSE . . . . . . 4<br>OTHER __________ 6<br>(SPECIFY)	

NO.	QUESTIONS AND FILTERS	CODING CATEGORIES	SKIP
612	Do you own this or any other house either alone or jointly with someone else?	ALONE ONLY . . . . 1<br>JOINTLY ONLY . . . . 2<br>BOTH ALONE AND JOINTLY . . . . . 3<br>DOES NOT OWN 4	
613	Do you own any land either alone or jointly with someone else?	ALONE ONLY . . . . 1<br>JOINTLY ONLY . . . . 2<br>BOTH ALONE AND JOINTLY . . . . . 3<br>DOES NOT OWN 4	
614	In your opinion, is a husband justified in hitting or beating his wife in the following situations:<br><br>If she goes out without telling him?<br>If she neglects the children?<br>If she argues with him?<br>If she refuses to have sex with him?<br>If she burns the food?	YES NO DK<br><br>GOES OUT . . . . 1 2 8<br>NEGL. CHILDREN . . . 1 2 8<br>ARGUES . . . . 1 2 8<br>REFUSES SEX . . . . . 1 2 8<br>BURNS FOOD 1 2 8	

SECTION 7. HIV/AIDS

NO.	QUESTIONS AND FILTERS	CODING CATEGORIES	SKIP
701	Now I would like to talk about something else. Have you ever heard of an illness called AIDS?	YES . . . . . . 1<br>NO 2	 → 723
702	Can people reduce their chance of getting the AIDS virus by having just one uninfected sex partner who has no other sex partners?	YES . . . . . . 1<br>NO . . . . . . 2<br>DON'T KNOW 8	
703<br>**(1)**	Can people get the AIDS virus from mosquito bites?	YES . . . . . . 1<br>NO . . . . . . 2<br>DON'T KNOW 8	
704	Can people reduce their chance of getting the AIDS virus by using a condom every time they have sex?	YES . . . . . . 1<br>NO . . . . . . 2<br>DON'T KNOW 8	
705<br>**(1)**	Can people get the AIDS virus by sharing food with a person who has AIDS?	YES . . . . . . 1<br>NO . . . . . . 2<br>DON'T KNOW 8	
706<br>**(1)**	Can people get the AIDS virus because of witchcraft or other supernatural means?	YES . . . . . . 1<br>NO . . . . . . 2<br>DON'T KNOW 8	
707	Is it possible for a healthy-looking person to have the AIDS virus?	YES . . . . . . 1<br>NO . . . . . . 2<br>DON'T KNOW 8	
708	Can the virus that causes AIDS be transmitted from a mother to her baby:<br>During pregnancy?<br>During delivery?<br>By breastfeeding?	YES NO DK<br>DURING PREG. . . . . . 1 2 8<br>DURING DELIVERY . . . 1 2 8<br>BREASTFEEDING . . . 1 2 8	
709	CHECK 708:<br>AT LEAST ONE 'YES' ☐ ↓ OTHER ☐		→ 711
710	Are there any special drugs that a doctor or a nurse can give to a woman infected with the AIDS virus to reduce the risk of transmission to the baby?	YES . . . . . . 1<br>NO . . . . . . 2<br>DON'T KNOW 8	
711	CHECK FOR PRESENCE OF OTHERS. BEFORE CONTINUING, MAKE EVERY EFFORT TO ENSURE PRIVACY.		
712	I don't want to know the results, but have you ever been tested to see if you have the AIDS virus?	YES . . . . . . 1<br>NO 2	 → 716
713	How many months ago was your most recent HIV test?	MONTHS AGO . . . . . . ☐☐<br>TWO OR MORE YEARS 95	
714	I don't want to know the results, but did you get the results of the test?	YES . . . . . . 1<br>NO 2	

NO.	QUESTIONS AND FILTERS	CODING CATEGORIES	SKIP
715	Where was the test done? **(3)**<br><br>PROBE TO IDENTIFY THE TYPE OF SOURCE.<br><br>IF UNABLE TO DETERMINE IF PUBLIC OR PRIVATE SECTOR, WRITE THE NAME OF THE PLACE.<br><br>__________________<br>(NAME OF PLACE)	PUBLIC SECTOR<br>GOVERNMENT HOSPITAL . . . . 11<br>GOVT. HEALTH CENTER . . . . 12<br>STAND-ALONE VCT CENTER . . . 13<br>FAMILY PLANNING CLINIC . . . . 14<br>MOBILE CLINIC . . . . 15<br>FIELDWORKER . . . . 16<br>SCHOOL BASED CLINIC . . . . 17<br>OTHER PUBLIC<br>SECTOR __________ 18<br>(SPECIFY)<br><br>PRIVATE MEDICAL SECTOR<br>PRIVATE HOSPITAL/CLINIC/<br>PRIVATE DOCTOR . . . . 21<br>STAND-ALONE VCT CENTER . . . 22<br>PHARMACY . . . . 23<br>MOBILE CLINIC . . . . 24<br>FIELDWORKER . . . . 25<br>SCHOOL BASED CLINIC . . . . 26<br>OTHER PRIVATE MEDICAL<br>SECTOR __________ 27<br>(SPECIFY)<br>OTHER SOURCE<br>HOME . . . . 31<br>CORRECTIONAL FACILITY . . . . 32<br><br>OTHER __________ 96<br>(SPECIFY)	→ 718
716	Do you know of a place where people can go to get tested for the AIDS virus?	YES . . . . 1<br>NO 2	 → 718
717	Where is that? **(3)**<br><br>Any other place?<br><br>PROBE TO IDENTIFY EACH TYPE OF SOURCE.<br><br>IF UNABLE TO DETERMINE IF PUBLIC OR PRIVATE SECTOR, WRITE THE NAME OF THE PLACE.<br><br>__________________<br>(NAME OF PLACE)	PUBLIC SECTOR<br>GOVERNMENT HOSPITAL . . . . A<br>GOVT. HEALTH CENTER . . . . B<br>STAND-ALONE VCT CENTER . . . C<br>FAMILY PLANNING CLINIC . . . . D<br>MOBILE CLINIC . . . . E<br>FIELDWORKER . . . . F<br>OTHER PUBLIC<br>SECTOR __________ G<br>(SPECIFY)<br><br>PRIVATE MEDICAL SECTOR<br>PRIVATE HOSPITAL/CLINIC/<br>PRIVATE DOCTOR . . . . H<br>STAND-ALONE VCT CENTER . . . I<br>PHARMACY . . . . J<br>MOBILE CLINIC . . . . K<br>FIELDWORKER . . . . L<br>OTHER PRIVATE MEDICAL<br>SECTOR __________ M<br>(SPECIFY)<br><br>OTHER __________ X<br>(SPECIFY)	
718	Would you buy fresh vegetables from a shopkeeper or vendor if you knew that this person had the AIDS virus?	YES . . . . 1<br>NO . . . . 2<br>DON'T KNOW 8	

NO.	QUESTIONS AND FILTERS	CODING CATEGORIES	SKIP
719	If a member of your family got infected with the AIDS virus, would you want it to remain a secret or not?	YES, REMAIN A SECRET . . . . 1<br>NO . . . . 2<br>DK/NOT SURE/DEPENDS 8	
720	If a member of your family became sick with AIDS, would you be willing to care for her or him in your own household?	YES . . . . 1<br>NO . . . . 2<br>DK/NOT SURE/DEPENDS 8	
721	In your opinion, if a female teacher has the AIDS virus but is not sick, should she be allowed to continue teaching in the school?	SHOULD BE ALLOWED . . . . 1<br>SHOULD NOT BE ALLOWED . . . . 2<br>DK/NOT SURE/DEPENDS 8	
722<br>**(2)**	Should children age 12-14 be taught about using a condom to avoid getting AIDS?	YES . . . . 1<br>NO . . . . 2<br>DK/NOT SURE/DEPENDS 8	
723	CHECK 701:<br>HEARD ABOUT AIDS ☐ ↓ Apart from AIDS, have you heard about other infections that can be transmitted through sexual contact?<br>NOT HEARD ABOUT AIDS ☐ ↓ Have you heard about infections that can be transmitted through sexual contact?	YES . . . . 1<br>NO 2	
724	CHECK 414:<br>HAS HAD SEXUAL INTERCOURSE ☐ ↓<br>HAS NOT HAD SEXUAL INTERCOURSE ☐		→ 732
725	CHECK 723: HEARD ABOUT OTHER SEXUALLY TRANSMITTED INFECTIONS?<br>YES ☐ ↓<br>NO ☐		→ 727
726	Now I would like to ask you some questions about your health in the last 12 months. During the last 12 months, have you had a disease which you got through sexual contact?	YES . . . . 1<br>NO . . . . 2<br>DON'T KNOW 8	
727	Sometimes men experience an abnormal discharge from their penis.<br>During the last 12 months, have you had an abnormal discharge from your penis?	YES . . . . 1<br>NO . . . . 2<br>DON'T KNOW 8	
728	Sometimes men have a sore or ulcer near their penis. During the last 12 months, have you had a sore or ulcer near your penis?	YES . . . . 1<br>NO . . . . 2<br>DON'T KNOW 8	
729	CHECK 726, 727, AND 728:<br>HAS HAD AN INFECTION (ANY 'YES') ☐ ↓<br>HAS NOT HAD AN INFECTION OR DOES NOT KNOW ☐		→ 732
730	The last time you had (PROBLEM FROM 726/727/728), did you seek any kind of advice or treatment?	YES . . . . 1<br>NO 2	 → 732

NO.	QUESTIONS AND FILTERS	CODING CATEGORIES	SKIP
731	Where did you go? **(3)**<br><br>Any other place?<br><br>PROBE TO IDENTIFY EACH TYPE OF SOURCE.<br><br>IF UNABLE TO DETERMINE IF PUBLIC OR PRIVATE SECTOR, WRITE THE NAME OF THE PLACE.<br><br>____________________<br>(NAME OF PLACE(S))	PUBLIC SECTOR<br>GOVERNMENT HOSPITAL . . . . A<br>GOVT. HEALTH CENTER . . . . B<br>STAND-ALONE VCT CENTER . . . C<br>FAMILY PLANNING CLINIC . . . . D<br>MOBILE CLINIC . . . . E<br>FIELDWORKER . . . . F<br>OTHER PUBLIC<br>SECTOR __________ G<br>(SPECIFY)<br><br>PRIVATE MEDICAL SECTOR<br>PRIVATE HOSPITAL/CLINIC/<br>PRIVATE DOCTOR . . . . H<br>STAND-ALONE VCT CENTER . . . I<br>PHARMACY . . . . J<br>MOBILE CLINIC . . . . K<br>FIELDWORKER . . . . L<br>OTHER PRIVATE MEDICAL<br>SECTOR __________ M<br>(SPECIFY)<br><br>OTHER SOURCE<br>SHOP . . . . N<br>OTHER __________ X<br>(SPECIFY)	
732<br>**(2)**	If a wife knows her husband has a disease that she can get during sexual intercourse, is she justified in asking that they use a condom when they have sex?	YES . . . . 1<br>NO . . . . 2<br>DON'T KNOW 8	
733	Is a wife justified in refusing to have sex with her husband when she knows her husband has sex with other women? **(4)**	YES . . . . 1<br>NO . . . . 2<br>DON'T KNOW 8	

(1) If Qs. 703, 705 and/or 706 do not apply to the local context, replace the question using a specific local misconception. At least two questions related to misconceptions are needed.
(2) The question may be deleted in countries with a very low HIV prevalence.
(3) Coding categories to be developed locally and revised based on the pretest; however, the broad categories must be maintained.
(4) In polygynous societies, the phrase 'other women' should be replaced by the phrase 'women other than his wives.'

SECTION 8. OTHER HEALTH ISSUES

NO.	QUESTIONS AND FILTERS	CODING CATEGORIES	SKIP
801 **(1)**	Some men are circumcised, that is, the foreskin is completely removed from the penis. Are you circumcised?	YES . . . . . 1<br>NO . . . . . 2<br>DON'T KNOW 8	 NO, DON'T KNOW → 805
802 **(1)**	How old were you when you got circumcised?	AGE IN COMPLETED YEARS . . . . [ ][ ]<br><br>DURING CHILDHOOD (<5 YEARS) 95<br>DON'T KNOW 98	
803 **(1)**	Who did the circumcision?	TRADITIONAL PRACTITIONER/ FAMILY/FRIEND . . . . . 1<br>HEALTH WORKER/PROFESSIONAL 2<br>OTHER . . . . . 3<br>DON'T KNOW 8	
804 **(1)**	Where was it done?	HEALTH FACILITY . . . . . 1<br>HOME OF A HEALTH WORKER/ PROFESSIONAL . . . . . 2<br>CIRCUMCISION DONE AT HOME . . . 3<br>RITUAL SITE . . . . . 4<br>OTHER HOME/PLACE . . . . . 5<br>DON'T KNOW 8	
805	Now I would like to ask you some other questions relating to health matters. Have you had an injection for any reason in the last 12 months?<br><br>IF YES: How many injections have you had?<br><br>IF NUMBER OF INJECTIONS IS 90 OR MORE, OR DAILY FOR 3 MONTHS OR MORE, RECORD '90'.<br><br>IF NON-NUMERIC ANSWER, PROBE TO GET AN ESTIMATE.	NUMBER OF INJECTIONS . . . [ ][ ]<br><br>NONE 00	 <br>NONE → 808
806	Among these injections, how many were administered by a doctor, a nurse, a pharmacist, a dentist, or any other health worker?<br><br>IF NUMBER OF INJECTIONS IS 90 OR MORE, OR DAILY FOR 3 MONTHS OR MORE, RECORD '90'.<br><br>IF NON-NUMERIC ANSWER, PROBE TO GET AN ESTIMATE.	NUMBER OF INJECTIONS . . . [ ][ ]<br><br>NONE 00	 <br>NONE → 808
807	The last time you got an injection from a health worker, did he/she take the syringe and needle from a new, unopened package?	YES . . . . . 1<br>NO . . . . . 2<br>DON'T KNOW 8	
808	Do you currently smoke cigarettes?	YES . . . . . 1<br>NO 2	 NO → 810
809	In the last 24 hours, how many cigarettes did you smoke?	NUMBER OF CIGARETTES . . . . . [ ][ ]	
810	Do you currently smoke or use any (other) type of tobacco? **(2)**	YES . . . . . 1<br>NO 2	 NO → 812
811	What (other) type of tobacco do you currently smoke or use? **(2)**<br><br>RECORD ALL MENTIONED.	PIPE . . . . . A<br>CHEWING TOBACCO . . . . . B<br>SNUFF . . . . . C<br><br>OTHER ______ X<br>(SPECIFY)	

NO.	QUESTIONS AND FILTERS	CODING CATEGORIES	SKIP
812	Are you covered by any health insurance? **(3)**	YES . . . . 1<br>NO 2	 → 814
813	What type of health insurance are you covered by? **(3)**<br><br>RECORD ALL MENTIONED.	MUTUAL HEALTH ORGANIZATION/<br>COMMUNITY-BASED HEALTH<br>INSURANCE . . . . A<br>HEALTH INSURANCE THROUGH<br>EMPLOYER . . . . B<br>SOCIAL SECURITY . . . . C<br>OTHER PRIVATELY PURCHASED<br>COMMERCIAL HEALTH INSURANCE D<br>OTHER ______________________ X<br>(SPECIFY)	
814	RECORD THE TIME.	HOUR . . . . [ ][ ]<br>MINUTES . . . . [ ][ ]	

(1) Question may be omitted depending on the practice of male circumcision in specific countries. Translation of circumcision should indicate removal of the foreskin and not merely coming of age ceremonies.
(2) Add local terms.
(3) If a health service prepayment plan or other types of plans are available in the country, add those types of plans to the question.

INTERVIEWER'S OBSERVATIONS

TO BE FILLED IN AFTER COMPLETING INTERVIEW

COMMENTS ABOUT RESPONDENT:

____

____

____

____

____

____

COMMENTS ON SPECIFIC QUESTIONS:

____

____

____

____

____

ANY OTHER COMMENTS:

____

____

____

____

____

SUPERVISOR'S OBSERVATIONS

____

____

____

____

____

____

____

NAME OF SUPERVISOR: ____________________ DATE: ____________________

EDITOR'S OBSERVATIONS

____

____

____

____

____

NAME OF EDITOR: ____________________ DATE: ____________________

DHS
TOOLKIT
